# Over-Activation of Minichromosome Maintenance Protein 10 Promotes Genomic Instability in Early Stages of Breast Cancer

**DOI:** 10.7150/ijbs.69344

**Published:** 2022-05-29

**Authors:** Muhammad Jameel Mughal, Kin Iong Chan, Ravikiran Mahadevappa, Sin Wa Wong, Kit Cheng Wai, Hang Fai Kwok

**Affiliations:** 1 Cancer Centre, Faculty of Health Sciences, University of Macau, Avenida de Universidade, Taipa, Macau SAR; MoE Frontiers Science Center for Precision Oncology, University of Macau, Avenida de Universidade, Taipa, Macau SAR; 2 Centre for Precision Medicine Research and Training, Faculty of Health Sciences, University of Macau, Avenida de Universidade, Taipa, Macau SAR; Department of Pathology, Kiang Wu Hospital, Macau SAR

**Keywords:** MCM10, DNA replication, DNA damage response, genomic instability, breast cancer

## Abstract

Genomic instability is considered as one of the key hallmark during cancer development and progression. Cellular mechanisms, such as DNA replication initiation, DNA damage and repair response, apoptosis etc are observed to block progression of genomic instability and thereby induce protective effects against cancer. DNA replication initiation protein MCM10 has been previously observed to have an increased expression in different cancer subtypes. However, MCM10 association with genomic instability, cancer development and its relevant mechanisms remain unknown. Here, using a breast cancer model, we observe a significant association of MCM10 with the degree of clinical aggressiveness in breast cancer patients. By overexpression of MCM10, we observed that MCM10 promotes tumorigenic properties in immortal non-tumorigenic mammary cells by increasing proliferation, shortening the cell cycle, and promoting tumorigenic characters in *in-vivo* mimicking conditions. Furthermore, overexpression of MCM10 is found to induce accumulation of ssDNA followed by overexpression of ssDNA binding protein RPA2. Mesenchymal markers such as up-regulation of Vimentin, transcription factor Snail and Twist2, and the down-regulation of E-cadherin were observed in MCM10 overexpression cells. Overall, the findings of this study revealed a novel mechanism by which MCM10 promotes genomic instability and breast cancer progression, and effectively differentiates the active degree of breast cancer aggressiveness. Thus, MCM10 has the potential to be a clinically useful biomarker as well as a therapeutic target for future breast cancer treatment.

## Introduction

Genomic instability within healthy cells is considered one of the earliest events during cancer development. Genomic instability is shown to enhance the genomic heterogeneity leading to aggressive tumor behavior and resistance to cancer treatment therapies 1. DNA replication is essential, one of the key events which unchecked results in the accumulation of harmful mutations that lead to cancer development 2. Multiple mechanisms ensure the accuracy of DNA replication. Once an error in replication is detected the feedback mechanism such as termination of DNA replication initiation, activation of DNA damage and repair response, activation of apoptosis etc., are observed to block progression of genomic instability and thereby induce protective effects against cancer. Hence, multiple endogenous factors that can potentially sense replication stress and genomic instability are worth exploring.

Cell proliferation is a crucial element of development that is disrupted in many malignancies. The replication of DNA, which occurs during the S phase of the cell cycle, is a critical component of cell proliferation 3. A major event in the DNA replication is the conversion of inactive Pre-replication complex (RC) to active RC by linking CMG helicase complex 4 to DNA polymerase. In eukaryotes, Minichromosome maintenance protein 10 (MCM10) is a unique replication initiator protein that links CMG helicase complex 5 to DNA polymerase ε. thereby activating helicase as well as DNA synthesis on the site leading to 'Origin firing' 6. Previously, MCM10 has also been shown in many experimental models to interact with ssDNA and various key DNA replication proteins such as CDC45, MCM2-7, DNA polymerase, PCNA, and GINS 7-11, all of which are required for accurate cell replication and proliferation. Thus, MCM10 expression levels could be one of the barriers in preventing replication catastrophe that may lead to genomic instability and cancer development/progression. Significantly higher expression of MCM10 has been observed in patients with different types of cancers such as lung cancer, cervical cancer, urothelial carcinoma, gastric cancer, esophageal cancer and prostate cancer 12. However, the mechanism by which MCM10 drives genomic instability and cancer progression remains unexplored. Here we hypothesized that overexpression of MCM10 causes increased proliferation by activating RCs leading to reduce DNA replication accuracy as well as increase in accumulation of single strand DNA (ssDNA). Excess ssDNA may lead to the activation of DNA damage response (DDR) pathway by recruiting multiple proteins that regulate cell cycle progression, 13. The persistence of these lesions during DNA replication has been shown to induce mutations, copy number alterations and chromosomal rearrangements 14, 15 and found to be associated to the initial stages of tumorigenesis 16.

In the present study, using breast cancer models we explored the relevance of MCM10 expression levels with breast cancer aggressiveness and validated our results in clinical Breast cancer (BC) patient samples. We established MCM10 overexpression (MCM10 OE) in MCF10A and NIH-3T3 cell lines to understand the role of MCM10 in tumorigenesis. In clinical samples and in database analysis we observed a significant increase in MCM10 expression compared to healthy individuals. *In-vitro* cell culture models with overexpression of MCM10 showed an increase in cell proliferation via significant increase in AKT (Phosphatidylinositol-3-Kinase and Protein Kinase B) signaling pathway. Overexpression of MCM10 also leads to the accumulation of ssDNA, however, we were unable to observe either DNA damage response or apoptosis in these models. Further, MCM10 overexpressing cells showed a significant increase in tumorigenic characters in immortal non-tumorigenic MCF 10A mammary cells. In our study, we report potential links to MCM10 expression levels and breast cancer aggressiveness as well as a novel mechanism by which MCM10 can accumulate DNA damage without inducing stress signals.

## Materials and Methods

### Extraction of clinical and microarray gene expression data from breast cancer patient cohorts

GENT2 (Gene Expression database of Normal and Tumor tissues 2) database that contains gene expression data of 68,000 clinical samples as well as cell lines was used to retrieve the expression data of MCM10, RPA1, RPA2, RPA3, ATR and CHEK1 genes as previously described 17.

We first retrieved MCM10 gene expression data from 1829 BC patient samples having a different degree of BC malignancies based on molecular subtypes (Luminal A, N=379, Luminal B, N=244, HER2, N=230, Triple negative Breast cancer (TNBC), N= 251) and as well as tumor grades (Grade 1, N=82, Grade 2, N=193, and Grade 3, N=450) From GENT2 database. MCM10 expression data was also retrieved from 4 breast cancer cell lines (MDA231, N=55, MCF7, N=49, T47D, N=21, and SKBR3, N=14) and one normal cell line (MCF10A, N= 13). Relevant information about datasets IDs, sample IDs and subtype expression are presented in Supplementary Table S3, Table S4 and Table S5. Expression pattern of MCM10, RPA1, RPA2, RPA3, ATR and CHEK1 in Grade 1, Grade 2 and Grade 3 was observed using Mann Whitney test and boxplot were made using R script. Patients with survival status available were used for survival analysis (N=502). Kaplan-Meier analyses were performed by dividing patients into two groups, the median expression level was used as the cut-off point. A value of p< 0.05 was considered significant (*), while p< 0.01 was considered markedly significant (**). ROC curve analysis was performed and ROC curves were generated to examine how well the MCM10 expression level can discriminate between different degrees of breast cancer malignancy using Graph Pad Prism 7 software.

### Patients' specimens collection

A total of eighteen patient samples (six Grade 1, six Grade 2 and six Grade 3) were obtained from breast cancer patients enrolled at Kiang Wu Hospital. The tumors were graded by Bloom & Richardson grading system. All clinical samples were collected after written informed consent was obtained from the patients. Studies were performed with the approval of the Medical Ethics Committee of Kiang Wu Hospital and the Faculty of Health Sciences University of Macau.

### Hematoxylin Eosin (HE) and Immunohistochemical (IHC) staining of patient specimens

The patients samples obtained from Kiang Wu Hospital were fixed and dehydrated in ethanol, then embedded in paraffin wax. Thin sections (5 μm) of tissue were sliced and mounted on glass slides. The tissue sections were then stained with hematoxylin and eosin staining. The histological organization of the tissues was observed, and imaging was achieved with Carl Zeiss Axio Imager 2 Microscope (Zeiss). For IHC, the tissue sections were de-paraffinized overnight in xylene and rehydrated using ethanol gradient. The samples were immersed in methanol containing 0.3% hydrogen peroxide and epitope retrieval with the Pre-treatment (PT) module using TEG buffer. The sections were then washed in 1% BSA and incubated overnight with the primary antibodies in 0.1% BSA (antibodies details and dilution rates are presented in Supplementary Table S1). After washing with PBS, the sections were incubated with respective HRP-conjugated secondary antibodies. The sections were then developed using DAB (ZsBio, Beijing, China) and counterstained with Meiers haematoxylin for 2 min followed by de-hydration in an ethanol gradient and xylene. Mounting was performed with Eukitt mounting medium (Merck) and imaging was achieved with the same Carl Zeiss Axio Imager 2 Microscope. IHC staining intensity score was computed using IHC Profiler plugin of ImageJ processing software 18 as previously described 19

### Cell lines and culture

Human mammary epithelial (MCF10A), immortalized mouse embryonic fibroblast (NIH3T3) and breast cancer cell lines (MCF-7, T47D, MDA-231 SKBR3) were procured from ATCC MCF10A cells were cultured and maintained in a 1:1 mixture of Dulbecco's Modified Eagle Medium and Nutrient Mixture F-12 medium (DMEM/F12) (Gibco, Carlsbad, CA) supplemented with 5% horse serum (HS), 0.5 μg/ ml hydrocortisone (Sigma-Aldrich, St. Louis, MO, USA), 20ng/ ml epidermal growth factor EGF (Peprotech, Rocky Hill, NJ, USA), and 10 μg/ ml insulin (Sigma-Aldrich, St. Louis, MO, USA) and 100 ng/ ml cholera toxin (Sigma-Aldrich). NIH3T3, MCF-7, T47D, MDA-231 and SKBR3 cell lines were cultured and maintained in DMEM (Gibco, Carlsbad, CA) supplemented with fetal bovine serum (Gibco) to a final concentration of 10%, 1% Pen/Strep (Gibco), and 1% L-Glutamine (Gibco).The entire cell lines were cultured in CO2 (5%) incubator at 37 °C with 95% humidity.

### MCM10 overexpression

For MCM10 overexpression, Phoenix packaging cells were transfected with pBABE-puro (#1764 Addgene, Cambridge, MA) vector containing MCM10-3xFLAG or empty vector using Lipofectamine 3000 according to manufacturer's protocol. After 48 h of transfection, the virus-containing medium was collected, filtered through a 0.45-μm filter and added to the cells of interest in the presence of 6 μg/ml of polybrene (Millipore, Billerica, Massachusetts). The lentivirus-infected cells were selected with 2 μg/ ml puromycin (InvivoGen, San Diego, CA). Transduction efficiency was confirmed by qPCR and Western blotting.

### Cell synchronization

Cell synchronization was performed by double thymidine block as previously described 20. In detail, asynchronized cells in equal quantity were seeded in cell culture dish and incubated at 37 °C overnight. The next day thymidine was added to a final concentration of 2 mM and cells were incubated for 18 hours. Thymidine was removed by washing cells with 1X PBS. Cells were then incubated with fresh medium for 9 hours at 37 °C. Cells were again incubated with thymidine for 18 hours to a final concentration of 2 mM for the second round. Finally, cells were washed with 1X PBS, and fresh medium was added. Cells were collected at different time points for different analysis as described below.

### Cell cycle analysis

Cell cycle flow cytometry analysis was done as previously described 21. Briefly, at the indicated time, cells were trypsinized, washed twice with 1×PBS, and pelleted by low-speed centrifugation. Pellet was resuspended with 70% ethanol for 30 min at 4°C. Cells were spun down and were incubated with the DNA-binding dye propidium iodide (PI) and 50 ug/ml PI in deionized water for 1 hour at room temperature. Finally, cells were analyzed by a BD Accuri™ C6 Plus Flow Cytometer.

### Apoptosis assay

Apoptosis was assessed via flow cytometric analysis of control and MCM10 overexpression cells that were stained with FITC-Annexin V and PI using the Annexin V-FITC apoptosis Detection kit according to the manufacturer's protocol (Alexa Fluor 488 Annexin V/Dead Cell Apoptosis Kit).

### Cell proliferation and scratch wound healing migration assays

Cell proliferation assays were performed by seeding cells at a density of 2.25 x 10^4^ cells per well of a 12-well plate in full growth media. All cultures were performed in quadruplicates (n=3). Scratch wound healing migration assay was performed by growing cells to confluency in 96-well plates (ImageLock; Essen Bioscience, Ann Arbor, MI) in a standard cell culture incubator. The 96-pin wound-making tool (WoundMaker; Essen Bioscience) was used to create a precise and reproducible wound in each well simultaneously. Cell proliferation and migration were monitored by Incucyte Zoom (Incucyte, Essen Bioscience). This allows an automated and non-invasive method of monitoring live cells in culture.

### Senescence-associated β-galactosidase staining

Cells were seeded in 6-well plates. At the indicated time medium was removed and cells were rinsed with PBS and fixed with 1x fixative solution provided by senescence β-galactosidase staining kit (9860, Cell Signaling Technology) for 15 minutes. The fresh β-galactosidase staining solution was prepared according to manufacturer's instructions. Cells in each well were stained with 1 mL staining solution after being washed with PBS twice. The process of staining was accomplished after incubation at 37°C in a dry incubator overnight. The β-galactosidase positive cells were considered as senescent cells and counted in at least 4 randomly chosen fields using EVOS® FL Cell Imaging System.

### Single cell gel electrophoresis (Comet assay)

Lysis buffer, alkaline solution, and electrophoresis running solution were prepared and chilled at 4ºC thoroughly before performing the assay. The assay was performed using ab238544 Comet Assay Kit (3- well slides) according to the manufacturer's instructions. In brief, Comet agarose was heated to 90-95ºC in a water bath for 20 mins, or until agarose liquified and then cooled by transferring the bottle to a 37ºC water bath for 20 mins. A volume of 75 μL of Comet Agarose was added per well onto the Comet Slide to create a base layer, and complete well coverage was achieved by spreading the solution over the well with the pipette tip. The slide was maintained horizontally and transferred to 4ºC for 15 mins. Cell suspension was prepared by washing cells pellet with ice-cold PBS (without Mg2+ and Ca2+) once and resuspending them at a density of 1x10^5^ in ice-cold PBS. Cell samples were combined with Comet Agarose at 1/10 ratio (v/v), mixed well by pipetting, and immediately 75μL/well cell suspension was transferred onto the top of the Comet agarose base layer. The slide was transferred to a small basin/container containing pre-chilled lysis buffer (~25 mL/slide) and then immersed in the buffer for 30-60 mins at 4ºC in the dark. The slide was maintained horizontally and carefully transferred to an alkaline solution to a horizontal electrophoresis chamber. The chamber was filled with cold alkaline electrophoresis solution until the buffer level covered the slide. A voltage of 1 volt/cm was applied for 30 mins. After completion, the slide was carefully transferred to a clean, small basin/container containing pre-chilled deionized water and immersed for 2 mins. The water was aspired and replaced with cold 70% ethanol for 5 mins. Then the slide was removed from 70% ethanol and allowed to air dry. 1X Vista Green DNA Staining Solution was prepared by diluting the provided stock 1/10000 in TE Buffer (10 mM Tris, pH 7.5, 1 mM EDTA). 100 μL/well of diluted Vista Green DNA Dye was added to the slide and incubated at room temperature for 15 mins. Finally, the results were monitored by epifluorescence microscopy using a FITC filter.

### Transwell cell migration assay

Cell Biolabs' CytoSelect™ Cell Migration Assay was used following the manufacturer's instruction. Cells were counted and placed on a polycarbonate membrane or basement membrane inserts in a 24-well plate containing full medium in triplicates. 1x10^6^ cells were plated per insert and were incubated for 24h at 37ºC. The cells that did not migrate were removed from the top of the inserts, and the cells that migrated through the inserts were fixed, stained and imaged using EVOS® FL Cell Imaging System. Cells were also extracted using extraction solution and transferred to 96 well plate, and OD 560nm was also measured using Thermo Scientific™ Multiskan™ GO Microplate Spectrophotometer.

### Clonogenic assay

In the anchorage-dependent colony formation assay, 1000 cells/ well were seeded in a 6 well plate. After 15 days, cells were fixed and stained with 0.5% crystal violet solution (made in 25% methanol and stored at room temperature) for 10 min and photographed. Colonies were counted using densitometry software clono-counter as described previously 22.

### Soft agar colony formation assay

In the anchorage-independent colony formation assay, a 6-well cell culture plate was coated with 2 ml of a solution with 0.6% sterile low melting-point agarose (Lonza, Basel, Switzerland) in DMEM medium supplemented with 10% FBS. After the agar layer was solidified, the upper layer was prepared by mixing a solution of 0.3% agarose with cell suspension of 2.5x10^5^ cells/ml. After the upper layer was solidified, it was then covered by 1 ml of complete medium. Cells were cultured for two weeks under standard conditions, supplemented with 150 µl of complete medium 2-3 times per week. Colonies greater than 50 µm were counted and photographed at 20x magnification under a microscope (Leica M165FC stereomicroscope) and analyzed using ImageJ 1.46r software. At least two independent experiments were performed in triplicates.

### Spheroid formation assay

Spheroids were generated as previously described by Zhang, et al. 2019. Briefly, Agarose (0.5%) was pre-coated on 100 mm culture plates. Next, 50000 cells were seeded on the pre-coated plates and cultured in a medium supplemented with 10% FBS for 8-10 days at 37 °C in a 5% CO2 incubator. Spheroids were photographed using EVOS® FL Cell Imaging System. To calculate the number, spheroids were collected by centrifugation and re-seeded in 6-well plates and incubated for 12 h for cell attachment. The attached spheroids were then stained with 0.5% crystal violet and the number of spheroids were calculated by ImageJ software.

### Immunofluorescence and Confocal Microscopy

The details of all antibodies used in the study are mentioned in Supplementary Table S1. Immunofluorescence assay was performed as per Abcam's immunofluorescence protocol. Briefly, cells were cultured on coverslips (15 mm) in 12-well plates until they reached 70-80% confluency. Adherent cells were fixed in 4% paraformaldehyde (PFA) for 15 min, followed by permeabilization with 0.05% Triton X-100 for 2 min. Non-specific sites were blocked by incubation in 0.1% BSA in PBS for 60 min. Cells were incubated overnight at 4 °C with the specified primary antibodies diluted in the blocking buffer. After that, cells were washed and incubated with either Alexa Fluor 488 or 555 conjugated goat anti-rabbit and goat anti-mouse IgG in a blocking buffer for 60 min at room temperature. Finally, coverslips were mounted using the anti-fade reagent Fluoro-gel II with DAPI. Confocal microscopic analyses were performed using Zeiss LSM 710 confocal microscope and images were acquired and analyzed using the ZEN 2012 image software.

### RNA extraction and quantitative real time PCR (qRT-PCR)

Whole RNA was isolated from the experimental cell lines using Qiagen RNeasy Mini Kit following the manufacturer's instructions. A total of 1µg of RNA was reverse transcribed to cDNA using cDNA Reverse Transcription Kit in a total reaction volume of 20μl on BioRad C1000 TouchTM Thermal Cycler. qRT-PCR reactions were performed in a total volume of 20μL using FastStart Universal SYBR Green Master mix (Cat No: 04913914001, Roche Applied Science, Germany), at the following thermocycler program; Initial denaturation at 95 ℃ for 10 min, followed by 44 cycles of “10 s at 95 ℃ and 30 s at melting temperature (Tm) of a specific primer pair”, and melt curve analysis by 10 s at 95 ℃, and 72 ℃ for 10 s, using Thermal Cycler (Step One Plus, Applied BioSystems, USA). GAPDH was used as an internal control. Primers information is provided in Supplementary Table S2. The qRT-PCR data were analyzed and fold changes in expressions were calculated using the 2-ΔΔCt calculation method described by (Livak and Schmittgen 2001).

### Western Blot

The experimental cell lines were harvested and the cell suspension was prepared in RIPA buffer (150 mM NaCl, 50 mM Tris, 5 mM EDTA, 0.5% sodium deoxycholate, 1% Triton X-100, 0.1% SDS) topped with freshly prepared protease and phosphatase inhibitors (Roche Applied Science, Indianapolis, IN) . The whole-cell lysate was sonicated and later centrifuged to extract the proteins. The quantity of extracted proteins was measured by BCA protein assay kit (ThermoFisher Scientific, Waltham, MA) and comparable quantities of these proteins were laden onto 4-12% SDS-polyacrylamide (SDS-PAGE) gels followed by their electrophoretic transfer onto the PVDF film by Novex iBlot transfer stack from ThermoFisher Scientific on iBlot gel transfer instrument (ThermoFisher Scientific). PVDF film holding the transferred proteins was further blocked for 1 hour by 5% bovine serum albumin (BSA) at room temperature. The target proteins were identified by overnight incubation of PVDF membrane with primary antibodies at 4 °C. The blots were later developed using secondary antibodies conjugated with horseradish peroxidase (HRP) and exposed using Immobilon Western Chemilum HRP substrate (Merck, Darmstadt). The blots were further seen using ChemiDoc Touch Imaging System (BioRad Laboratories, Hercules, CA). Antibodies information is provided in Supplementary Table S1.

### Statistical analysis

Microsoft Excel, statistics17, GraphPad Prism 7 and R software were utilized for statistical analysis. All experiments were conducted at least thrice. The significance of the difference between two groups was analyzed by variance analysis (Mann Whitney test: Patients datasets acquired from GENT2 database, Student t-test: Experimental data), and results are expressed as the mean value with standard deviation. A value of p< 0.05 was considered significant (*), while p< 0.01 was considered markedly significant (**).

## Results

### MCM10 expression levels are directly proportional to the degree of aggressiveness in breast cancer

**Fig 1:**
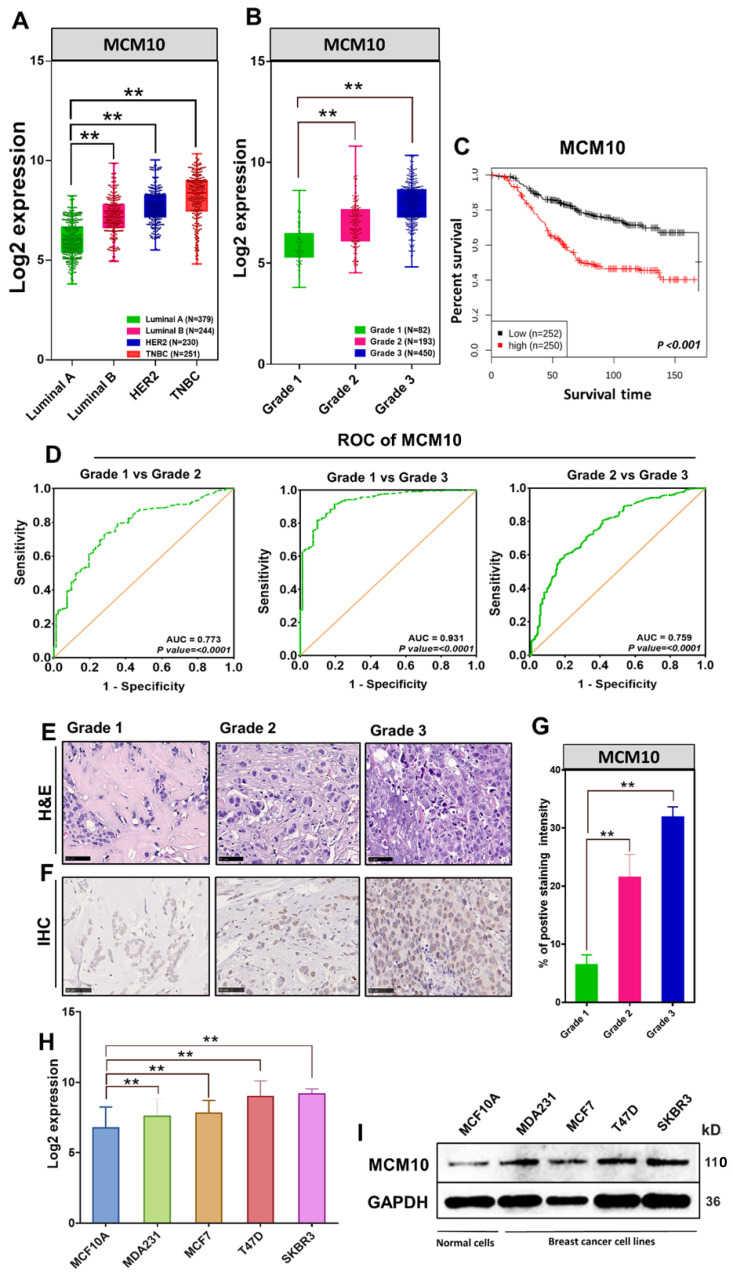
MCM10 expression in BC patients' specimens with different degrees of malignancy as well as in BC cell lines, survival status analysis and MCM10 expression sensitivity and specificity in discriminating malignancies of different grades. A) Box plot showing comparative expression of MCM10 in Luminal A, Luminal B, HER2, TNBC Breast cancer patients in combined BC patient's cohorts (n=1104). B) Box plot showing comparative expression of MCM10 in Grade 1, Grade 2 and Grade 3 BC patients in combined BC patient's cohorts (n=725), indicating consistent and significantly higher expression of MCM10 in tumors with a higher grade. C) Kaplan-Meier plot showing the proportion of patient survival for those with low or high MCM10 expression levels stratified by median value in combined BC cohorts (n=502). High expression of MCM10 was significantly associated with lower patient survival in BC patients. D) ROC curve analysis to validate MCM10 gene expression in characterization among different grades BC patient's samples. The total area under the curve (AUC) for MCM10 in Grade1 vs Grade 2 (AUC= 0.773, P=0.0001), Grade 1 vs Grade 3 (AUC= 0.931, P=0.0001) and Grade 2 vs Grade 3 (AUC= 0.759, P=0.0001) was observed, clearly indicating that MCM10 is potentially able to discriminate accurately among different grades breast cancer. E) Representative microscopic images of H & E in different grades of BC tissue taken at 40X. F). IHC staining of MCM10 in different grades of breast cancer, showing higher expression of MCM10 in tumors with higher grade tissue samples. Images were taken at 40X. G) Bar graph indicating expression of MCM10 in 4 BC cell lines (MDA231, MCF7, T47D, SKBR3) compared to normal cell line (MCF10A). H) Western blot analysis showing the protein expression level of MCM10 in normal MCF10A and MDA231, MCF7, T47D, SKBR3 BC cell lines, MCM10 protein expression was observed high in all BC cell lines compared to normal cell line. The significance of the difference between two groups was analyzed by variance analysis (Mann Whitney test: Patients datasets acquired from GENT2 database, Student t-test: Experimental data), and results are expressed as the mean value with standard deviation. A value of p< 0.05 was considered significant (*), while p< 0.01 was considered markedly significant (**).

Previously we have reported that MCMs expression levels remain low in healthy tissues and are often found to be high in a variety of human cancers 12. As a next step, we compared MCM10 expression in multiple cancer tissues, cancer cell lines versus normal tissue and normal cell lines respectively (Supplementary Fig. S1 and S2). These findings suggested the possible involvement of MCM10 in cancer progression. To explore the relevance of MCM10 with Breast cancer (BC) and associated mechanisms, we first retrieved MCM10 gene expression data of 1829 BC patient samples from GENT2 database and looked at MCM10 expressions patterns in patients having different degrees of BC malignancies based on molecular subtypes (Luminal A, Luminal B, HER2, Triple negative Breast cancer (TNBC) and as well as tumor grades (Grade 1, Grade 2, and Grade 3). Interestingly, MCM10 expression was found consistently and significantly higher in tumors with a higher malignancy and a higher tumor grade (Fig. 1A-B). To investigate whether MCM10 confer prognostic value to BC patients, we carried out Kaplan-Meier survival analysis in patients with survival statuses available. Patients with tumors expressing lower MCM10 had significantly longer survival than patients with tumors expressing higher MCM10 (Fig. 1C). To verify MCM10 expression, discriminating among different grades BC patient's samples, we performed receiver operating characteristic (ROC) curve analysis. ROC analysis clearly indicated that MCM10 is able to discriminate accurately among different grades BC (Fig. 1D). To validate the association of MCM10 expression with different degrees of malignancies in BC, we performed a collaborative study with Kiang Wu hospital Macau (a local hospital in Macau SAR) and obtained BC patient specimens having a different degree of aggressiveness. Specimens were stratified based on Bloom-Richardson system and stained with H&E (Fig. 1E) as well as with IHC staining (Fig. 1F). IHC staining of MCM10 showed relatively higher and distinct expression of MCM10 in different grades of tumors, i.e. higher the tumor grade, higher the MCM10 expression (Fig. 1F-G). These interesting results further motivated us to look at the expression pattern of MCM10 in normal and BC cell lines. Again, we retrieved MCM10 expression data of different BC cell lines (MDA231, MCF7, T47D, and SKBR3) and normal cell line (MCF10A) from GENT2 (Gene Expression database of Normal and Tumor tissues 2) database and validated observed results by western blotting. Log2 expression value showed significantly high expression of MCM10 in all 4 BC cell lines compared to normal cell line (Fig. 1H). A similar protein expression pattern of MCM10 was also seen in western blot analysis (Fig. 1I).

### MCM10 overexpression induces significant increase in proliferation of immortal non-tumorigenic MCF 10A mammary cells

**Fig 2:**
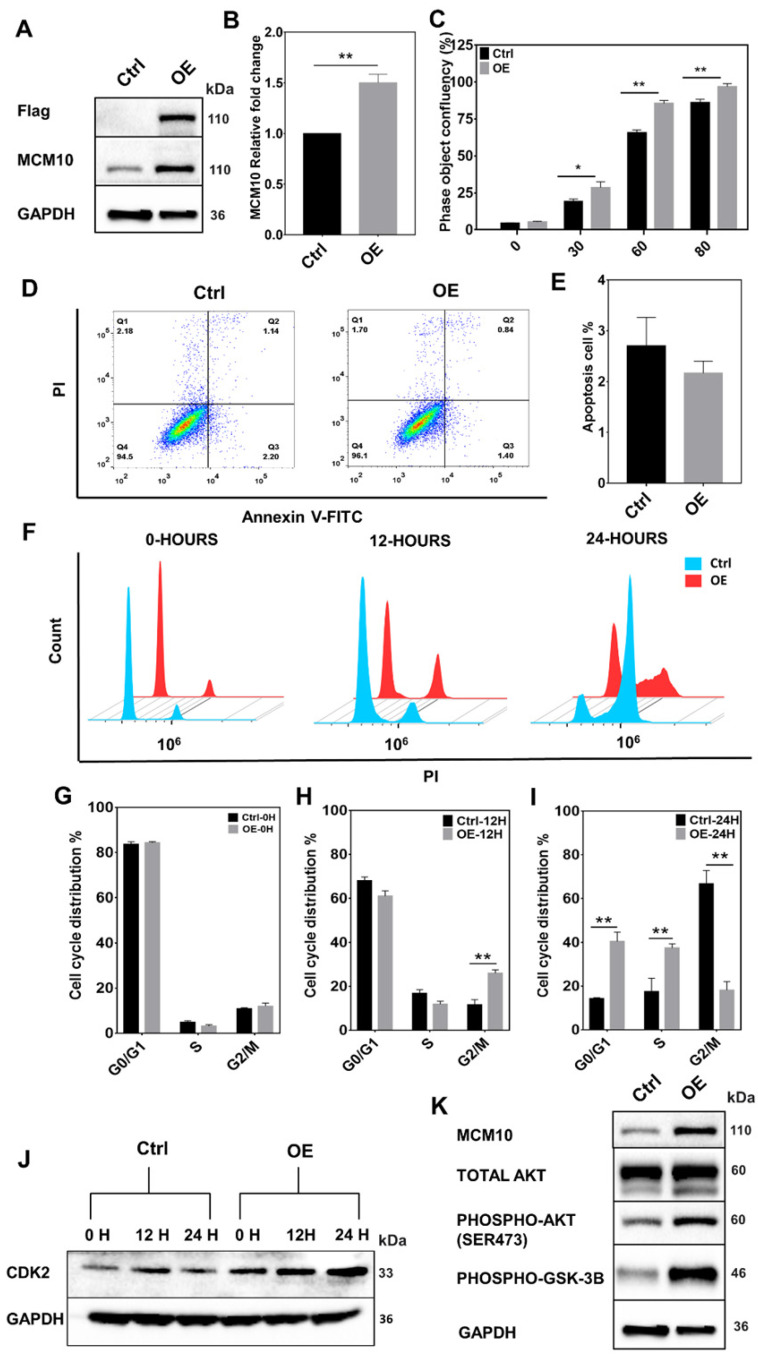
High expression of MCM10 in MCF10A cells. A) MCM10 expression monitored by 3x-FLAG and MCM10 using western blotting. B) mRNA quantification showing significant increase in MCM10 mRNA expression in MCF10A-OE cells compared to control. C) Bar graph showing a significant increase in cell proliferation in MCF10A-OE cells at different time points compared to control, monitored and analyzed using Incucyte ZOOM analysis software. D) Scattered analysis of apoptosis between control and MCF10A-OE cells, monitored by flow cytometer. E) Apoptotic percentage of control and MCF10A-OE groups showing no difference. F) Representative images of cell cycle analysis at 0, 12 and 24 hours after serum starvation by flow cytometer. G) Bar graph of Cell cycle analysis at 0 hours, H) at 12 hours and I) at 24 hours. Cell cycle analysis showed a relatively decreased percentage of cells in the S-phase and an increased percentage of cells in the G2/M phase at 12 hours. Increased percentage of cells in G0/G1, S-phase and decreased percentage of cells in G2/M phase in MCF10A-OE cells compared to control also been observed at 24 hours suggesting that MCF10A-OE cells might relatively shorten their duplication time. J) Protein expression of CDK2 (a S-phase cell cycle marker) in control and MCF10A-OE cells at different time points. K) Protein expression of AKT-pathway in MCF10A-OE cells and control groups, indicating increased proliferation in MCF10A-OE by modulating AKT signaling pathway. The student t-test was used to analyze the difference between the two groups and data are presented as mean values ± s.d. Value of p< 0.05 was considered significant (*), while p< 0.01 was considered markedly significant (**).

Cell proliferation is one of the most essential features of development and goes misregulated in many malignancies. A central part of cell proliferation is the replication of DNA that happens during S phase of the cell cycle. Recent research into the regulation of replication timing has found that MCM10 mutations are linked to extensive replication timing variations 3. It has been reported that cells from a single patient with MCM10 mutations showed replication time variability in 46 percent of the genome, compared to RIF1 knockdown (a known modulator of replication timing) 3. In addition, MCM10 has been shown to play an important role in DNA unwinding 10. Considering these findings, we hypothesized that MCM10 being a key regulator of DNA replication, where it was also shown to interact with ssDNA and multiple essential DNA replication proteins such as CDC45, MCM2-7, DNA polymerase, PCNA and GINS 7-11, required for accurate replication and proliferation of the cells, might also be involved in BC progression via impairing DNA replication or cellular proliferation. To test our hypothesis, we overexpressed MCM10 in immortal non-tumorigenic MCF10A mammary cells, expressing low MCM10 levels (Fig. 2 A-B). MCM10 OE in MCF10A (MCF10A-OE) cells significantly increased the cell proliferation compared to the controls (MCF10A-Control). A significant difference in proliferation was observed at times 30, 60, 80 hours (Fig. 2C). We found that this increased proliferation was mediated by increased phosphorylation of AKT in MCM10 OE cells (Fig. 2K).

To find out whether increased proliferation during replication could initiate cell death signaling, we assessed the cell death by measuring apoptosis. Increased proliferation didn't show any difference in apoptotic cells (Fig. 2D-E), however, we noticed a relative increase in cell count in MCM10 overexpression cells compared to control post stable selection of the cells (Supplementary Fig. S5). We further decided to analyze cell cycle changes in MCF10A-OE cells and performed cell cycle analysis. Cell cycle analysis showed that overexpression of MCM10 shortened the cell cycle specifically by decreasing the time duration spent in the S-phase of the cell cycle (Fig. 2F-I). We also validated our cell cycle analysis results by observing CDK2, a S-phase cell cycle marker. The CDK2 (Cyclin-dependent kinase 2) has been shown to generate hyper-phosphorylated retinoblastoma protein RB which stimulates the activity of DNA polymerase alpha and acts as a cell-cycle promoter 23. We studied protein expression of CDK2 at different times in control and MCF10A-OE cells (Fig. 2J) and found high expression of CDK2 in MCF10A-OE post synchronization. Together, these results indicated that MCM10 OE can increase cell proliferation which could be mediated by a shortened cell cycle particularly the S-phase in MCF10A-OE cells and activated AKT signaling.

### MCM10 overexpression leads to the accumulation of ssDNA binding protein RPA in MCF 10A cells

**Fig 3:**
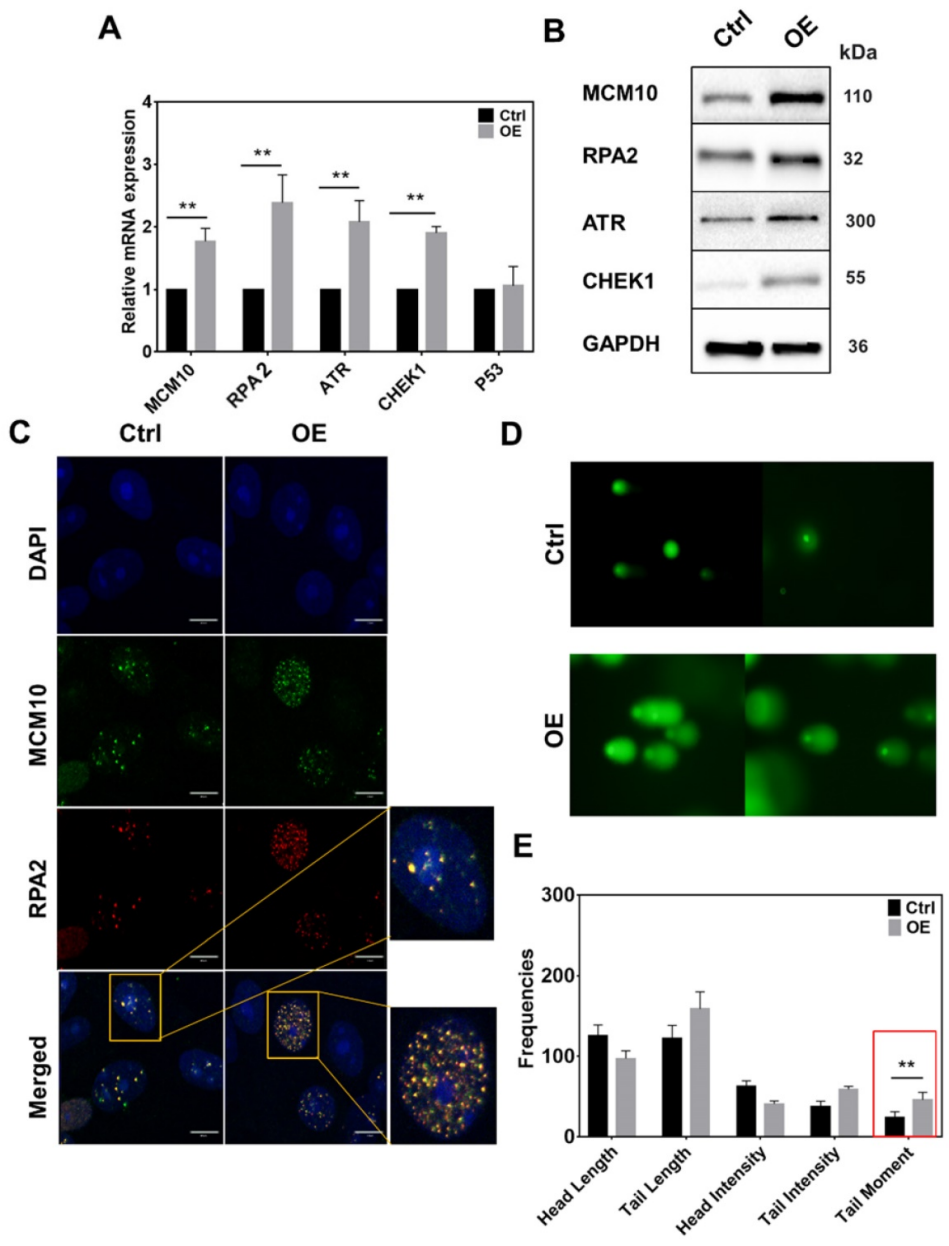
High expression of MCM10 induces an increase in ssDNA accumulation and ssDNA binding protein RPA2 in MCF 10A cells. A) mRNA quantification of RPA2, ATR and CHEK1. B) Western blotting showing expression of RPA2, ATR and CHEK1 proteins in MCF10A-control and MCF10A-OE. C) Immunofluorescence images of MCF10A cell nucleus stained for MCM10 and RPA2. Colocalization of MCM10 and RPA2 was observed at 100X magnification, Scale bar, 10um. D) Representative images of single cell gel electrophoresis in control and MCF10A-OE cells stained with SYBR green and observed under an epifluorescence microscope. E) Bar graph showing frequencies of length, intensities and tail moment in MCF10A-control and MCF10A-OE cells, analyzed by comet assay IV software. A significant difference was observed in Olive's tail moment in MCF10A-OE compared to control. The student t-test was used to analyze the difference between the two groups and the data are presented as mean values ± s.d. Value of p< 0.05 was considered significant (*), while p< 0.01 was considered markedly significant (**).

MCM10 plays an important role in the activation of RC helicase that unwind DNA during replication. Increased MCM10 expression could accelerate the process of DNA unwinding which can lead to the accumulation of ssDNA during replication and alter the replication rate. To analyze increased ssDNA, we monitored a subunit of ssDNA binding protein Replication protein A (RPA2) in MCF10A-OE cells. In line with our hypothesis, RPA2 had a significant increase in MCM10 OE cells specifically at 2 and 4 hours post synchronization (Fig. 3, Supplementary Fig. S4A-C). The same increase was also observed at protein level at 4 hours (Fig. 3B). Also, immunofluorescence staining (IF) of MCF10A-OE cells showed an increased overlap of protein MCM10 and RPA2 within nucleus (Fig. 3C). We also analyzed downstream targets of ssDNA such as ataxia telangiectasia and Rad3 related (ATR) and Checkpoint kinase 1 (CHEK1) in MCF10A OE cells. We could find a significant increase in total ATR and total CHEK1 (Fig. 3A-B), however, the phospho form of ATR and CHEK1 was not evident (Supplementary Fig. S6). To confirm our approach, we also used UV treated MDA-231 as a positive control (Supplementary Fig. S6). Overexpression of MCM10 may have increased the number of origin firing, which lead to an increase in single stand binding protein RPA2 in MCM10 overexpression cells. This may be the reason that we have observed relatively shorted S-phase of the cell cycle. To further validate if RPA2 is truly elevated due to accumulated ssDNA, we performed comet assay. Comet assay showed a significant increase in tail moment indicating the presence of ssDNA in MCF10A-OE cells (Fig. 3D-E). Persistence low level of replication stress by constant overexpression of MCM10 via ssDNA could be a possible source of total ATR/CHEK1 increase in MCM10 OE cells. A partial increase in ATR/CHEK1 in MCM10 OE cells indicated an imbalance in feedback termination of stressed cells.

### MCM10 overexpression in NIH3T3 cell line induces early onset of cell senescence

**Fig 4:**
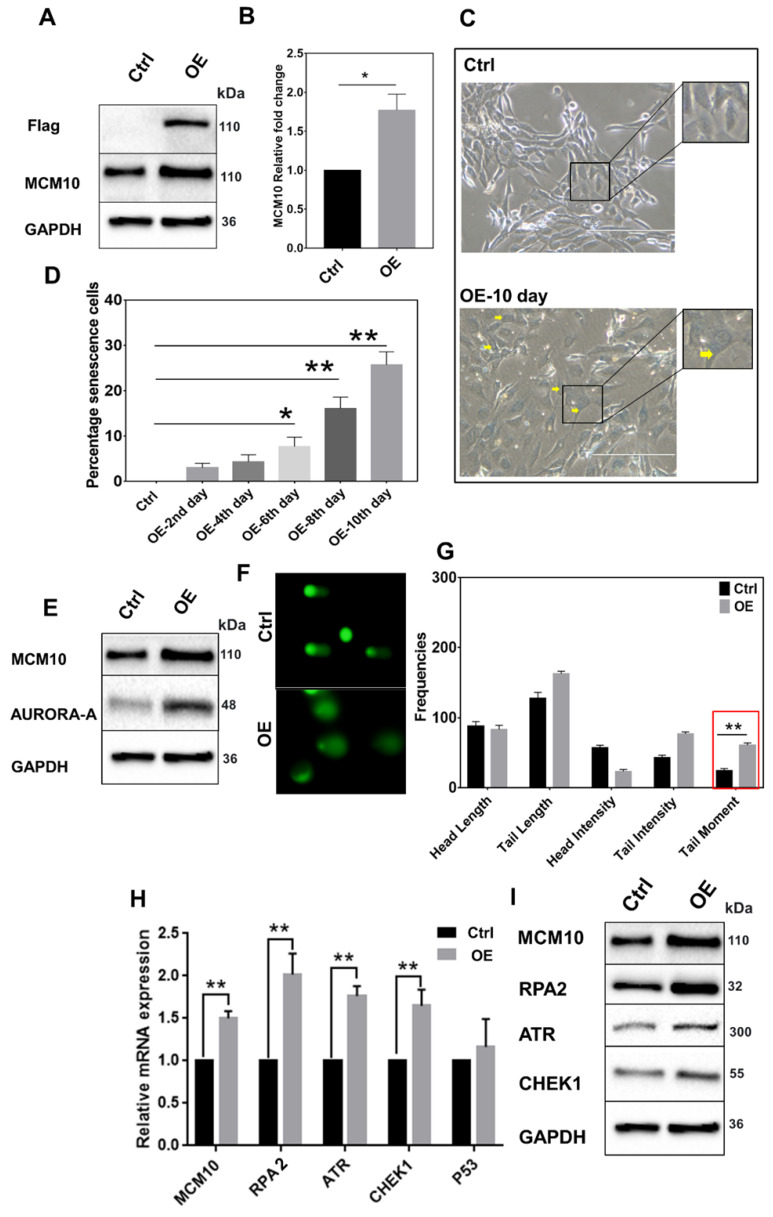
High expression of MCM10 in NIH3T3 cells. A) Protein and, B) mRNA expression of MCM10 in NIH3T3-control and NIH3T3-OE. C) Phase-contrast images of β-galactosidase staining for cell senescence (arrow), taken at 20X. D) Relative percentile of senescence cells at different time points post stable cell selection**.** A significant increase in the percentile of senescence cells was observed on 6^th^, 8^th^ and 10^th^ days after stable cell selection. E) Expression of DNA damage sensing protein Aurora-A in NIH3T3-control and NIH3T3-OE, showing increased protein expression of aurora-A in NIH3T3-OE cell compared to control. F) Representative images of single cell gel electrophoresis in control and NIH3T3-OE cells stained with SYBR green and observed under an epifluorescence microscope. G) Bar graph showing frequencies of length, intensities and tail moment in control and NIH3T3-OE cells, analyzed by comet assay IV software. A significant difference was also observed in Olive's tail moment in NIH3T3-OE cells compared to control. H) mRNA quantification of RPA2, ATR and CHEK1 in NIH3T3-control and NIH3T3-OE. I) Western blotting showing expression of RPA2, ATR and CHEK1 proteins in NIH3T3-control and NIH3T3-OE. RPA2, ATR and CHEK1 expression was observed significantly high in MCM10-OE cell line compared to control. The student t-test was used to analyze the difference between the two groups and data are presented as mean values ± s.d. Value of p< 0.05 was considered significant (*), while p< 0.01 was considered markedly significant (**).

The above observation indicated a possible occurrence of genomic instability in MCM10 OE cells. To analyze this possibility, we studied cell senescence, a prerequisite for genomic instability in MCM10 OE in another normal Mouse embryonic fibroblasts cell line (NIH3T3). NIH3T3 cells lack telomerase activity and are good models for analyzing cell senescence induced by replication stress and genomic instability. We overexpressed MCM10 in NIH3T3 by retroviral transfection (Fig. 4A-B). In line with our earlier results, cells with higher expression of MCM10 showed an increase in ssDNA binding protein RPA2 and ATR (Fig. 4H-I). Further, β-galactosidase staining revealed an increase in positive β-galactosidase stained cells with time, indicating the presence of cell senescence activity (Fig. 4C-D, Supplementary Fig. S3). As an indirect evidence for cell senescence, we also analyzed DNA damage checkpoint related protein aurora-A. Increase in MCM10 or DNA damage during G1/S phase induced an increase in aurora-A protein (Fig. 4E). We also performed single cell gel electrophoresis in NIH3T3-OE and control cells to observe DNA damage and DNA fragmentations. Results of single cell gel electrophoresis in NIH3T3-OE showed a significant increase in DNA tail moment (Fig. 4F-G). Altogether results in NIH3T3 cells indicated positive senescence, accumulated ssDNA followed by relatively high expression of ssDNA binding protein RPA2 with MCM10 overexpression, which is pivotal in understanding MCM10 and genomic instability relationship.

### The association of MCM10, RPA, ATR and CHEK1 in Breast Cancer patient cohorts

**Fig 5:**
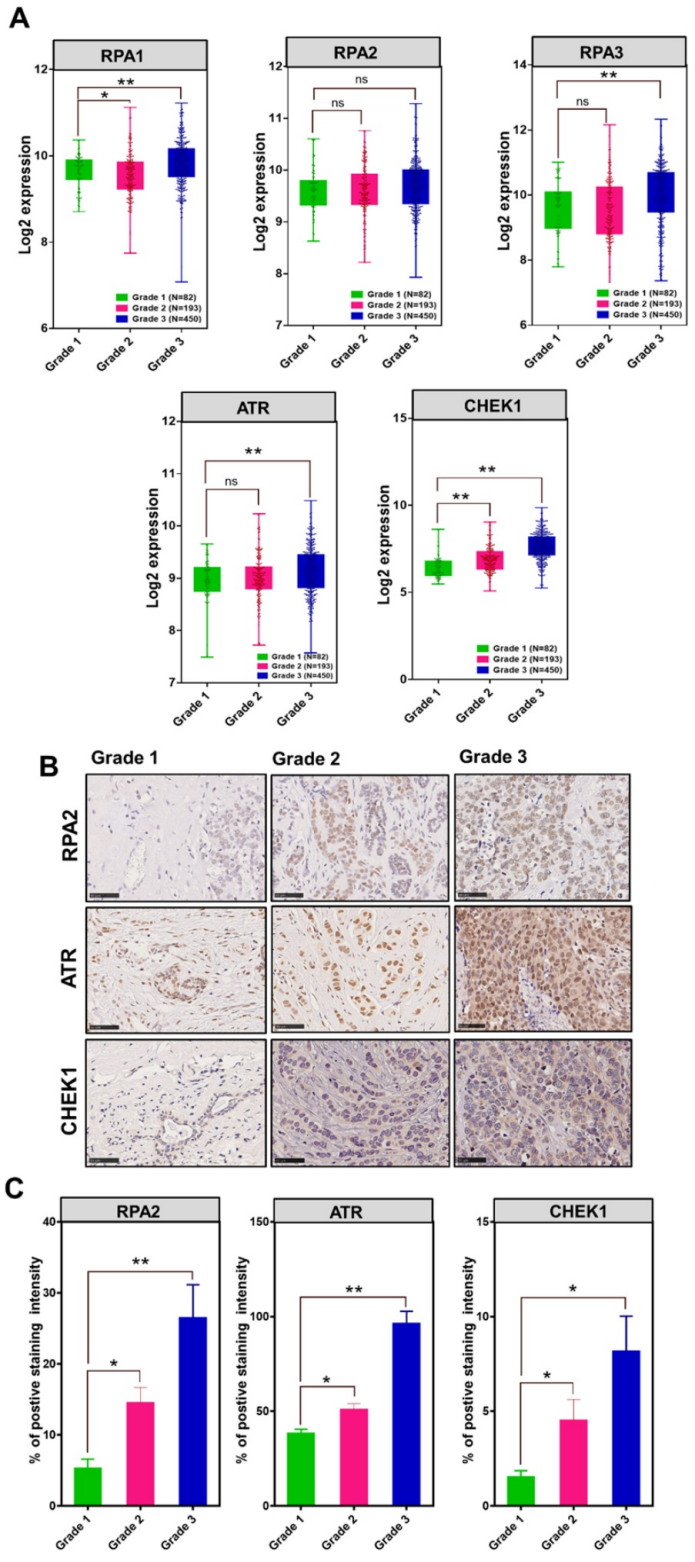
The association of MCM10, RPA, ATR and CHEK1 in combined BC patient cohorts and their survival status analysis along with their validation by IHC in clinical patient samples. A) Box plots showing log2 expression of different subunits of RPA, ATR and CHEK1 expression in combined BC patient's cohorts with different tumor grades. An increased expression of RPA1&3, ATR and CHEK1 with a higher tumor grade was observed. High expression of cellular RPA1&3, ATR, and CHEK1 proteins were observed in clinical patient samples with a higher tumor grade, particularly in Grade 3 BC compare to grade 1 BC. B-C) Immunohistochemistry staining of RPA2, ATR and CHEK1 in Grade 1, Grade 2 and Grade 3 BC tissue samples, images were taken at 40X. IHC staining also showed higher expressions of cellular RPA2, ATR, and CHEK1 proteins in clinical patient samples with a higher degree of aggressiveness. The significance of the difference between two groups was analyzed by variance analysis (Mann Whitney test: Patients datasets acquired from GENT2 database, Student t-test: Experimental data), and results are expressed as the mean value with standard deviation. A value of p< 0.05 was considered significant (*), while p< 0.01 was considered markedly significant (**).

To investigate the association between MCM10 and DDR genes in BC patients having different degree of malignancy, we retrieved DNA damage response genes expressions data from the same datasets that we utilized for exploring the MCM10 expression pattern using GENT2 database. Initially, we looked at expression patterns of these genes in grade 1-3 BC specimens. Fascinatingly, we observed quite similar trends in expression levels of different subunits of RPA1-3, ATR and CHEK1 in tumors with a higher degree of aggressiveness as we observed in MCM10 and a significant difference in the expressions of RPA1, RPA3, ATR and CHEK1 genes were seen in grade 1 and grade 3, (Fig. 5A). To validate the *in-silico* association of MCM10 expression and DDR activation, and to see the cellular protein levels of DDR proteins in the relevance of MCM10, we performed IHC staining in clinical BC patients specimens with grade 1, grade 2 and grade 3 of BC malignancy. Interestingly, RPA2, ATR and CHEK1 expressions in these grades tumors were overlapping with MCM10 expression (Fig. 5B-C). Cumulatively these results provided direct evidence that MCM10 expression can up-regulate DDR during early stages of cancer and involve in BC progression.

### MCM10 overexpression promotes tumorigenic properties in MCF10A cells

**Fig 6:**
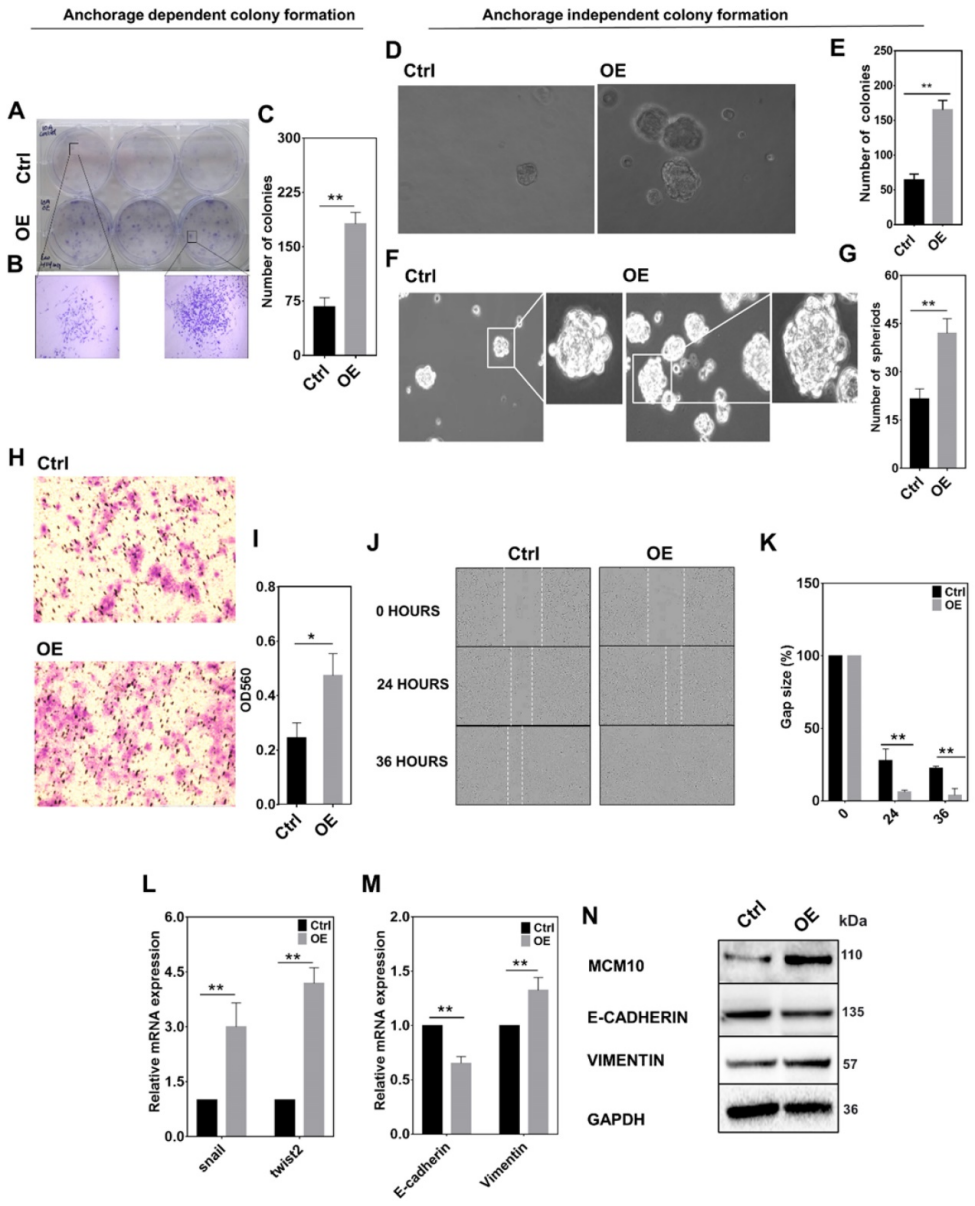
High expression of MCM10 in MCF10A promotes migration and anchorage independent growth. A) Images of 2D colony formation (anchorage dependent growth) in MCF10A-control and MCF10A-OE. B) Zoomed images of morphological changes observed in 2D colony formation assay. C) Bar graph showing a significantly increased number of colonies in MCF10A-OE compared to control. D) Images of soft agar colony formation (anchorage independent growth) assay taken at 20X. E) Bar graph of agar colony formation assay, showing significantly increased number of colonies in MCF10A-OE compared to control. F) Images of spheroid formation assay showing the morphological changes in MCF10A-OE and control group observed at 20X magnification. G) Bar graph showing a significantly increased number of spheroids in MCF10A-OE compared to the control (Anchorage independent colony formation). H) Images of cell migration determined by Transwell migration assay of MCF10A-OE and control taken at 4X magnification. I) Bar graph showing a significant increase in cell migration in MCF10A-OE compared to control. J) Cell migration determined by scratch wound Assay analyzed using Incucyte ZOOM analysis software at 10X magnification. K) Bar graphs showing a significant increase in cell migration in MCF10A-OE cells compared to the control at 24 and 36 hours. L) Significant increase in mRNA levels of Snail and Twist2 in MCF10A-OE cells compared to the control. M) mRNA expression of E-Cadherin and Vimentin in control and MCF10A-OE cells. N) Western blotting showing protein expression of E-cadherin and Vimentin in control and MCF10A-OE cells. A significantly decreased expression of E-cadherin and increased expression of Vimentin was observed on mRNA and protein levels. The Student t-test was used to analyze the difference between the two groups and data are presented as mean values ± s.d. Value of p< 0.05 was considered significant (*), while p< 0.01 was considered markedly significant (**).

To further confirm the involvement of MCM10 in BC progression, we studied MCM10 expression's influence on cellular properties such as proliferation, migration and anchorage dependent and independent growth. We analyzed MCM10 OE in MCF10A cells using a panel of relevant assays. Using 2D Clonogenic assay we analyzed anchorage dependent growth and, using soft agar and spheroid formation assays we observed anchorage independent growth in MCM10 OE cells. We found a significant increase in number of colonies in both anchorage dependent (Fig. 6A-C) and anchorage independent colony formation in MCM10 OE cells compared to controls (Fig. 6D-E). MCM10 OE cells also showed a significant increase in the number of spheroids compared to controls (Fig. 6F-G) observed by spheroid formation assay. We further considered analyzing the migration of cells under serum deprivation using Transwell assay and also migration after cell confluency by scratch wound assay. Both assays did show significant differences at different time points (Fig. 6H-K). These observations indicate a possible change in cellular characters of MCF10A cells after overexpressing MCM10. We hypothesized that these characters could have originated from increased proliferation. To analyze this, we quantified Snail, Twist (two frequently reactivated transcription factors in various cancers) and E cadherin and Vimentin (markers of cellular properties) in these cells (Fig. 6L-N). ) A significant increase in mRNA levels of Snail and Twist2 in MCF10A-OE cells compared to the control was observed by qPCR (Fig. 6L). mRNA expression of E-Cadherin and Vimentin in control and MCF10A-OE cells was also seen significantly decreased and increased respectively (Fig. 6M). Similar changes in proteins levels of these proteins in control and MCF10A-OE cells were also detected (Fig. 6N). Taken together, these results indicated that high expression of MCM10 could promote tumorigenic properties either directly or indirectly by impairing the relevant pathways.

## Discussion

Cell proliferation is one of the most critical characteristics of development and goes misregulated in many cancers. A crucial part of cell proliferation is the replication of DNA that occurs during S phase of the cell cycle and is recognized as a vital biological activity for preserving the stability and integrity of the genome. Recent study into the regulation of replication timing has demonstrated that MCM10 mutations are associated to significant replication timing variations 3. In addition, MCM10 has also been shown to interact with ssDNA and various critical DNA replication proteins including CDC45, MCM2-7, DNA polymerase, PCNA, and GINS 7-11, all of which are required for accurate cell replication and proliferation. This implies that aberrant expression of MCM10 may contribute to impaired replication and abnormal proliferation, which might lead to genomic instability and cancer development or progression 24. Downregulation of MCM10 during the early S phase hinders the cell cycle progression 25 and increased S and Early G2 cells 26. Unfortunately, the role of increased expression of MCM10 during pre-replication is least understood, despite overexpression of MCM10 expression has been reported in various cancers 12. In this study, we hypothesized that expression levels of MCM10 are associated with the degree of aggressiveness in clinical breast cancer patients, and increased MCM10 could enhance the cancer-like characteristics in normal cells. To validate our hypothesis and discover the underlying mechanism, we observed expression levels of MCM10 in breast cancer patients having a different degree of aggressiveness. We were also the first to establish MCM10 overexpression model to study the influence of MCM10 on immortal non-tumorigenic MCF10A and embryonic fibroblast cells NIH3T3 cells.

Many previous studies have reported that MCM10 expression is high in a number of cancers such as lung cancer, cervical cancer, urothelial carcinoma, gastric cancer, esophageal cancer and BC 27-31. We analyzed the expression patterns of MCM10 in relation to BC in patient cohorts by acquiring microarray gene expression data from the GENT2 database, in *ex-vivo* patients specimens having a different degree of BC malignancy and, in less aggressive and highly aggressive breast cancer cell lines. MCM10 expression was found consistently and significantly higher in tumors with a higher malignancy, a higher tumor grade, and highly aggressive cancer cell lines. Also, we observed that MCM10 expression levels were potentially able to discriminate accurately among different grades BC. Using MCM10 OE model, we observed that MCM10 OE in MCF10A increased cell proliferation possibly by increasing the binding of MCM10 to multiple MCM2-7 complex during the S phase and G2 phase. Stable expression of MCM10 at different time points was confirmed by Western blot and qPCR (data not shown). MCM10 OE shortened the cell cycle particularly the S phase during the cell cycle followed by an increased population of G2 cells in MCM10 OE cells at 12 hours. Shortened S phase indicates the possibility of a high DNA replication rate. In cancer cells, knocking down MCM10 has been observed to increase S and Early G2 cells due to incomplete replication initiation 26. In accordance, high expression of CDK2 (an S phase maker of cell cycle) in MCF10A-OE cells post synchronization was also observed. The CDK2 has been shown to generate hyper-phosphorylated retinoblastoma protein which stimulates the activity of DNA polymerase alpha and acts as a cell-cycle promoter 23. It was also previously reported that elevated expression of MCM3 (another member of MCMs) promoted G1/S cell cycle progression, proliferation, invasion and migration in colorectal cancer 32. Similar results were observed by Wang, Liuxin, et al. where overexpression of PCNA, a known interaction of MCM10 in budding yeast promoted cell proliferation, clonal formation, and tumorigenesis in lung cancer cells and inhibited cell apoptosis 33. Another DNA replication initiation factor CIZ1 overexpression has been shown to promote the growth and migration of hepatocellular carcinoma 34. MCM10 overexpression in MCF10A did not induce any cell death-related responses, which indicated a possible low-level persistent stress. MCM10 binding to MCM2-7 complex at multiple origin sites can cause accumulation of ssDNA and increase DNA replication rate. Although multiple origin firing was not confirmed in the current study, we used a measure of RPA2, a single strand DNA binding protein as an indirect indicator of multiple firing and ssDNA accumulation. MCM10 OE was able to increase RPA2 compared to controls in breast cancer patient biopsy and in MCF10A OE cells, which further induced high expression of downstream proteins ATR and CHEK1, supporting the possibility of increased ssDNA in MCM10 OE cells. However, *in-silico* RPA1 and RPA3 were signicantly higher in grade 3 compared to grade 1. We were unable to detect the phospho forms of these proteins in MCM10 OE cells. One possible reason could be that the activation of these proteins upon DNA damage may have initiated ssDNA repair mechanisms leading to ssDNA repair, halt cell cycle progression or apoptosis induction. We confirmed this by looking at the expression levels of p53 and XRCC1, two key genes involved in single strand DNA repair post ATR and CHEK1 activation, and observed no difference in their expression (Supplementary Fig. S7). This also indicates the persistence low level of DNA damage post MCM10 overexpression. The presence of ssDNA was also confirmed by comet assay, which showed a significant increase in the nuclear tail moment. Together, these data indicated accumulation of ssDNA and persistence low level of replication stress in MCM10 OE cells. MCM10 expression is highly regulated within the cells. MCM10 OE, however, didn't affect p53 expression or apoptosis. Thus, the effect of MCM10 overexpression on cellular senescence was evaluated by measuring cellular senescence in NIH3T3 cells. NIH3T3 cell line is an ideal model for such study as it lacks telomerase activity and is generally used for measuring DNA instability by β-galactosidase staining 35, 36. Overexpression of MCM10 in NIH3T3 cells induced an increase in β-galactosidase stained positive cells. The gradual increase in staining was observed at different time points specifically in MCM10 OE cells. Inherent β-galactosidase staining of NIH3T3 has been shown to exhibit biochemical and morphological changes related to replicative senescence and reported by many studies. DNA damage was also validated using Aurora-A protein, A DNA damage sensing protein downstream to RPA/ATR signaling 37, 38. We observed an increase in Aurora-A, which indicated DNA damage in NIH3T3-OE cells. This observation suggests the positive influence of MCM10 OE on genomic instability. Although many studies have shown increased MCM10 expression in highly proliferating tumors, direct evidence linking MCM10 and cancer phenotype is yet lacking 39. Earlier studies have reported PI3K/AKT signaling pathway, as well‐characterized and the most important signaling pathways activated in response to DNA damage 40. PI3K/AKT signaling plays a key role in cell physiology as a critical regulator of cell survival, proliferation and metabolism 41. Recently, PI3K/AKT signaling has also been shown to regulate cell migration and tumorigenesis 40, 42, 43. Overexpression of MCM10 in normal epithelial cells leads to an increase in Phospho-AKT (ser473) and Phospho-Gsk-3β. This upregulation indicates activation of AKT signaling pathway in response to high expression of MCM10. Moreover, a significant increase in mRNA levels of snail (Zinc finger protein SNAI1) and twist2 (twist family bHLH transcription factor 2), a downstream target of PI3K/AKT signaling was also observed. Phenotypically, this upregulation could promote a change in cellular characters leading to the transformation from epithelial to mesenchymal cell 44. Studies show that the activation of DNA stress mediates phosphorylation of AKT, GSK3β and Snail to promote tumorigenesis 45, 46. Upregulation of Snail and Twist2 can also be indirectly regulated by ATR through activation of ZEB1 (Zinc Finger E-Box Binding Homeobox 1) which then can trigger tumorigenesis and relative markers 47. Snail can suppress E-cadherin expression, a transmembrane glycoprotein that connects epithelial cells together at adherent junctions to prevent mesenchymal transformation 48. Twist2 is also shown to be involved in p53/RB signaling inhibition and is considered a potential mediator of mesenchymal transformation 49. These phenomena also explain the unaltered expression of tumor suppressor gene p53 upon activation of DDR in MCM10 OE cells. Mesenchymal transformation is characterized by the combined loss of epithelial cell junction proteins such as E-cadherin and the gain of mesenchymal markers such as Vimentin cellular properties of MCF10A by growing them in a culture closely mimicking the *in-vivo* conditions. Relatively high migration, anchorage independent growth and spheroid formation were seen in MCF10A-OE group compared with the control. The ability of cells to exhibit anchorage-independent cell growth, migration and colony formation has been shown to promote tumor cell aggressiveness *in-vivo*, and also utilized as a marker for *in-vitro* transformation 50, 51 We also showed the relationship among MCM10, DDR genes and tumor progression by both *in-silico* analysis in patient cohorts and *ex-vivo* patient sample analysis. Moreover, patients whose BC expressed a low level of MCM10, had significantly longer survival than those patients whose tumors expressed a higher level of MCM10. Altogether these observations indicated the role of MCM10 in inducing DNA replication catastrophe and dictating the aggressiveness of breast cancer.

## Conclusion

Our results demonstrated that MCM10 expression levels are closely linked with the degree of malignancy in BC patients. Mechanically, increased expression of MCM10 plays a vital role in inducing proliferation and accumulating ssDNA without activating the DDR, resulting to replication catastrophe and cancer development. Thus, MCM10 could act as both, a potential clinical marker to detect the active degree of BC malignancies and a capable therapeutic target for future cancer therapy.

## Supplementary Material

Supplementary figures and tables.

## Abbreviations

RC: Pre-replication complex
GINS: Go-ichi-ni-san
CMG: Cdc45, Mcm2-7, GINS
MCM10: Minichromosome maintenance protein 10
ssDNA: Single strand DNA
DDR: DNA damage response
BC: Breast cancer
ROC: Receiver operating characteristic
GENT2: Gene Expression database of Normal and Tumor tissues 2
TNBC: Triple negative Breast cancer
CDK2: Cyclin-dependent kinase 2
RB: Retinoblastoma protein
RPA: Replication protein A
ATR: Ataxia telangiectasia and Rad3 related
CHEK1: Checkpoint kinase 1
AKT: Phosphatidylinositol-3-Kinase and Protein Kinase B
SNAIL: Zinc finger protein SNAI1
TWIST2: Twist family bHLH transcription factor 2
ZEB1: Zinc Finger E-Box Binding Homeobox 1
