# Supplementary material for: Over-Activation of Minichromosome Maintenance Protein 10 Promotes Genomic Instability in Early Stages of Breast Cancer
Source: Int J Biol Sci. 2022 May 29;18(9):3827–44. doi: 10.7150/ijbs.69344 (PMC9254470; doi:10.7150/ijbs.69344)
Supplement: Supplementary file 1 — Supplementary figures and tables. [file ijbsv18p3827s1.pdf]

Supplementary files for the manuscript entitled

"Over-Activation Of Minichromosome Maintenance Protein 10 Promotes Genomic Instability In Early Stages Of Breast Cancer"

Muhammad Jameel Mughal[1^], Kin Iong Chan[2^], Ravikiran Mahadevappa[1], Sin Wa Wong[1], Kit Cheng Wai[2], Hang Fai Kwok[1*]

[1]*Cancer Centre, Faculty of Health Sciences, University of Macau, Avenida de Universidade, Taipa, Macau SAR; MOE Frontiers Science Center for Precision Oncology, University of Macau, Avenida de Universidade, Taipa, Macau SAR*

[2]*Centre for Precision Medicine Research and Training, Faculty of Health Sciences, University of Macau, Avenida de Universidade, Taipa, Macau SAR; Department of Pathology, Kiang Wu Hospital, Macau SAR*

^Co-first author

***Corresponding Author: Hang Fai Kwok**

Mailing address:

Faculty of Health Sciences (E12), University of Macau, Avenida de Universidade, Macau SAR

Tel: +853 8822 4991

Email: hfkwok@um.edu.mo

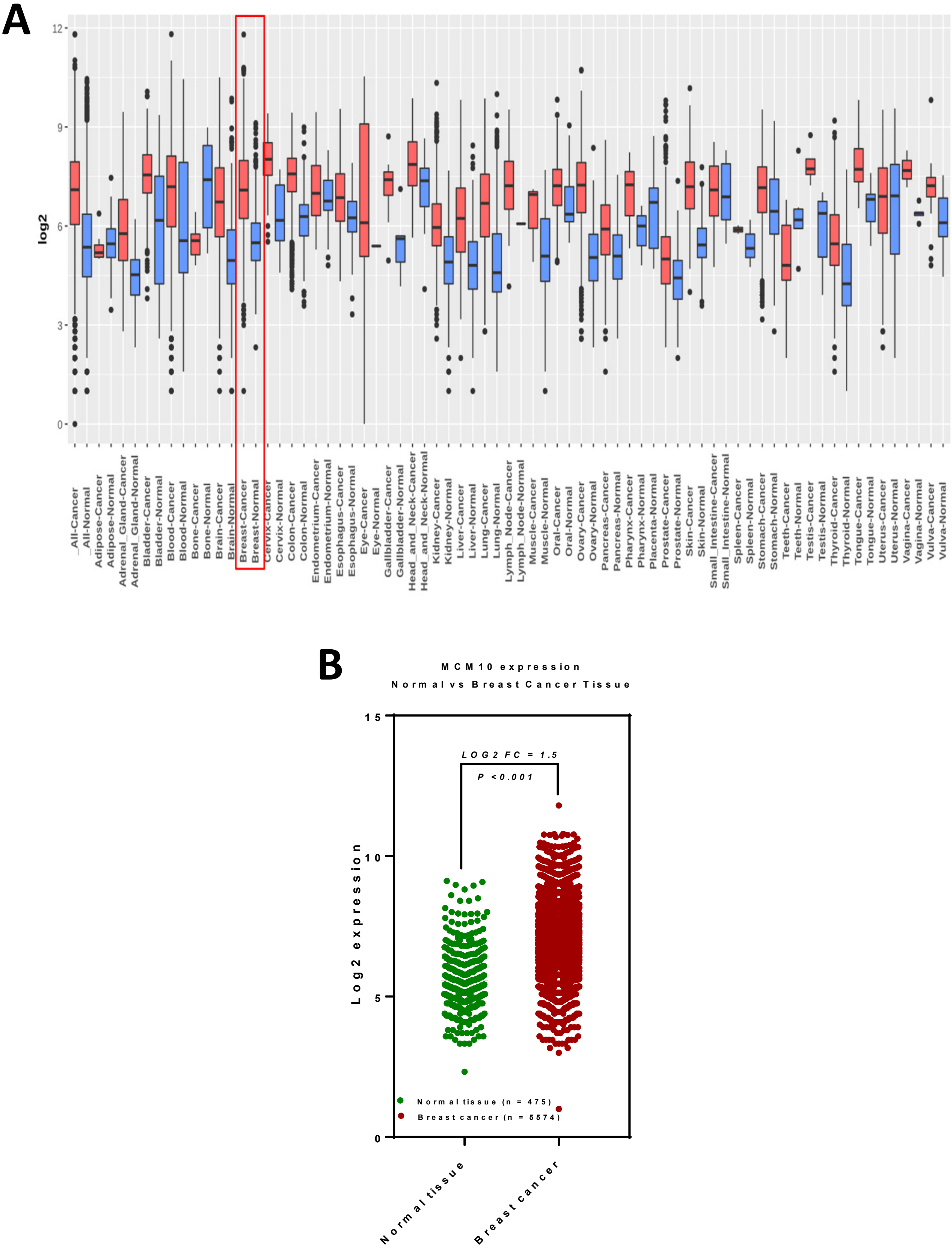

Fig. S1: MCM10 expression across multiple cancers vs their normal tissues analyzed using GENT2 (Gene Expression database of Normal and Tumor tissues 2) database that contains gene expression data of 68,000 clinical samples. A, MCM10 expression in multiple cancers compare to normal tissue compared to normal tissues. B, MCM10 expression in breast cancer

compare to normal tissue manually analyzed by retrieving MCM10 expression data from GENT2 database. A log2 fold change of 1.52 and a *p* value <0.001 was observed in breast cancer tissues compare to normal breast tissues.

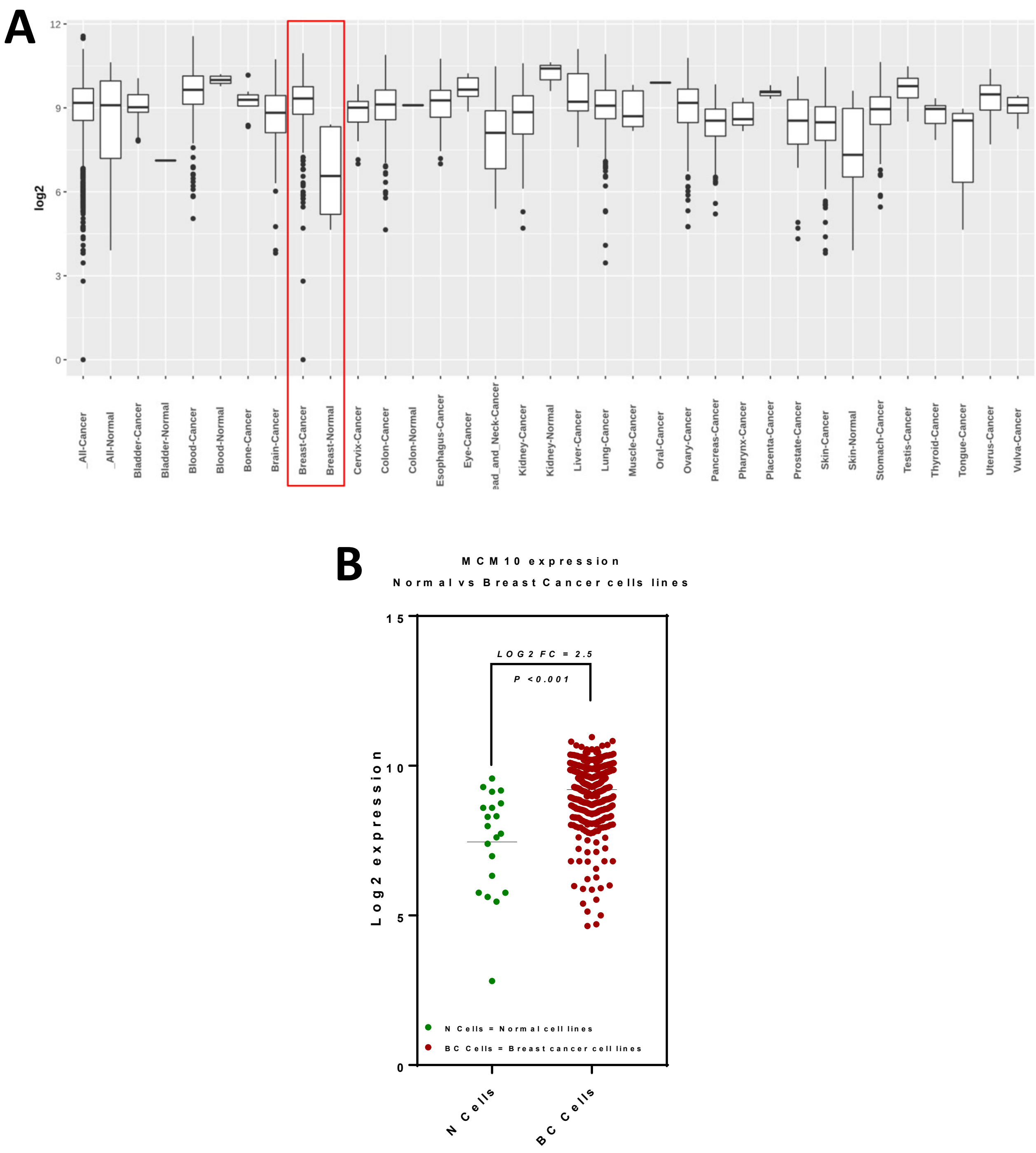

Fig. S2: MCM10 expression across multiple cancers cell lines vs their normal tissues cell lines analyzed using GENT2 (Gene Expression database of Normal and Tumor tissues 2) database. A, MCM10 expression in various cancers cell lines vs their normal tissues cell lines. B, MCM10 expression in breast cancer cell lines compared to normal tissue cell lines manually analyzed by retrieving MCM10 expression data from GENT2 database. A log2 fold

change of 2.5 and a *p* value <0.001 was observed in breast cancer cell lines compare to normal cell lines.

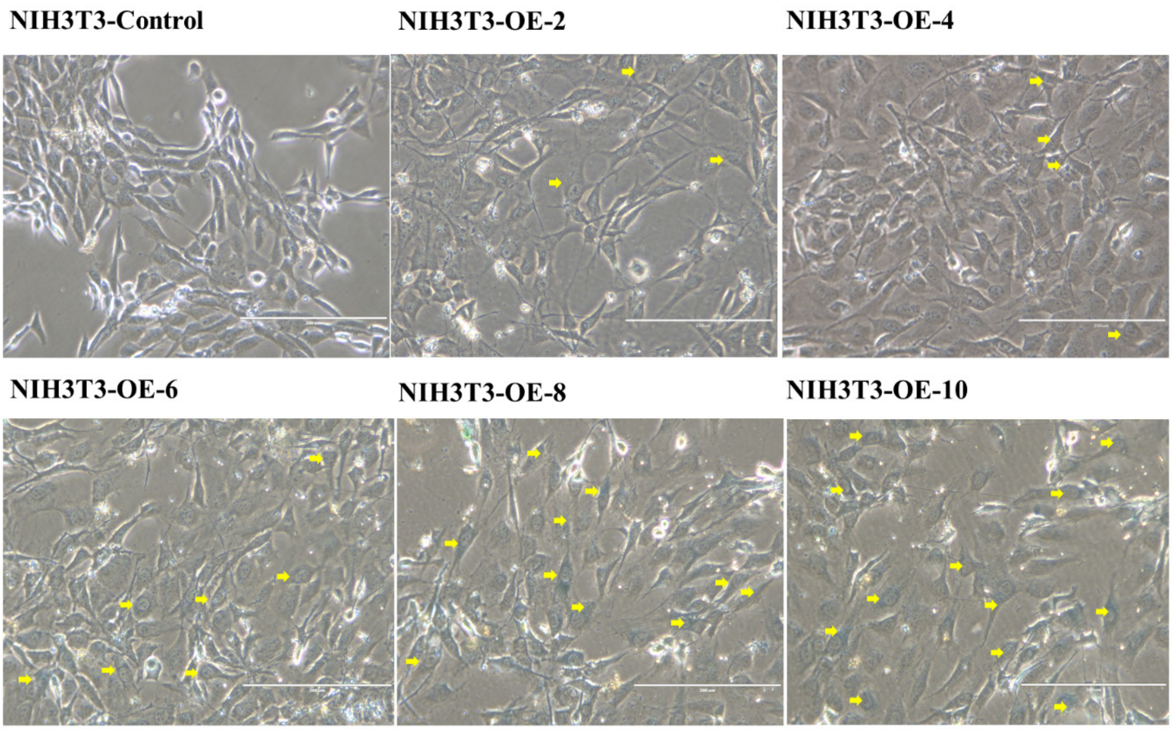

Fig. S3. Phase-contrast images of beta Galactosidase staining for cell senescence in NIH3T3 cell line at different time points (days) post stable cell selection**.**

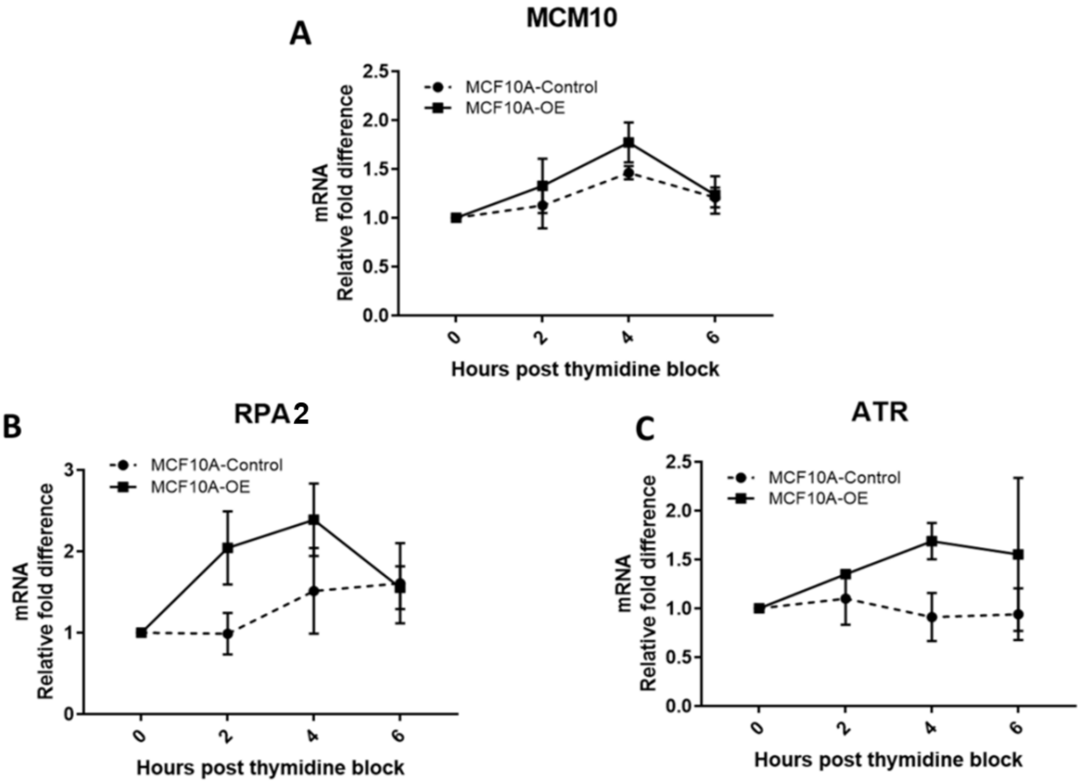

Fig. S4. Relative mRNA quantification of MCM10, RPA, ATR at different time point post thymidine block in MCF10A cells. A, mRNA quantification of MCM10 at 0, 2, 4 and 6 hours post thymidine block. B, mRNA quantification of RPA at 0, 2, 4 and 6 hours post thymidine block. C, mRNA quantification of ATR at 0, 2, 4 and 6 hours post thymidine block. The significance of difference between control and OE groups was analyzed by variance analysis, and results are expressed as the mean value with deviation. The analysis was performed using the independent sample t test of prism 7 software and a value of p< 0.05 was considered significant, while p< 0.01 was considered markedly significant.

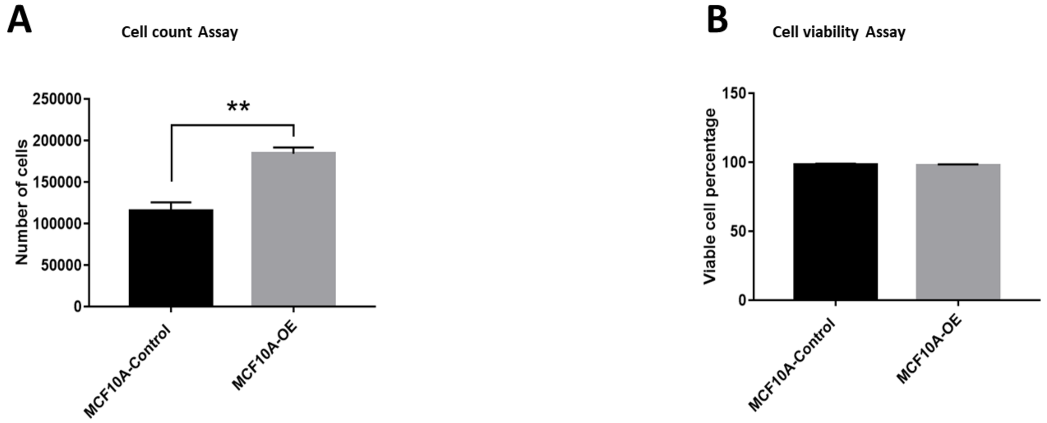

Fig. S5. Cell count and cell viable assays sowing difference in number of cells and viable cells in MCF10A control and OE cells. A, Results of cell count assay showing relatively significant cells in MCF10A-OE cells compare to MCF10A control after stable cell selection. B, Results of cell Viability assay showing no difference in cells viability in both MCF10A-OE and control groups. The significance of difference between control and OE groups was analyzed by variance analysis, and results are expressed as the mean value with deviation. The analysis was performed using the independent sample t test of prism 7 software and a value of p< 0.05 was considered significant, while p< 0.01 was considered markedly significant.

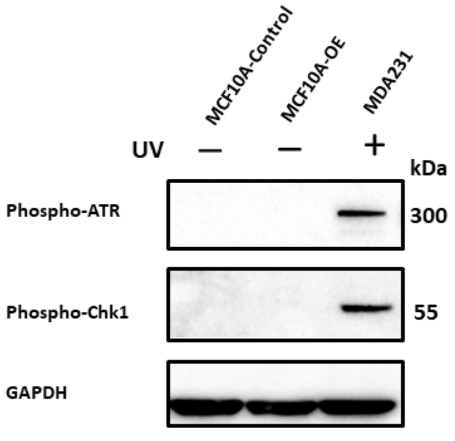

Fig. S6. Western blot analysis showing protein expression level of Phopho-ATR and CHEK1 proteins in MCF10A-Control, OE and UV-treated MDA231. Western blot analysis showed expression level of Phopho-ATR and CHEK1 proteins in UV-treated (50 mj, 30 min) MDA-231 cell lines, however, no expression level of these proteins in MCF10A control and MCF10A-OE, was observed.

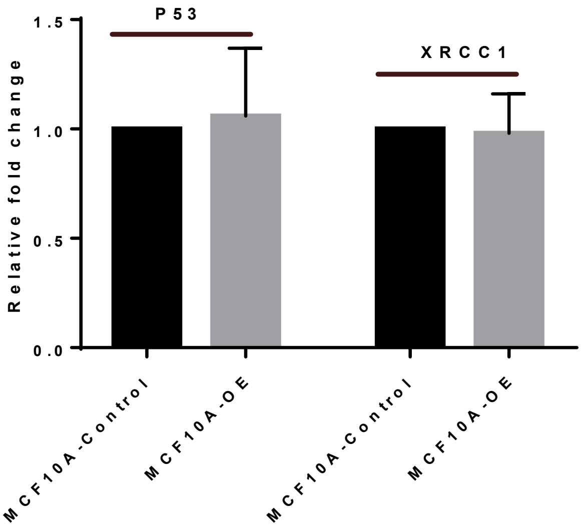

Fig. S7. Relative mRNA quantification of ssDNA repair genes P53 and XRCC1 in MCF10A cells. No difference between MCF10A-OE and Control for P53 and XRCC1 was observed.

Table S1. List of Primary and secondary antibodies used in this study

Primary antibodies	Supplier	Species	Dilution for WB	Dilution for IF	Dilution for IHC	Cat No.
MCM10	Bethyl laboratories, Montgomery, TX, USA	Rabbit	1:1000	1:500	1:250	A300-131A
VIMENTIN	Cell signaling technology	Rabbit	1:1000			5741
FLAG	Sigma Aldritch	Mouse	1:5000			F1804
E-CADHERIN	Cell signaling technology	Rabbit	1:1000			3195S
ATR	Santa cruz biotechnology	Mouse	1:400	1:100		sc-515173
ATR	LSBio	Rabbit			1:100	LS-C806721
RPA2	Cell signaling technology	Rat	1:1000	1:500		2208T
RPA2	Cell signaling technology	Rabbit			1:600	35869S
CHEK1	Santa cruz biotechnology	Mouse	1:400			sc-56291
CHEK1	Abcam	Rabbit			1:250	ab47574
AURORA A	Cell signaling technology	Rabbit	1:1000			14475
CDK2	Cell signaling technology	Rabbit	1:1000			2546T
GAPDH	Proteintech	Mouse	1:5000			60004-1-Ig
ANTI-GOAT ALEXA 555	Thermofisher	Mouse	1:500			A28180
ANTI-GOAT ALEXA 488	Thermofisher	Rabbit	1:250			A27034
ANTI-RABBIT HRP	Cell signaling technology	Rabbit	1:3000		1:500	7074
ANTI-MOUSE HRP	Cell signaling technology	Mouse	1:3000		1:500	7076
ANTI-RAT HRP	Cell signaling technology	Rat	1:3000		1:500	7077
PHOSPHO-GSK-3B (SER9)	Cell signaling technology	Rabbit	1:1000			9916
PHOSPHO-AKT (SER473)	Cell signaling technology	Rabbit	1:1000			9916
AKT (PAN)	Cell signaling technology	Rabbit	1:1000			9916

Table S2. List of primers used in this study

Primers		Sequence (5'->3')	Product length
MCM10	Forward primer	GTTCAGCTGTGAACGAAGAAGG	401
	Reverse primer	TCAGTTGACTGTGATGCGGG	
RPA2	Forward primer	ATTGCGGCCATCATGCAGAA	110
	Reverse primer	ATGAGCAGTCGATAACGCGG	
ATR	Forward primer	TTGTTCGCTGCCTGGATCAT	583
	Reverse primer	TCTTGCACAGCATCCCTGTT	
CHEK1	Forward primer	AGGGATCAGCTTTTCCCAGC	413
	Reverse primer	ATCATGTGGCAGGAAGCCAA	
Twist2	Forward primer	GGCGCAAGTGGAATTGGGAT	129
	Reverse primer	CGGGTCTTCTGTCCGATGTC	
Snail	Forward primer	TAGCGAGTGGTTCTTCTGCG	164
	Reverse primer	AGGGCTGCTGGAAGGTAAAC	
P53	Forward primer	GTTGGCTCTGACTGTACCACC	96
	Reverse primer	TGTGATGATGGTGAGGATGGG	
E-Cadherin	Forward primer	AGTGACTGATGCTGATGCCC	113
	Reverse primer	TGCCATCGTTGTTCACTGGA	
Vimentin	Forward primer	CTCTGGCACGTCTTGACCTT	817
	Reverse primer	TTGCGCTCCTGAAAAACTGC	
Gapdh	Forward primer	CTCTGCTCCTCCTGTTCGAC	408
	Reverse primer	GCGCCCAATACGACCAAATC	

Table S3. Gene expression of MCM10, RPA, ATR and CHEK1 in breast cancer patient's samples having different degree of BC malignancy based on tumor grades

Dataset_ID	Sample_ID	TS	N	ST	MCM10	RPA1	RPA2	RPA3	ATR	CHK1
GSE16446	GSM411345	BC	1	1	6.15	9.02	9.47	11.01	8.82	7.91
GSE16446	GSM411385	BC	2	1	6.46	8.71	9.37	10.85	9.07	6.88
GSE17907	GSM447238	BC	3	1	7.64	9.16	9.50	8.04	9.31	7.07
GSE17907	GSM447240	BC	4	1	8.60	10.00	9.94	8.03	8.81	7.33
GSE17907	GSM447244	BC	5	1	5.52	10.24	10.08	9.94	8.69	5.88
GSE20713	GSM519736	BC	6	1	4.52	8.90	10.08	9.80	9.16	5.64
GSE20713	GSM519762	BC	7	1	4.95	9.76	9.45	10.55	9.20	6.64
GSE20713	GSM519772	BC	8	1	6.04	9.63	9.58	10.68	7.73	6.17
GSE20713	GSM519788	BC	9	1	5.13	9.88	9.74	9.86	9.28	5.78
GSE20713	GSM519789	BC	10	1	6.09	9.96	9.95	9.93	8.75	6.11
GSE20713	GSM519790	BC	11	1	6.15	9.26	9.30	10.24	8.89	6.95
GSE20713	GSM519791	BC	12	1	5.81	10.18	8.63	9.77	9.10	6.21
GSE20713	GSM519795	BC	13	1	5.25	10.16	9.67	10.02	8.97	6.09
GSE20713	GSM519798	BC	14	1	5.58	9.85	9.44	10.74	9.24	6.04
GSE20713	GSM519804	BC	15	1	6.19	9.58	9.13	10.70	9.21	6.52
GSE20713	GSM519807	BC	16	1	5.04	9.89	9.39	10.52	9.13	6.15
GSE20713	GSM519809	BC	17	1	3.81	9.48	9.61	9.92	8.79	5.49
GSE20713	GSM519810	BC	18	1	5.32	9.14	10.26	10.55	9.07	6.73
GSE21653	GSM540341	BC	19	1	6.89	9.58	9.76	10.75	8.54	6.66
GSE21653	GSM540315	BC	20	1	5.52	9.94	10.59	9.94	8.69	5.88
GSE21653	GSM540321	BC	21	1	5.13	9.01	9.99	10.53	8.66	6.11
GSE21653	GSM540240	BC	22	1	5.67	9.83	10.29	9.64	8.95	6.00
GSE21653	GSM540197	BC	23	1	6.25	9.23	9.27	9.37	9.18	6.93
GSE21653	GSM540212	BC	24	1	5.67	9.77	9.62	10.09	8.89	5.93
GSE21653	GSM540198	BC	25	1	7.08	9.88	9.68	10.10	9.03	7.67
GSE21653	GSM540222	BC	26	1	6.32	9.97	9.46	10.82	8.74	6.38
GSE21653	GSM540337	BC	27	1	4.86	9.89	9.10	9.69	9.22	6.52
GSE21653	GSM540192	BC	28	1	6.61	9.69	9.32	8.91	8.99	5.83
GSE21653	GSM540318	BC	29	1	5.49	10.35	10.10	10.53	7.52	7.18
GSE21653	GSM540317	BC	30	1	5.00	9.95	10.57	9.72	8.74	6.09
GSE21653	GSM540232	BC	31	1	5.78	9.88	9.85	9.03	9.15	6.17
GSE21653	GSM540169	BC	32	1	4.95	10.03	8.98	8.26	9.54	5.78
GSE21653	GSM540153	BC	33	1	6.04	8.95	9.76	8.85	8.96	6.02
GSE21653	GSM540202	BC	34	1	5.58	9.85	9.74	8.74	9.04	6.15
GSE21653	GSM540334	BC	35	1	5.32	9.15	9.49	9.88	8.93	6.55
GSE21653	GSM540207	BC	36	1	7.52	8.83	9.19	9.01	8.68	6.83
GSE21653	GSM540335	BC	37	1	5.43	9.46	9.63	8.91	9.15	6.44
GSE21653	GSM540191	BC	38	1	6.51	9.64	9.29	9.69	8.85	6.94
GSE21653	GSM540134	BC	39	1	7.29	9.01	9.41	9.91	8.92	6.85
GSE21653	GSM540135	BC	40	1	5.64	10.06	9.73	8.81	9.19	6.25
GSE21653	GSM540133	BC	41	1	6.04	9.68	9.76	8.85	8.55	6.48
GSE21653	GSM540173	BC	42	1	6.99	9.77	9.18	10.06	9.65	6.79

GSE21653	GSM540175	BC	43	1	5.88	10.02	9.61	9.83	8.99	6.79
GSE21653	GSM540190	BC	44	1	5.61	9.44	8.76	9.23	9.27	6.27
GSE21653	GSM540194	BC	45	1	4.64	9.57	9.58	9.11	9.45	5.70
GSE21653	GSM540204	BC	46	1	5.13	9.75	9.74	7.81	9.47	5.98
GSE21653	GSM540136	BC	47	1	4.39	9.79	9.65	9.01	8.93	5.86
GSE21653	GSM540223	BC	48	1	5.36	9.76	9.04	7.89	9.33	5.98
GSE21653	GSM540210	BC	49	1	6.79	9.03	10.00	10.06	8.71	6.73
GSE21653	GSM540267	BC	50	1	7.58	9.16	9.50	10.80	9.50	8.62
GSE31448	GSM781454	BC	51	1	5.64	9.90	9.87	9.62	8.94	5.98
GSE31448	GSM781405	BC	52	1	6.48	9.49	9.63	9.67	8.84	6.92
GSE31448	GSM781389	BC	53	1	5.86	9.96	10.58	9.82	8.98	6.77
GSE31448	GSM781412	BC	54	1	7.06	9.81	9.66	10.09	9.02	7.66
GSE31448	GSM781553	BC	55	1	6.88	9.96	10.60	10.74	8.53	6.64
GSE31448	GSM781436	BC	56	1	6.30	9.85	10.30	10.80	8.73	6.34
GSE31448	GSM781547	BC	57	1	5.39	9.98	9.48	8.89	9.14	6.43
GSE31448	GSM781367	BC	58	1	6.02	9.48	9.64	8.84	8.94	6.00
GSE31448	GSM781404	BC	59	1	5.61	9.79	9.21	9.22	9.26	6.25
GSE31448	GSM781549	BC	60	1	4.81	9.92	9.14	9.67	9.20	6.49
GSE31448	GSM781529	BC	61	1	4.95	9.70	9.33	9.69	8.71	6.02
GSE31448	GSM781446	BC	62	1	5.75	10.04	9.62	9.01	9.14	6.13
GSE31448	GSM781481	BC	63	1	7.56	9.78	9.63	10.78	9.48	8.60
GSE31448	GSM781411	BC	64	1	6.23	10.08	9.74	9.36	9.16	6.91
GSE31448	GSM781533	BC	65	1	5.09	9.87	9.75	10.51	8.63	6.09
GSE31448	GSM781437	BC	66	1	5.36	9.77	9.76	7.88	9.31	5.95
GSE31448	GSM781424	BC	67	1	6.77	9.25	9.29	10.04	8.70	6.70
GSE31448	GSM781546	BC	68	1	5.32	9.15	10.27	9.86	8.92	6.54
GSE31448	GSM781527	BC	69	1	5.49	9.69	9.77	9.92	8.67	5.86
GSE31448	GSM781347	BC	70	1	6.02	9.58	9.60	8.83	8.53	6.44
GSE31448	GSM781406	BC	71	1	6.60	8.85	9.21	8.90	8.97	5.78
GSE31448	GSM781350	BC	72	1	4.32	9.45	8.78	9.00	8.91	5.81
GSE31448	GSM781418	BC	73	1	5.09	9.60	9.77	7.79	9.45	5.95
GSE31448	GSM781416	BC	74	1	5.55	9.89	9.69	8.73	9.03	6.13
GSE31448	GSM781348	BC	75	1	7.27	9.78	9.05	9.89	8.91	6.83
GSE31448	GSM781530	BC	76	1	5.46	9.03	9.42	10.51	7.49	7.15
GSE31448	GSM781383	BC	77	1	4.91	9.66	9.30	8.24	9.53	5.75
GSE31448	GSM781387	BC	78	1	6.98	10.05	8.99	10.04	9.64	6.78
GSE31448	GSM781349	BC	79	1	5.61	8.97	9.78	8.79	9.17	6.21
GSE31448	GSM781421	BC	80	1	7.50	10.37	10.11	9.00	8.66	6.82
GSE31448	GSM781426	BC	81	1	5.64	9.74	9.87	10.07	8.88	5.91
GSE31448	GSM781408	BC	82	1	4.64	9.76	9.94	9.10	9.44	5.67
GSE16446	GSM411302	BC	83	2	7.31	9.99	9.48	11.82	8.23	8.40
GSE16446	GSM411304	BC	84	2	7.16	9.71	9.46	11.23	8.57	7.44
GSE16446	GSM411314	BC	85	2	6.07	9.75	9.35	10.38	8.10	6.94
GSE16446	GSM411315	BC	86	2	6.00	9.24	9.73	10.41	8.33	6.94
GSE16446	GSM411320	BC	87	2	9.06	9.84	9.14	10.08	8.80	8.26
GSE16446	GSM411323	BC	88	2	7.32	9.39	9.20	10.43	9.53	8.65

GSE16446	GSM411325	BC	89	2	7.75	10.44	10.26	10.27	8.31	7.75
GSE16446	GSM411330	BC	90	2	6.70	9.03	10.35	10.66	8.16	6.93
GSE16446	GSM411339	BC	91	2	7.39	8.72	8.54	10.72	9.04	7.86
GSE16446	GSM411341	BC	92	2	6.46	9.25	9.34	10.93	8.91	7.77
GSE16446	GSM411343	BC	93	2	6.71	10.41	10.44	10.49	8.25	7.37
GSE16446	GSM411349	BC	94	2	6.70	10.13	9.12	10.79	8.69	6.75
GSE16446	GSM411356	BC	95	2	9.27	9.32	9.67	10.97	8.27	8.30
GSE16446	GSM411358	BC	96	2	7.65	9.92	9.64	10.82	8.27	7.64
GSE16446	GSM411362	BC	97	2	7.54	9.63	9.00	11.16	9.19	8.32
GSE16446	GSM411383	BC	98	2	8.36	9.36	10.03	10.69	9.43	7.43
GSE16446	GSM411388	BC	99	2	8.68	9.54	9.39	10.73	9.36	7.18
GSE16446	GSM411393	BC	100	2	8.36	9.89	10.53	10.87	9.21	7.64
GSE16446	GSM411395	BC	101	2	6.71	9.46	9.72	12.16	8.54	6.52
GSE16446	GSM411402	BC	102	2	9.20	9.32	9.99	10.43	8.98	8.80
GSE17907	GSM447213	BC	103	2	8.31	9.75	9.27	10.06	9.18	7.38
GSE17907	GSM447214	BC	104	2	7.83	10.03	9.63	8.92	8.61	7.44
GSE17907	GSM447217	BC	105	2	6.57	9.72	9.71	8.66	9.17	6.64
GSE17907	GSM447222	BC	106	2	6.89	10.10	9.43	8.91	9.39	6.34
GSE17907	GSM447228	BC	107	2	6.29	9.10	9.63	7.57	8.97	6.17
GSE17907	GSM447242	BC	108	2	7.75	9.88	10.15	9.26	9.44	6.98
GSE17907	GSM447207	BC	109	2	6.07	7.75	8.28	11.01	9.10	7.53
GSE17907	GSM447208	BC	110	2	7.36	8.91	9.01	8.57	8.87	7.03
GSE17907	GSM447227	BC	111	2	6.49	9.98	9.47	8.57	8.87	5.95
GSE17907	GSM447243	BC	112	2	7.12	9.48	9.85	7.65	8.77	6.11
GSE20713	GSM519768	BC	113	2	7.60	8.99	8.96	10.55	9.98	7.25
GSE20713	GSM519780	BC	114	2	6.13	9.20	9.34	10.54	9.19	6.00
GSE20713	GSM519781	BC	115	2	7.94	9.68	10.75	10.11	9.27	6.92
GSE20713	GSM519785	BC	116	2	6.60	9.68	10.19	10.90	9.08	6.97
GSE20713	GSM519793	BC	117	2	6.32	9.78	9.23	10.08	9.41	6.60
GSE21653	GSM540316	BC	118	2	8.19	9.30	9.45	9.23	9.03	6.86
GSE21653	GSM540320	BC	119	2	5.52	10.29	9.11	10.68	8.87	6.77
GSE21653	GSM540306	BC	120	2	7.73	9.72	10.16	9.35	8.67	6.71
GSE21653	GSM540166	BC	121	2	7.14	8.19	8.37	8.98	8.90	6.83
GSE21653	GSM540245	BC	122	2	7.32	9.17	9.86	9.33	9.58	6.61
GSE21653	GSM540336	BC	123	2	8.14	9.85	9.72	8.82	9.23	7.51
GSE21653	GSM540226	BC	124	2	7.61	9.74	9.33	9.11	8.55	6.48
GSE21653	GSM540266	BC	125	2	7.48	10.37	9.93	11.40	9.47	7.12
GSE21653	GSM540152	BC	126	2	7.79	9.00	9.57	9.52	8.55	6.82
GSE21653	GSM540231	BC	127	2	6.36	9.51	10.34	7.32	7.77	7.13
GSE21653	GSM540295	BC	128	2	7.82	9.27	9.33	8.39	8.89	7.04
GSE21653	GSM540201	BC	129	2	9.16	9.89	9.83	8.41	8.60	7.58
GSE21653	GSM540108	BC	130	2	6.49	9.97	9.34	8.57	8.87	5.95
GSE21653	GSM540299	BC	131	2	7.16	9.79	9.55	10.56	9.20	6.30
GSE21653	GSM540131	BC	132	2	7.50	9.03	9.54	9.66	8.88	7.10
GSE21653	GSM540139	BC	133	2	5.00	9.76	9.41	8.57	7.76	5.17
GSE21653	GSM540149	BC	134	2	7.07	9.07	9.91	9.73	9.59	6.92

GSE21653	GSM540333	BC	135	2	6.75	9.68	9.70	9.39	8.99	6.92
GSE21653	GSM540313	BC	136	2	6.66	10.47	10.37	10.31	8.78	7.34
GSE21653	GSM540239	BC	137	2	7.00	10.43	10.25	9.55	9.55	7.08
GSE21653	GSM540227	BC	138	2	5.75	9.81	9.36	8.27	9.50	6.09
GSE21653	GSM540144	BC	139	2	5.09	9.51	9.97	9.02	9.41	6.29
GSE21653	GSM540229	BC	140	2	7.38	9.56	9.51	8.87	9.12	6.86
GSE21653	GSM540233	BC	141	2	5.13	9.45	9.55	9.01	9.12	6.15
GSE21653	GSM540225	BC	142	2	6.67	8.92	9.51	8.65	9.06	7.16
GSE21653	GSM540330	BC	143	2	4.95	8.95	9.33	8.90	8.50	6.30
GSE21653	GSM540171	BC	144	2	7.37	9.46	9.68	8.57	8.87	7.03
GSE21653	GSM540179	BC	145	2	6.64	9.65	9.64	10.50	8.98	6.57
GSE21653	GSM540322	BC	146	2	5.49	8.79	8.85	9.91	8.62	6.25
GSE21653	GSM540241	BC	147	2	5.86	8.88	9.59	9.29	9.07	6.23
GSE21653	GSM540203	BC	148	2	5.29	9.01	10.33	9.23	8.54	6.02
GSE21653	GSM540145	BC	149	2	5.95	9.19	8.61	8.79	9.29	6.58
GSE21653	GSM540193	BC	150	2	6.11	8.69	8.50	10.44	8.80	6.86
GSE21653	GSM540258	BC	151	2	6.15	9.71	9.73	9.30	8.40	5.61
GSE21653	GSM540230	BC	152	2	7.03	8.75	8.83	8.19	9.11	6.07
GSE21653	GSM540279	BC	153	2	6.07	10.30	10.20	11.01	9.10	7.53
GSE21653	GSM540275	BC	154	2	6.51	9.85	9.71	8.83	8.67	6.69
GSE21653	GSM540220	BC	155	2	5.81	9.30	9.40	10.38	9.44	6.64
GSE21653	GSM540185	BC	156	2	7.31	8.90	9.73	8.74	8.90	6.88
GSE21653	GSM540262	BC	157	2	7.06	9.20	10.06	10.09	9.57	8.08
GSE21653	GSM540243	BC	158	2	6.66	9.43	9.43	9.25	8.98	6.43
GSE21653	GSM540247	BC	159	2	6.71	9.35	9.41	9.70	9.06	7.00
GSE21653	GSM540286	BC	160	2	6.11	9.29	9.79	7.79	9.38	6.52
GSE21653	GSM540344	BC	161	2	4.91	10.87	9.55	10.37	9.28	6.21
GSE21653	GSM540287	BC	162	2	7.66	9.56	9.29	8.75	9.08	6.57
GSE21653	GSM540199	BC	163	2	8.24	9.98	10.23	9.00	9.89	7.07
GSE21653	GSM540328	BC	164	2	4.58	9.83	9.76	8.68	8.96	5.83
GSE21653	GSM540296	BC	165	2	6.91	9.57	9.44	8.29	9.21	6.09
GSE21653	GSM540338	BC	166	2	6.25	9.42	8.71	8.52	9.22	6.15
GSE21653	GSM540205	BC	167	2	5.09	9.32	8.78	8.68	9.02	6.34
GSE21653	GSM540350	BC	168	2	6.43	10.51	9.50	11.09	8.93	6.89
GSE21653	GSM540112	BC	169	2	7.21	8.81	9.25	10.23	9.76	6.64
GSE21653	GSM540329	BC	170	2	6.00	9.49	8.22	9.38	8.95	6.29
GSE21653	GSM540347	BC	171	2	7.28	9.61	8.47	8.97	10.23	7.06
GSE21653	GSM540349	BC	172	2	8.06	9.30	9.19	9.94	9.54	7.71
GSE21653	GSM540129	BC	173	2	7.41	9.32	10.29	8.36	9.40	7.12
GSE21653	GSM540200	BC	174	2	7.07	9.26	9.40	8.73	8.97	6.70
GSE21653	GSM540150	BC	175	2	5.86	10.10	9.30	8.80	8.89	6.13
GSE21653	GSM540189	BC	176	2	5.64	8.69	9.52	8.96	8.71	7.03
GSE21653	GSM540188	BC	177	2	5.81	10.11	9.32	9.73	9.03	6.43
GSE21653	GSM540234	BC	178	2	6.89	8.98	9.35	10.44	8.46	6.36
GSE21653	GSM540211	BC	179	2	5.17	9.45	9.44	9.18	9.07	5.98
GSE21653	GSM540170	BC	180	2	6.29	9.32	9.42	9.63	9.14	7.75

GSE21653	GSM540180	BC	181	2	6.57	9.62	8.48	8.66	9.17	6.66
GSE21653	GSM540251	BC	182	2	8.31	9.56	9.41	10.06	9.18	7.38
GSE21653	GSM540354	BC	183	2	7.75	9.66	9.66	9.92	8.41	6.36
GSE21653	GSM540339	BC	184	2	6.29	9.84	9.77	9.17	9.11	6.44
GSE21653	GSM540162	BC	185	2	7.59	9.51	8.24	9.73	9.56	6.97
GSE21653	GSM540327	BC	186	2	7.70	8.23	8.41	10.02	8.99	7.46
GSE21653	GSM540151	BC	187	2	8.51	8.92	9.75	9.07	8.75	8.18
GSE21653	GSM540183	BC	188	2	9.33	9.01	9.58	9.62	8.93	8.30
GSE21653	GSM540237	BC	189	2	6.44	8.72	8.54	9.47	8.91	7.09
GSE21653	GSM540331	BC	190	2	7.01	9.30	9.81	9.15	8.83	7.83
GSE21653	GSM540255	BC	191	2	7.86	9.78	9.43	10.24	9.04	7.73
GSE21653	GSM540117	BC	192	2	8.84	7.79	8.32	9.84	9.11	9.03
GSE21653	GSM540359	BC	193	2	10.79	10.39	9.95	10.48	9.94	8.88
GSE21653	GSM540364	BC	194	2	7.88	9.28	9.35	10.15	8.80	6.94
GSE21653	GSM540156	BC	195	2	7.46	9.38	10.04	9.60	9.97	8.57
GSE21653	GSM540118	BC	196	2	8.08	9.48	9.74	9.65	8.97	6.94
GSE31448	GSM781525	BC	197	2	6.64	8.81	8.87	10.29	8.76	7.32
GSE31448	GSM781548	BC	198	2	8.12	8.93	9.03	8.79	9.21	7.48
GSE31448	GSM781402	BC	199	2	5.78	9.76	9.29	9.71	9.01	6.41
GSE31448	GSM781453	BC	200	2	6.98	9.32	9.21	9.54	9.54	7.06
GSE31448	GSM781397	BC	201	2	9.31	9.89	10.17	9.61	8.91	8.29
GSE31448	GSM781443	BC	202	2	7.36	9.22	9.36	8.85	9.11	6.83
GSE31448	GSM781541	BC	203	2	5.98	9.03	10.35	9.36	8.93	6.27
GSE31448	GSM781556	BC	204	2	4.86	9.04	9.56	10.36	9.27	6.19
GSE31448	GSM781534	BC	205	2	5.43	10.14	9.47	9.88	8.58	6.21
GSE31448	GSM781551	BC	206	2	6.25	8.90	9.61	9.15	9.09	6.43
GSE31448	GSM781439	BC	207	2	6.66	9.47	9.57	8.64	9.04	7.14
GSE31448	GSM781353	BC	208	2	4.95	9.84	9.38	8.53	7.72	5.09
GSE31448	GSM781358	BC	209	2	5.04	8.77	8.85	9.00	9.39	6.27
GSE31448	GSM781465	BC	210	2	8.30	9.73	9.74	10.05	9.16	7.37
GSE31448	GSM781489	BC	211	2	6.48	9.01	8.97	8.81	8.65	6.66
GSE31448	GSM781444	BC	212	2	7.01	10.44	10.26	8.17	9.09	6.04
GSE31448	GSM781542	BC	213	2	4.91	9.50	9.87	8.88	8.48	6.29
GSE31448	GSM781576	BC	214	2	7.86	9.43	8.72	10.13	8.79	6.93
GSE31448	GSM781332	BC	215	2	8.07	9.18	9.87	9.64	8.95	6.93
GSE31448	GSM781365	BC	216	2	8.49	10.52	9.52	9.05	8.73	8.16
GSE31448	GSM781559	BC	217	2	7.26	8.83	9.26	8.96	10.22	7.04
GSE31448	GSM781370	BC	218	2	7.44	10.31	10.21	9.58	9.95	8.55
GSE31448	GSM781469	BC	219	2	7.84	9.91	9.85	10.22	9.02	7.71
GSE31448	GSM781445	BC	220	2	6.30	9.73	9.72	7.28	7.73	7.09
GSE31448	GSM781399	BC	221	2	7.29	9.37	9.42	8.72	8.89	6.86
GSE31448	GSM781413	BC	222	2	8.23	9.87	9.73	8.98	9.87	7.04
GSE31448	GSM781394	BC	223	2	6.54	9.59	9.46	8.64	9.16	6.63
GSE31448	GSM781363	BC	224	2	7.04	9.09	9.93	9.71	9.57	6.88
GSE31448	GSM781415	BC	225	2	9.15	10.00	9.36	8.40	8.58	7.55
GSE31448	GSM781448	BC	226	2	6.87	9.28	9.41	10.42	8.44	6.32

GSE31448	GSM781545	BC	227	2	6.74	8.71	9.54	9.37	8.97	6.89
GSE31448	GSM781499	BC	228	2	6.09	9.47	9.69	7.78	9.36	6.49
GSE31448	GSM781550	BC	229	2	6.21	10.05	9.65	8.50	9.19	6.13
GSE31448	GSM781364	BC	230	2	5.83	9.31	9.46	8.78	8.88	6.11
GSE31448	GSM781393	BC	231	2	6.61	9.33	10.30	10.49	8.96	6.54
GSE31448	GSM781345	BC	232	2	7.48	9.58	9.31	9.65	8.86	7.08
GSE31448	GSM781508	BC	233	2	6.89	9.21	8.64	8.27	9.19	6.07
GSE31448	GSM781539	BC	234	2	7.68	9.81	9.57	10.00	8.97	7.44
GSE31448	GSM781571	BC	235	2	10.78	9.52	9.98	10.47	9.93	8.87
GSE31448	GSM781493	BC	236	2	6.04	9.90	10.55	11.00	9.09	7.52
GSE31448	GSM781359	BC	237	2	5.93	9.53	10.35	8.77	9.26	6.55
GSE31448	GSM781403	BC	238	2	5.61	9.58	9.53	8.94	8.69	7.01
GSE31448	GSM781425	BC	239	2	5.13	9.34	8.80	9.16	9.05	5.95
GSE31448	GSM781417	BC	240	2	5.25	9.75	9.35	9.22	8.52	5.98
GSE31448	GSM781566	BC	241	2	7.72	9.99	9.48	9.90	8.39	6.32
GSE31448	GSM781532	BC	242	2	5.49	8.93	9.54	10.66	8.84	6.74
GSE31448	GSM781326	BC	243	2	7.20	9.12	9.65	10.22	9.75	6.63
GSE31448	GSM781440	BC	244	2	7.58	9.21	10.08	9.09	8.53	6.46
GSE31448	GSM781441	BC	245	2	5.73	9.70	9.72	8.24	9.48	6.07
GSE31448	GSM781455	BC	246	2	5.83	9.80	9.25	9.26	9.05	6.19
GSE31448	GSM781385	BC	247	2	7.35	9.34	10.01	8.55	8.85	7.01
GSE31448	GSM781414	BC	248	2	7.03	9.86	9.73	8.71	8.95	6.67
GSE31448	GSM781322	BC	249	2	6.46	10.01	10.25	8.53	8.83	5.91
GSE31448	GSM781472	BC	250	2	6.11	9.74	10.18	9.28	8.38	5.58
GSE31448	GSM781407	BC	251	2	6.09	10.89	9.57	10.42	8.78	6.83
GSE31448	GSM781461	BC	252	2	6.70	10.48	10.38	9.69	9.05	6.98
GSE31448	GSM781451	BC	253	2	6.43	9.69	10.76	9.46	8.89	7.07
GSE31448	GSM781380	BC	254	2	7.12	10.31	9.12	8.97	8.89	6.82
GSE31448	GSM781507	BC	255	2	7.80	9.69	10.20	8.37	8.88	7.02
GSE31448	GSM781376	BC	256	2	7.57	10.24	10.07	9.71	9.54	6.94
GSE31448	GSM781518	BC	257	2	7.71	9.63	10.27	9.33	8.66	6.69
GSE31448	GSM781500	BC	258	2	7.64	8.95	9.74	8.73	9.06	6.55
GSE31448	GSM781511	BC	259	2	7.14	10.15	8.96	10.55	9.18	6.29
GSE31448	GSM781331	BC	260	2	8.83	11.12	9.91	9.82	9.10	9.02
GSE31448	GSM781343	BC	261	2	7.38	10.23	9.70	8.34	9.39	7.10
GSE31448	GSM781543	BC	262	2	6.99	9.58	10.10	9.13	8.81	7.81
GSE31448	GSM781480	BC	263	2	7.47	9.26	10.17	11.39	9.46	7.10
GSE31448	GSM781540	BC	264	2	4.52	9.71	9.85	8.66	8.95	5.81
GSE31448	GSM781434	BC	265	2	5.78	9.18	9.31	10.37	9.42	6.63
GSE31448	GSM781384	BC	266	2	6.27	9.49	10.34	9.61	9.13	7.72
GSE31448	GSM781476	BC	267	2	7.03	10.03	10.32	10.07	9.55	8.07
GSE31448	GSM781457	BC	268	2	6.63	9.87	9.98	9.23	8.96	6.41
GSE31448	GSM781366	BC	269	2	7.76	9.85	9.54	9.49	8.52	6.79
GSE31448	GSM781459	BC	270	2	7.30	9.92	10.45	9.32	9.57	6.60
GSE31448	GSM781447	BC	271	2	5.09	9.56	10.18	9.00	9.11	6.13
GSE31448	GSM781561	BC	272	2	8.04	9.70	9.30	9.92	9.53	7.69

GSE31448	GSM781419	BC	273	2	5.09	10.41	10.44	8.67	9.01	6.32
GSE31448	GSM781528	BC	274	2	8.17	9.85	9.68	9.21	9.01	6.83
GSE31448	GSM781562	BC	275	2	6.39	9.65	9.35	11.07	8.91	6.87
GSE13787	GSM346882	BC	276	3	9.28	8.97	9.07	11.02	9.78	8.84
GSE13787	GSM346883	BC	277	3	8.18	9.01	9.16	11.77	8.98	8.18
GSE13787	GSM346884	BC	278	3	9.08	10.43	9.29	10.78	9.73	9.08
GSE13787	GSM346885	BC	279	3	7.88	9.36	9.57	9.86	9.54	6.82
GSE13787	GSM346886	BC	280	3	9.01	9.44	9.86	10.19	9.59	7.38
GSE13787	GSM346887	BC	281	3	8.23	9.78	9.13	12.25	9.65	8.27
GSE13787	GSM346888	BC	282	3	8.80	9.40	9.16	10.40	9.98	8.32
GSE13787	GSM346889	BC	283	3	8.57	9.68	9.67	10.30	9.62	8.66
GSE13787	GSM346890	BC	284	3	7.42	9.72	9.70	10.17	8.70	6.78
GSE13787	GSM346891	BC	285	3	6.64	10.27	9.45	10.49	9.01	7.00
GSE13787	GSM346892	BC	286	3	7.76	10.31	10.04	9.20	8.69	6.73
GSE13787	GSM346893	BC	287	3	8.26	10.02	9.68	10.77	8.54	7.44
GSE13787	GSM346894	BC	288	3	6.79	10.02	9.32	10.97	8.50	6.95
GSE13787	GSM346895	BC	289	3	7.21	8.95	9.55	11.18	9.96	7.31
GSE13787	GSM346896	BC	290	3	7.55	9.75	10.21	10.53	8.93	7.89
GSE13787	GSM346897	BC	291	3	7.67	9.89	9.41	11.02	8.71	7.56
GSE13787	GSM346898	BC	292	3	7.30	10.33	9.46	10.66	9.26	7.24
GSE13787	GSM346899	BC	293	3	6.81	9.57	9.21	10.60	8.86	7.86
GSE13787	GSM346900	BC	294	3	8.13	9.20	10.04	10.24	9.12	8.85
GSE13787	GSM346901	BC	295	3	8.33	9.74	9.68	10.96	9.16	8.34
GSE13787	GSM346902	BC	296	3	7.77	9.22	9.29	10.61	8.85	7.52
GSE13787	GSM346903	BC	297	3	8.57	9.48	10.22	11.90	8.77	8.87
GSE13787	GSM346904	BC	298	3	8.52	9.05	9.94	9.73	9.37	8.79
GSE16446	GSM411290	BC	299	3	8.97	9.07	10.02	11.81	9.85	9.34
GSE16446	GSM411291	BC	300	3	8.09	9.08	9.71	11.08	9.22	7.19
GSE16446	GSM411292	BC	301	3	7.86	9.02	9.61	10.48	9.04	7.29
GSE16446	GSM411293	BC	302	3	6.92	9.61	9.81	9.78	8.75	7.69
GSE16446	GSM411294	BC	303	3	9.60	9.61	9.79	10.83	8.62	9.10
GSE16446	GSM411295	BC	304	3	8.06	8.96	8.54	11.66	8.67	7.93
GSE16446	GSM411296	BC	305	3	9.74	9.28	10.00	11.04	9.07	9.06
GSE16446	GSM411297	BC	306	3	8.31	9.38	9.58	10.60	9.13	8.48
GSE16446	GSM411298	BC	307	3	9.80	9.56	10.13	11.32	9.58	7.45
GSE16446	GSM411299	BC	308	3	7.15	9.48	10.13	10.29	9.08	7.52
GSE16446	GSM411300	BC	309	3	7.17	9.21	9.45	9.95	8.23	6.46
GSE16446	GSM411301	BC	310	3	7.13	10.32	8.78	10.85	9.35	7.38
GSE16446	GSM411303	BC	311	3	8.34	9.72	10.08	11.08	8.92	7.64
GSE16446	GSM411305	BC	312	3	8.64	9.85	9.46	10.57	8.68	7.20
GSE16446	GSM411306	BC	313	3	8.16	8.68	9.61	10.87	9.42	8.65
GSE16446	GSM411307	BC	314	3	7.87	9.88	9.23	10.92	8.31	7.51
GSE16446	GSM411308	BC	315	3	9.06	9.65	10.17	11.26	8.56	7.77
GSE16446	GSM411309	BC	316	3	8.50	9.41	8.91	10.22	9.05	8.35
GSE16446	GSM411310	BC	317	3	8.34	9.61	9.18	11.57	9.31	8.08
GSE16446	GSM411311	BC	318	3	8.43	10.15	9.76	10.62	9.62	7.31

GSE16446	GSM411312	BC	319	3	9.45	9.19	9.98	11.36	8.63	8.69
GSE16446	GSM411313	BC	320	3	9.43	9.59	9.97	11.06	9.70	8.47
GSE16446	GSM411317	BC	321	3	6.66	9.86	9.42	10.27	8.85	7.36
GSE16446	GSM411318	BC	322	3	8.33	9.61	9.86	10.45	9.13	8.50
GSE16446	GSM411319	BC	323	3	8.45	8.95	9.42	10.02	8.88	7.52
GSE16446	GSM411321	BC	324	3	7.89	9.56	9.80	10.71	9.17	7.91
GSE16446	GSM411322	BC	325	3	7.26	10.18	9.68	10.27	8.17	7.69
GSE16446	GSM411324	BC	326	3	9.98	9.97	9.82	9.54	9.07	8.31
GSE16446	GSM411326	BC	327	3	8.65	10.16	9.70	10.23	8.38	8.19
GSE16446	GSM411327	BC	328	3	9.14	9.51	9.96	11.18	10.00	8.24
GSE16446	GSM411328	BC	329	3	7.90	9.60	10.20	10.28	9.28	8.22
GSE16446	GSM411329	BC	330	3	7.06	9.62	9.90	11.71	8.73	7.08
GSE16446	GSM411331	BC	331	3	6.39	9.63	9.15	10.71	8.58	7.25
GSE16446	GSM411332	BC	332	3	8.13	9.24	9.80	11.24	9.86	9.15
GSE16446	GSM411333	BC	333	3	8.75	10.71	9.89	10.67	8.78	8.49
GSE16446	GSM411334	BC	334	3	8.65	9.08	9.48	10.43	9.50	8.34
GSE16446	GSM411335	BC	335	3	7.85	10.60	10.26	11.18	9.27	8.34
GSE16446	GSM411337	BC	336	3	7.17	9.68	9.45	10.99	9.30	7.50
GSE16446	GSM411338	BC	337	3	8.54	9.63	9.18	10.26	9.15	8.66
GSE16446	GSM411340	BC	338	3	7.79	9.55	10.02	10.76	8.88	7.19
GSE16446	GSM411342	BC	339	3	9.67	9.84	9.80	11.49	8.69	7.52
GSE16446	GSM411344	BC	340	3	9.08	10.38	9.48	10.88	9.17	8.91
GSE16446	GSM411346	BC	341	3	8.34	9.43	9.12	9.98	8.22	6.73
GSE16446	GSM411347	BC	342	3	8.65	9.60	9.55	11.42	9.50	8.63
GSE16446	GSM411348	BC	343	3	9.19	9.88	9.23	10.46	9.74	8.45
GSE16446	GSM411350	BC	344	3	8.04	9.20	9.82	10.51	8.88	9.66
GSE16446	GSM411351	BC	345	3	7.83	9.65	9.26	11.25	8.73	7.51
GSE16446	GSM411352	BC	346	3	8.87	9.26	9.71	10.87	8.84	8.32
GSE16446	GSM411353	BC	347	3	9.40	10.32	9.18	10.19	9.28	7.25
GSE16446	GSM411354	BC	348	3	10.06	10.14	10.36	11.01	9.14	8.48
GSE16446	GSM411355	BC	349	3	8.06	10.03	10.00	10.72	9.30	7.29
GSE16446	GSM411357	BC	350	3	9.20	10.18	9.81	10.54	9.55	7.86
GSE16446	GSM411359	BC	351	3	8.60	9.72	9.13	10.38	8.07	8.39
GSE16446	GSM411360	BC	352	3	9.37	9.91	9.57	10.99	9.24	8.11
GSE16446	GSM411361	BC	353	3	10.04	10.27	9.53	10.61	9.08	8.27
GSE16446	GSM411363	BC	354	3	8.15	9.71	9.77	11.62	9.18	8.17
GSE16446	GSM411364	BC	355	3	7.98	9.91	9.85	9.96	9.67	8.05
GSE16446	GSM411365	BC	356	3	7.30	9.68	9.03	11.55	8.61	6.93
GSE16446	GSM411366	BC	357	3	7.89	9.25	9.16	11.07	8.37	7.20
GSE16446	GSM411368	BC	358	3	8.57	9.91	9.41	11.80	9.10	9.55
GSE16446	GSM411369	BC	359	3	6.92	9.36	9.21	10.83	8.62	7.54
GSE16446	GSM411370	BC	360	3	8.70	9.55	9.04	10.15	9.65	7.50
GSE16446	GSM411371	BC	361	3	9.44	10.57	9.17	10.29	9.95	8.62
GSE16446	GSM411372	BC	362	3	7.16	9.52	9.34	9.92	9.84	7.48
GSE16446	GSM411373	BC	363	3	9.33	10.13	9.22	11.00	8.17	7.88
GSE16446	GSM411374	BC	364	3	8.16	9.23	8.70	10.38	8.91	8.72

GSE16446	GSM411375	BC	365	3	7.93	9.73	8.86	10.62	8.73	8.62
GSE16446	GSM411376	BC	366	3	8.27	9.64	9.14	11.38	8.43	7.88
GSE16446	GSM411377	BC	367	3	8.43	10.11	8.52	11.40	8.42	8.21
GSE16446	GSM411378	BC	368	3	8.82	10.33	9.07	10.39	8.97	9.06
GSE16446	GSM411379	BC	369	3	8.26	9.48	8.77	10.88	9.70	8.66
GSE16446	GSM411380	BC	370	3	7.44	9.53	9.61	10.48	9.89	7.81
GSE16446	GSM411381	BC	371	3	9.32	9.92	9.30	11.08	8.68	9.87
GSE16446	GSM411382	BC	372	3	8.25	9.46	9.02	10.52	9.64	8.22
GSE16446	GSM411384	BC	373	3	9.33	10.29	9.75	11.13	9.41	8.35
GSE16446	GSM411386	BC	374	3	8.09	9.74	9.86	10.31	8.87	8.16
GSE16446	GSM411387	BC	375	3	9.04	9.96	9.99	11.10	9.74	8.54
GSE16446	GSM411389	BC	376	3	6.49	10.07	9.83	10.03	9.09	6.92
GSE16446	GSM411390	BC	377	3	7.99	9.93	9.33	11.49	8.29	7.41
GSE16446	GSM411394	BC	378	3	8.34	10.12	9.82	9.36	9.13	9.56
GSE16446	GSM411396	BC	379	3	8.46	10.20	9.03	10.20	8.61	7.75
GSE16446	GSM411397	BC	380	3	9.01	10.42	9.95	10.00	9.72	9.15
GSE16446	GSM411398	BC	381	3	8.14	10.42	9.58	10.92	9.75	8.75
GSE16446	GSM411399	BC	382	3	5.61	10.12	9.32	10.78	8.53	6.44
GSE16446	GSM411400	BC	383	3	9.17	9.57	8.84	10.09	9.40	7.98
GSE16446	GSM411401	BC	384	3	9.00	9.65	9.88	9.07	8.92	8.02
GSE16446	GSM411403	BC	385	3	7.86	9.68	9.62	10.71	9.38	7.37
GSE16446	GSM411404	BC	386	3	8.37	9.01	9.10	11.21	8.76	8.12
GSE16446	GSM411406	BC	387	3	7.81	9.38	9.41	10.83	8.55	7.38
GSE16446	GSM411407	BC	388	3	6.75	9.34	9.40	10.87	8.29	7.20
GSE16446	GSM411408	BC	389	3	7.75	10.32	9.64	10.48	9.74	7.43
GSE16446	GSM411409	BC	390	3	7.92	9.42	9.26	10.23	9.26	7.11
GSE17907	GSM447225	BC	391	3	6.91	10.58	9.39	10.69	8.85	7.83
GSE17907	GSM447197	BC	392	3	7.42	10.41	9.89	11.13	8.96	7.40
GSE17907	GSM447198	BC	393	3	10.03	9.52	8.85	9.72	8.33	8.04
GSE17907	GSM447199	BC	394	3	9.17	10.63	10.27	10.65	9.11	7.61
GSE17907	GSM447200	BC	395	3	7.34	9.96	9.95	10.14	9.58	8.64
GSE17907	GSM447201	BC	396	3	5.58	9.73	10.20	7.97	9.45	6.57
GSE17907	GSM447202	BC	397	3	7.16	9.65	9.47	9.49	9.24	7.76
GSE17907	GSM447203	BC	398	3	7.33	9.98	9.04	9.15	9.15	6.63
GSE17907	GSM447209	BC	399	3	7.35	9.80	8.93	9.93	9.43	7.20
GSE17907	GSM447210	BC	400	3	8.47	10.99	9.81	9.29	8.68	9.11
GSE17907	GSM447211	BC	401	3	8.67	9.78	9.35	9.44	8.80	8.52
GSE17907	GSM447212	BC	402	3	7.85	10.25	9.43	9.82	9.31	7.14
GSE17907	GSM447215	BC	403	3	9.65	10.17	10.88	9.48	8.82	6.93
GSE17907	GSM447219	BC	404	3	7.49	9.91	8.98	7.81	9.05	7.23
GSE17907	GSM447221	BC	405	3	8.78	9.30	8.75	10.55	8.55	7.03
GSE17907	GSM447226	BC	406	3	7.33	9.53	9.91	9.55	9.10	7.64
GSE17907	GSM447229	BC	407	3	7.91	10.65	9.85	8.72	9.86	7.57
GSE17907	GSM447230	BC	408	3	8.63	9.46	8.79	10.70	9.14	7.23
GSE17907	GSM447231	BC	409	3	5.91	9.94	8.89	8.90	9.76	7.34
GSE17907	GSM447232	BC	410	3	6.83	9.58	10.08	10.11	9.30	6.89

GSE17907	GSM447233	BC	411	3	7.78	10.12	9.47	8.70	8.86	6.54
GSE17907	GSM447236	BC	412	3	9.57	9.52	9.97	9.51	9.02	6.85
GSE17907	GSM447246	BC	413	3	9.17	10.10	9.97	10.18	8.41	8.39
GSE17907	GSM447247	BC	414	3	6.75	10.42	10.05	8.87	8.77	7.43
GSE17907	GSM447218	BC	415	3	8.22	10.27	9.97	8.80	9.50	7.43
GSE17907	GSM447234	BC	416	3	7.18	10.52	9.88	10.19	9.21	6.70
GSE17907	GSM447245	BC	417	3	7.90	10.64	9.64	8.75	9.06	7.06
GSE20713	GSM519722	BC	418	3	7.23	10.76	10.01	10.76	8.89	7.46
GSE20713	GSM519723	BC	419	3	7.33	10.13	10.06	9.93	9.13	7.28
GSE20713	GSM519724	BC	420	3	6.77	10.32	11.28	10.64	9.09	7.09
GSE20713	GSM519725	BC	421	3	6.15	9.79	9.63	10.57	9.15	5.95
GSE20713	GSM519726	BC	422	3	7.85	9.84	9.80	10.79	9.18	7.57
GSE20713	GSM519727	BC	423	3	8.40	9.67	9.27	9.29	8.82	6.44
GSE20713	GSM519728	BC	424	3	6.83	10.99	9.21	10.77	9.49	6.69
GSE20713	GSM519729	BC	425	3	9.23	9.58	9.65	10.75	9.95	8.32
GSE20713	GSM519730	BC	426	3	7.38	10.11	10.43	12.33	9.23	7.24
GSE20713	GSM519731	BC	427	3	7.48	10.03	8.97	10.58	9.15	6.48
GSE20713	GSM519732	BC	428	3	6.49	10.22	9.01	10.43	9.10	6.51
GSE20713	GSM519733	BC	429	3	6.66	10.19	10.58	11.02	9.84	6.92
GSE20713	GSM519734	BC	430	3	8.04	9.83	9.96	10.90	9.17	7.55
GSE20713	GSM519735	BC	431	3	5.09	8.96	10.14	10.12	8.90	5.36
GSE20713	GSM519737	BC	432	3	6.48	10.14	9.63	9.99	9.43	5.70
GSE20713	GSM519738	BC	433	3	7.29	9.71	9.95	10.72	8.83	6.97
GSE20713	GSM519739	BC	434	3	7.32	10.46	9.47	9.99	9.00	7.30
GSE20713	GSM519740	BC	435	3	6.19	10.03	8.97	9.95	9.24	7.02
GSE20713	GSM519741	BC	436	3	7.85	9.60	9.53	10.25	8.73	7.70
GSE20713	GSM519742	BC	437	3	7.01	9.98	9.92	10.74	9.34	6.61
GSE20713	GSM519743	BC	438	3	5.58	10.61	9.84	10.58	9.24	6.75
GSE20713	GSM519744	BC	439	3	6.09	10.54	9.66	9.94	8.85	6.61
GSE20713	GSM519745	BC	440	3	9.42	8.95	9.05	10.50	9.31	8.08
GSE20713	GSM519746	BC	441	3	8.01	10.31	9.44	10.86	9.09	7.71
GSE20713	GSM519747	BC	442	3	8.65	9.18	10.02	10.73	9.75	7.81
GSE20713	GSM519748	BC	443	3	6.13	9.88	9.58	10.57	9.49	6.54
GSE20713	GSM519749	BC	444	3	7.64	10.72	9.83	10.76	8.79	7.43
GSE20713	GSM519750	BC	445	3	6.98	10.60	9.13	11.41	9.20	7.21
GSE20713	GSM519751	BC	446	3	8.97	9.35	9.70	10.93	8.75	7.23
GSE20713	GSM519752	BC	447	3	7.03	9.65	9.39	11.84	9.23	7.52
GSE20713	GSM519753	BC	448	3	8.36	9.34	9.29	11.05	9.14	7.55
GSE20713	GSM519754	BC	449	3	7.02	10.16	10.03	11.43	9.06	6.83
GSE20713	GSM519755	BC	450	3	7.53	8.89	9.66	11.23	9.49	8.46
GSE20713	GSM519756	BC	451	3	8.28	9.94	9.69	10.32	8.75	7.70
GSE20713	GSM519757	BC	452	3	6.81	9.19	9.60	10.45	9.37	7.00
GSE20713	GSM519758	BC	453	3	5.98	9.71	9.68	10.64	9.62	6.74
GSE20713	GSM519759	BC	454	3	7.79	9.24	9.87	9.91	9.39	6.69
GSE20713	GSM519760	BC	455	3	8.70	9.06	9.69	10.27	8.59	7.59
GSE20713	GSM519761	BC	456	3	5.55	9.28	9.92	10.47	9.54	6.73

GSE20713	GSM519763	BC	457	3	6.60	9.76	10.04	10.15	9.15	6.29
GSE20713	GSM519764	BC	458	3	5.70	9.65	9.77	9.94	9.67	6.57
GSE20713	GSM519765	BC	459	3	4.81	9.65	9.82	10.58	9.18	6.25
GSE20713	GSM519767	BC	460	3	6.63	10.98	10.01	11.03	9.61	6.73
GSE20713	GSM519769	BC	461	3	7.97	9.56	9.27	10.31	9.49	7.41
GSE20713	GSM519770	BC	462	3	7.38	9.97	10.62	10.60	9.09	7.00
GSE20713	GSM519771	BC	463	3	8.07	9.62	9.08	11.45	8.97	7.56
GSE20713	GSM519773	BC	464	3	6.92	9.26	10.47	10.95	9.37	6.49
GSE20713	GSM519774	BC	465	3	9.27	10.23	9.69	10.97	9.59	7.43
GSE20713	GSM519775	BC	466	3	8.01	9.97	10.18	10.39	8.77	7.31
GSE20713	GSM519776	BC	467	3	8.68	9.20	9.27	10.52	9.35	8.32
GSE20713	GSM519777	BC	468	3	7.50	8.56	9.45	10.17	9.39	7.59
GSE20713	GSM519778	BC	469	3	7.11	10.29	10.03	10.74	9.20	6.66
GSE20713	GSM519779	BC	470	3	8.64	10.07	9.58	10.03	9.38	7.20
GSE20713	GSM519782	BC	471	3	6.98	9.81	9.35	10.02	9.07	6.43
GSE20713	GSM519783	BC	472	3	8.01	10.00	9.29	10.86	9.31	7.06
GSE20713	GSM519784	BC	473	3	8.07	10.08	10.55	10.02	9.81	7.19
GSE20713	GSM519786	BC	474	3	7.25	10.40	10.09	10.73	8.51	7.18
GSE20713	GSM519787	BC	475	3	7.97	9.40	9.21	10.17	9.68	7.52
GSE20713	GSM519792	BC	476	3	8.87	9.74	9.76	10.89	9.33	8.57
GSE20713	GSM519794	BC	477	3	8.04	9.60	10.23	9.73	8.87	8.19
GSE20713	GSM519796	BC	478	3	6.43	9.87	9.39	10.76	8.74	6.81
GSE20713	GSM519797	BC	479	3	7.71	9.92	10.53	10.13	8.69	6.63
GSE20713	GSM519799	BC	480	3	6.67	9.42	10.05	10.03	9.06	8.06
GSE20713	GSM519800	BC	481	3	8.17	8.69	8.98	10.38	9.25	7.16
GSE20713	GSM519801	BC	482	3	6.48	9.64	10.14	11.48	9.15	6.89
GSE20713	GSM519802	BC	483	3	7.87	9.67	9.89	10.26	9.39	8.08
GSE20713	GSM519803	BC	484	3	6.43	10.54	10.22	10.75	8.99	6.30
GSE20713	GSM519805	BC	485	3	6.19	9.24	9.10	10.47	9.23	6.48
GSE20713	GSM519806	BC	486	3	7.52	10.05	9.78	11.01	8.75	7.27
GSE20713	GSM519808	BC	487	3	7.40	9.85	10.14	9.69	8.36	7.64
GSE21653	GSM540311	BC	488	3	7.85	8.93	9.54	9.92	8.52	8.14
GSE21653	GSM540303	BC	489	3	8.78	10.26	10.19	8.71	8.66	7.75
GSE21653	GSM540142	BC	490	3	7.66	9.54	9.22	9.67	9.65	7.95
GSE21653	GSM540342	BC	491	3	8.64	9.90	9.98	9.91	8.99	7.83
GSE21653	GSM540148	BC	492	3	7.27	10.08	9.48	11.24	8.98	6.41
GSE21653	GSM540182	BC	493	3	8.04	8.98	8.64	9.60	8.47	7.07
GSE21653	GSM540277	BC	494	3	7.48	9.74	10.01	9.60	8.73	6.88
GSE21653	GSM540284	BC	495	3	7.39	9.76	9.15	9.87	9.47	7.54
GSE21653	GSM540270	BC	496	3	7.19	9.80	9.39	9.80	8.90	7.54
GSE21653	GSM540177	BC	497	3	8.13	10.29	9.95	10.68	9.25	7.60
GSE21653	GSM540269	BC	498	3	8.27	9.75	9.71	9.26	9.37	7.22
GSE21653	GSM540116	BC	499	3	6.97	9.92	9.19	10.00	8.34	7.39
GSE21653	GSM540221	BC	500	3	8.77	9.89	10.46	11.08	8.92	7.62
GSE21653	GSM540332	BC	501	3	8.25	9.37	9.14	9.79	9.20	8.03
GSE21653	GSM540165	BC	502	3	7.89	9.25	10.10	8.86	9.10	7.26

GSE21653	GSM540168	BC	503	3	6.87	10.42	9.60	9.84	8.55	7.25
GSE21653	GSM540196	BC	504	3	7.35	10.09	9.54	9.65	8.35	7.27
GSE21653	GSM540253	BC	505	3	6.52	10.00	9.66	9.48	8.47	7.46
GSE21653	GSM540161	BC	506	3	6.89	10.63	9.81	10.17	8.96	7.17
GSE21653	GSM540278	BC	507	3	9.37	8.77	9.01	9.83	8.58	8.96
GSE21653	GSM540110	BC	508	3	7.43	10.00	9.29	7.97	9.17	6.44
GSE21653	GSM540109	BC	509	3	7.19	10.35	10.07	7.96	8.18	6.60
GSE21653	GSM540128	BC	510	3	8.95	8.89	8.95	9.50	9.25	7.71
GSE21653	GSM540120	BC	511	3	7.79	9.87	9.22	8.52	9.07	7.58
GSE21653	GSM540137	BC	512	3	7.01	11.06	9.26	8.49	8.03	6.19
GSE21653	GSM540343	BC	513	3	9.87	8.91	9.67	12.14	8.87	8.34
GSE21653	GSM540326	BC	514	3	5.64	8.71	9.00	9.26	8.89	5.43
GSE21653	GSM540224	BC	515	3	7.58	10.24	9.03	9.19	9.34	6.21
GSE21653	GSM540123	BC	516	3	7.48	9.32	8.77	9.07	9.53	7.27
GSE21653	GSM540298	BC	517	3	6.66	9.21	9.62	10.76	9.14	7.10
GSE21653	GSM540163	BC	518	3	5.39	10.44	9.61	8.44	9.03	6.00
GSE21653	GSM540114	BC	519	3	5.86	9.42	9.23	9.79	8.96	6.67
GSE21653	GSM540280	BC	520	3	7.42	9.56	9.24	9.76	8.64	7.02
GSE21653	GSM540228	BC	521	3	6.66	9.93	9.00	9.88	8.77	6.89
GSE21653	GSM540178	BC	522	3	5.81	10.00	9.94	8.15	8.70	5.29
GSE21653	GSM540246	BC	523	3	7.43	10.13	9.49	9.98	9.18	7.94
GSE21653	GSM540126	BC	524	3	6.60	9.64	9.11	8.20	9.17	7.38
GSE21653	GSM540363	BC	525	3	7.67	9.58	9.29	10.56	9.55	7.23
GSE21653	GSM540172	BC	526	3	7.27	9.67	9.50	9.76	9.37	7.61
GSE21653	GSM540122	BC	527	3	7.30	9.26	9.89	10.68	9.19	7.88
GSE21653	GSM540160	BC	528	3	7.09	8.79	9.03	9.55	9.57	7.62
GSE21653	GSM540309	BC	529	3	6.91	9.67	9.84	10.69	8.85	7.83
GSE21653	GSM540250	BC	530	3	7.21	9.26	9.12	10.17	9.21	7.67
GSE21653	GSM540257	BC	531	3	7.78	10.27	10.20	8.70	8.86	6.54
GSE21653	GSM540370	BC	532	3	7.52	10.11	9.56	10.66	9.08	7.67
GSE21653	GSM540213	BC	533	3	8.78	7.42	8.22	9.82	8.46	8.46
GSE21653	GSM540265	BC	534	3	8.35	7.08	7.93	10.77	9.59	8.45
GSE21653	GSM540111	BC	535	3	7.35	9.78	10.05	9.93	9.43	7.20
GSE21653	GSM540283	BC	536	3	7.67	9.26	10.12	9.91	9.07	8.68
GSE21653	GSM540216	BC	537	3	7.85	10.00	9.06	9.83	9.30	7.15
GSE21653	GSM540272	BC	538	3	7.21	10.33	11.29	9.13	9.06	7.01
GSE21653	GSM540268	BC	539	3	9.65	9.95	9.71	9.48	8.82	6.93
GSE21653	GSM540358	BC	540	3	9.55	9.78	9.17	10.00	8.82	6.57
GSE21653	GSM540297	BC	541	3	8.23	8.91	8.97	8.80	9.50	7.43
GSE21653	GSM540346	BC	542	3	6.13	10.37	10.08	7.81	9.00	5.98
GSE21653	GSM540174	BC	543	3	8.67	9.29	9.94	9.44	8.80	8.52
GSE21653	GSM540274	BC	544	3	7.33	10.09	9.60	9.55	9.10	7.64
GSE21653	GSM540236	BC	545	3	5.91	9.01	8.66	8.90	9.75	7.34
GSE21653	GSM540249	BC	546	3	8.55	9.44	10.06	8.88	8.60	8.23
GSE21653	GSM540348	BC	547	3	8.64	9.36	9.30	10.49	8.70	9.24
GSE21653	GSM540260	BC	548	3	7.91	9.73	9.97	8.95	9.03	8.29

GSE21653	GSM540304	BC	549	3	9.03	10.05	8.99	11.01	9.85	8.22
GSE21653	GSM540369	BC	550	3	8.83	9.55	9.92	10.50	9.98	8.39
GSE21653	GSM540373	BC	551	3	8.77	10.01	9.31	10.25	9.47	7.80
GSE21653	GSM540368	BC	552	3	8.34	9.68	9.91	10.09	10.17	8.79
GSE21653	GSM540256	BC	553	3	7.40	9.61	9.54	9.51	8.98	8.05
GSE21653	GSM540271	BC	554	3	8.34	8.97	9.07	7.87	9.24	7.97
GSE21653	GSM540352	BC	555	3	8.20	9.80	9.37	9.77	9.46	7.62
GSE21653	GSM540214	BC	556	3	8.35	9.22	9.29	10.17	10.25	9.07
GSE21653	GSM540261	BC	557	3	8.36	9.62	10.24	7.39	8.50	7.73
GSE21653	GSM540282	BC	558	3	8.71	9.28	10.48	9.58	9.96	7.91
GSE21653	GSM540285	BC	559	3	9.65	9.83	9.37	9.48	10.06	8.30
GSE21653	GSM540167	BC	560	3	7.48	9.72	9.70	10.25	9.48	6.04
GSE21653	GSM540357	BC	561	3	8.73	10.05	8.99	10.38	9.94	7.24
GSE21653	GSM540356	BC	562	3	9.34	10.02	9.68	9.42	9.93	7.56
GSE21653	GSM540281	BC	563	3	8.73	9.82	9.41	9.08	9.65	6.87
GSE21653	GSM540288	BC	564	3	8.84	10.02	9.32	9.82	10.48	8.77
GSE21653	GSM540206	BC	565	3	7.91	10.10	10.57	8.71	9.86	7.57
GSE21653	GSM540353	BC	566	3	8.53	9.08	9.71	10.11	9.83	7.28
GSE21653	GSM540248	BC	567	3	8.27	8.58	9.46	10.54	8.68	7.94
GSE21653	GSM540264	BC	568	3	9.50	10.31	10.04	9.30	8.60	7.34
GSE21653	GSM540254	BC	569	3	8.52	9.89	9.41	9.59	9.18	7.35
GSE21653	GSM540291	BC	570	3	8.78	9.20	10.04	10.55	8.55	7.03
GSE21653	GSM540125	BC	571	3	7.50	10.68	9.87	9.75	9.75	7.42
GSE21653	GSM540361	BC	572	3	6.73	9.85	9.98	9.92	9.29	7.48
GSE21653	GSM540360	BC	573	3	10.27	10.48	9.49	8.99	9.65	8.14
GSE21653	GSM540132	BC	574	3	8.05	9.87	10.16	8.83	9.47	9.13
GSE21653	GSM540351	BC	575	3	7.73	9.75	10.02	10.45	9.21	8.34
GSE21653	GSM540292	BC	576	3	8.89	9.94	10.54	7.55	9.02	7.77
GSE21653	GSM540362	BC	577	3	8.97	10.11	9.99	9.75	9.69	7.91
GSE21653	GSM540146	BC	578	3	9.07	10.29	9.99	7.73	8.60	7.08
GSE21653	GSM540345	BC	579	3	8.99	9.86	9.82	10.69	9.43	8.44
GSE21653	GSM540294	BC	580	3	10.34	10.31	9.96	10.93	9.41	8.07
GSE21653	GSM540365	BC	581	3	7.63	10.15	9.64	9.91	9.43	7.24
GSE21653	GSM540310	BC	582	3	8.44	9.67	9.42	10.28	9.72	8.42
GSE21653	GSM540186	BC	583	3	7.75	11.01	9.23	8.62	9.26	7.11
GSE21653	GSM540176	BC	584	3	9.19	10.43	10.06	10.31	8.43	7.71
GSE21653	GSM540127	BC	585	3	8.59	9.66	9.79	9.32	9.39	7.61
GSE21653	GSM540325	BC	586	3	7.52	10.25	9.71	10.50	7.60	8.03
GSE21653	GSM540302	BC	587	3	10.33	10.54	9.90	10.51	8.97	8.58
GSE21653	GSM540238	BC	588	3	7.28	9.53	9.74	9.06	9.15	8.37
GSE21653	GSM540124	BC	589	3	8.47	9.69	9.97	9.29	8.68	9.11
GSE21653	GSM540355	BC	590	3	7.61	10.33	9.46	9.03	9.51	7.89
GSE21653	GSM540366	BC	591	3	8.76	9.36	9.71	11.00	9.03	8.36
GSE21653	GSM540121	BC	592	3	8.14	10.78	10.03	9.03	9.03	7.11
GSE21653	GSM540276	BC	593	3	8.02	9.77	9.73	9.58	9.45	8.01
GSE21653	GSM540164	BC	594	3	6.98	9.92	9.99	8.44	8.60	6.29

GSE21653	GSM540371	BC	595	3	8.72	9.75	10.21	9.83	9.57	8.43
GSE21653	GSM540130	BC	596	3	6.85	8.98	10.16	8.64	9.24	7.18
GSE21653	GSM540181	BC	597	3	8.62	9.98	10.19	8.58	8.84	7.99
GSE21653	GSM540209	BC	598	3	8.12	10.20	10.60	9.19	8.55	7.55
GSE21653	GSM540119	BC	599	3	8.19	11.01	9.83	8.56	8.94	7.79
GSE21653	GSM540372	BC	600	3	8.14	10.18	10.90	10.09	8.95	7.64
GSE21653	GSM540138	BC	601	3	8.46	9.54	9.99	8.13	9.49	8.86
GSE21653	GSM540147	BC	602	3	7.49	10.66	9.65	7.81	9.06	7.23
GSE21653	GSM540195	BC	603	3	7.89	9.75	9.78	7.59	9.70	6.66
GSE21653	GSM540219	BC	604	3	8.80	10.06	9.79	9.00	8.96	7.85
GSE21653	GSM540215	BC	605	3	8.13	10.15	10.08	8.53	9.46	8.83
GSE21653	GSM540140	BC	606	3	9.15	9.65	10.15	7.72	8.89	7.62
GSE21653	GSM540307	BC	607	3	9.70	10.56	9.68	10.18	9.52	8.54
GSE21653	GSM540273	BC	608	3	8.15	10.74	9.85	10.97	9.32	8.12
GSE31448	GSM781503	BC	609	3	8.76	9.89	9.60	10.53	8.53	7.01
GSE31448	GSM781544	BC	610	3	8.22	10.62	9.85	9.76	9.17	8.01
GSE31448	GSM781351	BC	611	3	6.98	11.08	9.28	8.46	8.00	6.17
GSE31448	GSM781514	BC	612	3	10.31	9.81	8.95	10.49	8.96	8.56
GSE31448	GSM781346	BC	613	3	8.02	11.00	10.03	8.81	9.45	9.11
GSE31448	GSM781464	BC	614	3	7.18	10.27	9.45	10.15	9.19	7.65
GSE31448	GSM781338	BC	615	3	8.45	9.60	10.09	9.28	8.66	9.09
GSE31448	GSM781563	BC	616	3	7.72	10.56	10.23	10.43	9.19	8.33
GSE31448	GSM781484	BC	617	3	7.17	9.99	10.64	9.78	8.89	7.52
GSE31448	GSM781554	BC	618	3	8.62	10.61	9.14	9.89	8.98	7.81
GSE31448	GSM781575	BC	619	3	7.65	9.48	8.81	10.55	9.54	7.21
GSE31448	GSM781463	BC	620	3	8.52	9.90	10.47	8.85	8.58	8.20
GSE31448	GSM781378	BC	621	3	6.95	10.65	9.83	8.42	8.58	6.27
GSE31448	GSM781409	BC	622	3	7.86	9.69	9.29	7.57	9.67	6.63
GSE31448	GSM781567	BC	623	3	7.59	9.60	9.67	9.02	9.50	7.87
GSE31448	GSM781483	BC	624	3	8.26	9.39	9.16	9.24	9.35	7.20
GSE31448	GSM781504	BC	625	3	8.87	10.42	10.10	7.52	9.00	7.75
GSE31448	GSM781580	BC	626	3	8.32	10.13	10.46	10.08	10.15	8.77
GSE31448	GSM781498	BC	627	3	9.64	8.95	9.55	9.47	10.05	8.29
GSE31448	GSM781470	BC	628	3	7.38	9.96	8.91	9.50	8.97	8.03
GSE31448	GSM781568	BC	629	3	9.32	9.89	9.24	9.39	9.91	7.54
GSE31448	GSM781574	BC	630	3	8.95	9.81	9.65	9.74	9.67	7.89
GSE31448	GSM781462	BC	631	3	8.24	10.09	9.50	10.52	8.66	7.92
GSE31448	GSM781506	BC	632	3	10.32	10.18	10.05	10.91	9.39	8.04
GSE31448	GSM781555	BC	633	3	9.86	9.94	9.21	12.13	8.85	8.34
GSE31448	GSM781429	BC	634	3	8.11	9.75	9.92	8.51	9.44	8.81
GSE31448	GSM781485	BC	635	3	8.32	9.40	9.90	7.85	9.22	7.95
GSE31448	GSM781395	BC	636	3	8.61	9.41	10.09	8.56	8.82	7.97
GSE31448	GSM781495	BC	637	3	8.69	10.32	9.81	9.56	9.94	7.89
GSE31448	GSM781423	BC	638	3	8.10	10.21	10.37	9.17	8.54	7.52
GSE31448	GSM781352	BC	639	3	8.44	10.20	10.61	8.11	9.47	8.85
GSE31448	GSM781496	BC	640	3	7.66	9.43	10.01	9.89	9.06	8.66

GSE31448	GSM781356	BC	641	3	7.64	10.87	9.77	9.65	9.63	7.93
GSE31448	GSM781572	BC	642	3	10.26	10.46	10.26	8.97	9.64	8.12
GSE31448	GSM781560	BC	643	3	8.62	9.90	9.93	10.48	8.69	9.23
GSE31448	GSM781339	BC	644	3	7.48	9.00	9.57	9.73	9.73	7.40
GSE31448	GSM781428	BC	645	3	8.34	9.75	9.74	10.15	10.24	9.06
GSE31448	GSM781460	BC	646	3	7.42	9.88	9.54	9.96	9.16	7.92
GSE31448	GSM781474	BC	647	3	7.89	9.87	10.17	8.92	9.01	8.26
GSE31448	GSM781340	BC	648	3	6.57	10.28	10.14	8.18	9.16	7.35
GSE31448	GSM781374	BC	649	3	7.07	8.94	9.61	9.54	9.55	7.61
GSE31448	GSM781391	BC	650	3	8.12	9.47	11.01	10.67	9.23	7.58
GSE31448	GSM781341	BC	651	3	8.58	9.34	9.79	9.30	9.38	7.59
GSE31448	GSM781522	BC	652	3	8.42	10.06	9.26	10.27	9.70	8.41
GSE31448	GSM781521	BC	653	3	6.88	9.77	9.80	10.67	8.83	7.80
GSE31448	GSM781420	BC	654	3	7.89	9.54	10.16	8.69	9.84	7.55
GSE31448	GSM781450	BC	655	3	5.86	10.78	11.23	8.88	9.73	7.32
GSE31448	GSM781390	BC	656	3	9.17	9.40	9.71	10.29	8.41	7.69
GSE31448	GSM781400	BC	657	3	7.73	9.82	10.19	8.59	9.24	7.09
GSE31448	GSM781490	BC	658	3	8.00	9.77	9.74	9.56	9.43	7.99
GSE31448	GSM781565	BC	659	3	8.51	10.65	9.93	10.09	9.82	7.26
GSE31448	GSM781475	BC	660	3	8.33	9.01	10.17	7.37	8.48	7.70
GSE31448	GSM781392	BC	661	3	5.78	8.97	9.80	8.13	8.69	5.25
GSE31448	GSM781519	BC	662	3	9.68	9.81	10.01	10.17	9.50	8.52
GSE31448	GSM781523	BC	663	3	7.83	9.62	10.23	9.91	8.50	8.12
GSE31448	GSM781538	BC	664	3	5.61	9.96	9.88	9.24	8.87	5.43
GSE31448	GSM781362	BC	665	3	7.24	9.56	10.24	11.22	8.96	6.38
GSE31448	GSM781325	BC	666	3	7.32	9.47	10.61	9.91	9.41	7.18
GSE31448	GSM781379	BC	667	3	7.88	11.09	9.82	8.84	9.09	7.24
GSE31448	GSM781509	BC	668	3	8.20	9.93	10.27	8.78	9.48	7.41
GSE31448	GSM781377	BC	669	3	5.36	9.93	9.20	8.42	9.02	5.98
GSE31448	GSM781342	BC	670	3	8.94	9.61	10.16	9.49	9.23	7.69
GSE31448	GSM781381	BC	671	3	7.46	9.78	10.00	10.23	9.47	6.02
GSE31448	GSM781410	BC	672	3	7.33	10.05	9.61	9.63	8.34	7.25
GSE31448	GSM781452	BC	673	3	7.25	9.87	10.37	9.03	9.12	8.34
GSE31448	GSM781435	BC	674	3	8.75	10.13	9.69	11.06	8.89	7.60
GSE31448	GSM781335	BC	675	3	8.13	9.76	9.97	9.01	9.01	7.09
GSE31448	GSM781330	BC	676	3	6.94	9.16	9.34	9.98	8.32	7.38
GSE31448	GSM781427	BC	677	3	8.77	10.19	10.57	9.81	8.44	8.44
GSE31448	GSM781569	BC	678	3	8.71	10.53	9.95	10.36	9.92	7.22
GSE31448	GSM781573	BC	679	3	6.71	11.22	9.08	9.91	9.27	7.45
GSE31448	GSM781471	BC	680	3	7.76	9.94	9.49	8.68	8.84	6.51
GSE31448	GSM781558	BC	681	3	6.11	9.91	10.66	7.79	8.98	5.95
GSE31448	GSM781388	BC	682	3	8.65	10.34	9.97	9.42	8.79	8.50
GSE31448	GSM781328	BC	683	3	5.83	9.77	11.03	9.77	8.93	6.64
GSE31448	GSM781479	BC	684	3	8.33	9.52	10.11	10.75	9.58	8.43
GSE31448	GSM781482	BC	685	3	9.63	10.71	9.94	9.46	8.80	6.91
GSE31448	GSM781570	BC	686	3	9.54	9.62	9.72	9.98	8.80	6.54

GSE31448	GSM781584	BC	687	3	8.13	10.58	9.96	10.07	8.93	7.63
GSE31448	GSM781491	BC	688	3	7.47	10.78	9.47	9.58	8.72	6.86
GSE31448	GSM781360	BC	689	3	9.06	8.94	9.91	7.72	8.58	7.07
GSE31448	GSM781582	BC	690	3	7.50	10.93	9.90	10.65	9.06	7.65
GSE31448	GSM781488	BC	691	3	7.31	8.76	9.83	9.54	9.08	7.62
GSE31448	GSM781585	BC	692	3	8.76	9.33	10.58	10.24	9.45	7.78
GSE31448	GSM781442	BC	693	3	6.64	10.33	10.34	9.87	8.76	6.87
GSE31448	GSM781515	BC	694	3	8.76	9.12	10.15	8.69	8.64	7.73
GSE31448	GSM781577	BC	695	3	7.61	9.71	10.51	9.90	9.42	7.22
GSE31448	GSM781336	BC	696	3	7.29	10.55	10.59	10.67	9.17	7.87
GSE31448	GSM781578	BC	697	3	8.75	10.02	9.90	10.98	9.01	8.34
GSE31448	GSM781467	BC	698	3	6.49	10.48	10.67	9.46	8.45	7.43
GSE31448	GSM781557	BC	699	3	8.98	10.30	9.97	10.68	9.42	8.43
GSE31448	GSM781516	BC	700	3	9.01	11.12	9.87	10.99	9.84	8.20
GSE31448	GSM781361	BC	701	3	7.47	9.97	9.41	7.79	9.03	7.20
GSE31448	GSM781375	BC	702	3	6.88	10.92	9.38	10.16	8.95	7.16
GSE31448	GSM781497	BC	703	3	7.37	10.34	9.69	9.85	9.45	7.52
GSE31448	GSM781468	BC	704	3	8.50	9.92	9.68	9.57	9.17	7.33
GSE31448	GSM781354	BC	705	3	9.14	9.56	9.74	7.70	8.88	7.60
GSE31448	GSM781494	BC	706	3	7.39	10.92	9.83	9.74	8.62	7.00
GSE31448	GSM781581	BC	707	3	8.82	10.10	10.38	10.49	9.97	8.38
GSE31448	GSM781438	BC	708	3	7.56	9.46	9.68	9.17	9.33	6.17
GSE31448	GSM781486	BC	709	3	7.18	9.75	9.66	9.11	9.04	6.99
GSE31448	GSM781564	BC	710	3	8.18	9.69	9.59	9.75	9.44	7.60
GSE31448	GSM781478	BC	711	3	9.48	9.47	9.66	9.28	8.58	7.31
GSE31448	GSM781487	BC	712	3	8.13	9.75	9.35	10.95	9.30	8.10
GSE31448	GSM781583	BC	713	3	8.71	10.17	8.58	9.82	9.56	8.42
GSE31448	GSM781333	BC	714	3	8.16	9.58	11.01	8.54	8.92	7.76
GSE31448	GSM781334	BC	715	3	7.77	10.47	9.66	8.50	9.06	7.55
GSE31448	GSM781396	BC	716	3	8.02	10.12	10.62	9.58	8.46	7.04
GSE31448	GSM781492	BC	717	3	9.35	9.17	9.63	9.81	8.56	8.94
GSE31448	GSM781430	BC	718	3	7.83	9.87	10.25	9.81	9.29	7.13
GSE31448	GSM781344	BC	719	3	6.82	10.24	9.74	8.62	9.21	7.15
GSE31448	GSM781382	BC	720	3	6.85	9.88	10.37	9.82	8.53	7.23
GSE31448	GSM781386	BC	721	3	7.24	9.37	9.43	9.74	9.36	7.59
GSE31448	GSM781510	BC	722	3	6.64	9.41	10.18	10.75	9.13	7.09
GSE31448	GSM781337	BC	723	3	7.45	9.92	9.83	9.05	9.51	7.25
GSE31448	GSM781433	BC	724	3	8.78	9.82	10.35	8.99	8.95	7.84
GSE31448	GSM781537	BC	725	3	7.50	9.22	9.52	10.48	7.58	8.01

* BC = Breast Cancer, TS = Tissue, N = Number of samples, ST = Breast cancer Subtype

Table S4. Gene expression of MCM10, RPA, ATR and CHEK1 in breast cancer cell lines

Dataset ID	Sample ID	Cell line	Tissue	Numbers	MCM10
GSE63354	GSM1546532	MCF10A	Breast-Normal	1	9.572
GSE63354	GSM1546533	MCF10A	Breast-Normal	2	9.285
GSE63354	GSM1546534	MCF10A	Breast-Normal	3	9.129
GSE63354	GSM1546535	MCF10A	Breast-Normal	4	8.741
GSE63354	GSM1546536	MCF10A	Breast-Normal	5	7.983
GSE63354	GSM1546537	MCF10A	Breast-Normal	6	7.728
GSE67301	GSM1644038	MCF10A	Breast-Normal	7	5.459
GSE67301	GSM1644039	MCF10A	Breast-Normal	8	5.615
GSE10890	GSM275995	MCF10A	Breast-Normal	9	6.977
GSE10890	GSM275996	MCF10A	Breast-Normal	10	9.172
GSE40059	GSM984504	MCF10A	Breast-Normal	11	7.392
GSE20713	GSM553873	MCF10A	Breast-Normal	12	7.607
GSE52032	GSM1257857	MCF10A	Breast-Normal	13	6.322
GSE32474	GSM803625	MDA231	Breast-Cancer	14	9.087
GSE32474	GSM803684	MDA231	Breast-Cancer	15	9.170
GSE32474	GSM803743	MDA231	Breast-Cancer	16	9.003
GSE10890	GSM276037	MDA231	Breast-Cancer	17	10.015
GSE10890	GSM276038	MDA231	Breast-Cancer	18	9.957
GSE10890	GSM276039	MDA231	Breast-Cancer	19	9.699
GSE10890	GSM275993	MDA231	Breast-Cancer	20	9.238
GSE27515	GSM679722	MDA231	Breast-Cancer	21	10.066
GSE27515	GSM679723	MDA231	Breast-Cancer	22	9.845
GSE27515	GSM679724	MDA231	Breast-Cancer	23	9.753
GSE10021	GSM253208	MDA231	Breast-Cancer	24	7.592
GSE10843	GSM274653	MDA231	Breast-Cancer	25	9.238
GSE12777	GSM320604	MDA231	Breast-Cancer	26	9.238
GSE25487	GSM626476	MDA231	Breast-Cancer	27	9.803
GSE25487	GSM626477	MDA231	Breast-Cancer	28	9.740
GSE25487	GSM626478	MDA231	Breast-Cancer	29	9.823
GSE25487	GSM626479	MDA231	Breast-Cancer	30	9.531
GSE25487	GSM626480	MDA231	Breast-Cancer	31	9.371
GSE25487	GSM626481	MDA231	Breast-Cancer	32	9.371
GSE25487	GSM626482	MDA231	Breast-Cancer	33	9.111
GSE25487	GSM626483	MDA231	Breast-Cancer	34	8.852
GSE25487	GSM626484	MDA231	Breast-Cancer	35	9.944
GSE25487	GSM626485	MDA231	Breast-Cancer	36	9.916
GSE34211	GSM844594	MDA231	Breast-Cancer	37	9.422
GSE34211	GSM844595	MDA231	Breast-Cancer	38	9.604
GSE40464	GSM994556	MDA231	Breast-Cancer	39	9.889
GSE40464	GSM994557	MDA231	Breast-Cancer	40	8.589
GSE41445	GSM1017490	MDA231	Breast-Cancer	41	9.315
GSE41445	GSM1017491	MDA231	Breast-Cancer	42	9.017
GSE41445	GSM1017492	MDA231	Breast-Cancer	43	9.264

GSE45827	GSM1116162	MDA231	Breast-Cancer	44	8.562
GSE47379	GSM1148312	MDA231	Breast-Cancer	45	9.793
GSE47379	GSM1148313	MDA231	Breast-Cancer	46	9.790
GSE47379	GSM1148314	MDA231	Breast-Cancer	47	10.003
GSE50832	GSM1230134	MDA231	Breast-Cancer	48	9.984
GSE50832	GSM1230135	MDA231	Breast-Cancer	49	9.786
GSE50832	GSM1230136	MDA231	Breast-Cancer	50	9.901
GSE60125	GSM1465323	MDA231	Breast-Cancer	51	8.066
GSE60125	GSM1465324	MDA231	Breast-Cancer	52	8.155
GSE60125	GSM1465325	MDA231	Breast-Cancer	53	8.170
GSE65216	GSM1589153	MDA231	Breast-Cancer	54	8.562
GSE32670	GSM810964	MDA231	Breast-Cancer	55	8.714
GSE32670	GSM810965	MDA231	Breast-Cancer	56	10.212
GSE33167	GSM820814	MDA231	Breast-Cancer	57	9.160
GSE33167	GSM820815	MDA231	Breast-Cancer	58	9.187
GSE33167	GSM820816	MDA231	Breast-Cancer	59	9.335
GSE36441	GSM893545	MDA231	Breast-Cancer	60	9.707
GSE36441	GSM893546	MDA231	Breast-Cancer	61	9.545
GSE36441	GSM893547	MDA231	Breast-Cancer	62	9.660
GSE36953	GSM907019	MDA231	Breast-Cancer	63	9.747
GSE36953	GSM907020	MDA231	Breast-Cancer	64	8.071
GSE39042	GSM954535	MDA231	Breast-Cancer	65	9.886
GSE39042	GSM954541	MDA231	Breast-Cancer	66	9.828
GSE39042	GSM954547	MDA231	Breast-Cancer	67	8.271
GSE40059	GSM984500	MDA231	Breast-Cancer	68	8.397
GSE30350	GSM317505	MCF7	Breast-Cancer	69	9.264
GSE3156	GSM70659	MCF7	Breast-Cancer	70	9.101
gsk	gsk_446	MCF7	Breast-Cancer	71	9.317
gsk	gsk_447	MCF7	Breast-Cancer	72	9.207
gsk	gsk_448	MCF7	Breast-Cancer	73	9.257
GSE41445	GSM1017487	MCF7	Breast-Cancer	74	9.388
GSE41445	GSM1017488	MCF7	Breast-Cancer	75	9.313
GSE41445	GSM1017489	MCF7	Breast-Cancer	76	9.478
GSE43657	GSM1067677	MCF7	Breast-Cancer	77	8.765
GSE43657	GSM1067678	MCF7	Breast-Cancer	78	9.066
GSE50820	GSM1230317	MCF7	Breast-Cancer	79	8.087
GSE51238	GSM1240794	MCF7	Breast-Cancer	80	10.014
GSE51238	GSM1240795	MCF7	Breast-Cancer	81	9.581
GSE51238	GSM1240796	MCF7	Breast-Cancer	82	9.893
GSE10021	GSM253207	MCF7	Breast-Cancer	83	7.741
GSE10843	GSM274640	MCF7	Breast-Cancer	84	9.451
GSE10890	GSM275978	MCF7	Breast-Cancer	85	9.451
GSE10890	GSM276046	MCF7	Breast-Cancer	86	9.496
GSE10890	GSM276047	MCF7	Breast-Cancer	87	9.635
GSE10890	GSM276048	MCF7	Breast-Cancer	88	9.693
GSE12777	GSM320602	MCF7	Breast-Cancer	89	9.451

GSE17889	GSM446723	MCF7	Breast-Cancer	90	8.124
GSE17889	GSM446724	MCF7	Breast-Cancer	91	8.017
GSE24547	GSM605093	MCF7	Breast-Cancer	92	9.074
GSE24547	GSM605094	MCF7	Breast-Cancer	93	9.190
GSE32474	GSM803623	MCF7	Breast-Cancer	94	7.864
GSE32474	GSM803682	MCF7	Breast-Cancer	95	8.633
GSE32474	GSM803741	MCF7	Breast-Cancer	96	7.508
GSE34211	GSM844586	MCF7	Breast-Cancer	97	9.137
GSE34211	GSM844587	MCF7	Breast-Cancer	98	9.379
GSE40059	GSM984498	MCF7	Breast-Cancer	99	8.804
GSE40968	GSM1006127	MCF7	Breast-Cancer	100	9.592
GSE40968	GSM1006128	MCF7	Breast-Cancer	101	9.464
GSE40968	GSM1006129	MCF7	Breast-Cancer	102	9.644
GSE41635	GSM1020604	MCF7	Breast-Cancer	103	7.607
GSE41635	GSM1020605	MCF7	Breast-Cancer	104	7.435
GSE50832	GSM1230054	MCF7	Breast-Cancer	105	10.201
GSE50832	GSM1230055	MCF7	Breast-Cancer	106	10.194
GSE50832	GSM1230056	MCF7	Breast-Cancer	107	10.295
GSE76540	GSM2027638	MCF7	Breast-Cancer	108	9.869
GSE76540	GSM2027639	MCF7	Breast-Cancer	109	9.815
GSE76540	GSM2027640	MCF7	Breast-Cancer	110	9.527
GSE15749	GSM394765	MCF7	Breast-Cancer	111	8.435
GSE15749	GSM394766	MCF7	Breast-Cancer	112	8.439
GSE29672	GSM735885	MCF7	Breast-Cancer	113	9.676
GSE29672	GSM735886	MCF7	Breast-Cancer	114	9.602
GSE29672	GSM735887	MCF7	Breast-Cancer	115	9.553
GSE32670	GSM810956	MCF7	Breast-Cancer	116	9.243
GSE32670	GSM810957	MCF7	Breast-Cancer	117	8.655
GSE41445	GSM1017511	T47D	Breast-Cancer	118	9.412
GSE41445	GSM1017512	T47D	Breast-Cancer	119	9.514
GSE41445	GSM1017513	T47D	Breast-Cancer	120	9.414
GSE50832	GSM1230045	T47D	Breast-Cancer	121	9.812
GSE50832	GSM1230046	T47D	Breast-Cancer	122	9.577
GSE50832	GSM1230047	T47D	Breast-Cancer	123	9.447
GSE10843	GSM274651	T47D	Breast-Cancer	124	9.516
GSE10843	GSM274652	T47D	Breast-Cancer	125	9.674
GSE10843	GSM274664	T47D	Breast-Cancer	126	9.317
GSE10890	GSM275991	T47D	Breast-Cancer	127	9.516
GSE10890	GSM275992	T47D	Breast-Cancer	128	9.674
GSE12777	GSM320612	T47D	Breast-Cancer	129	9.317
GSE32474	GSM803673	T47D	Breast-Cancer	130	6.555
GSE32474	GSM803731	T47D	Breast-Cancer	131	6.000
GSE32474	GSM803788	T47D	Breast-Cancer	132	6.267
GSE32670	GSM810945	T47D	Breast-Cancer	133	9.109
GSE32670	GSM810946	T47D	Breast-Cancer	134	9.170
GSE34211	GSM844714	T47D	Breast-Cancer	135	9.340

GSE34211	GSM844715	T47D	Breast-Cancer	136	9.451
GSE34211	GSM844716	T47D	Breast-Cancer	137	9.167
GSE40059	GSM984496	T47D	Breast-Cancer	138	9.127
GSE50832	GSM1230081	SKBR3	Breast-Cancer	139	9.379
GSE50832	GSM1230082	SKBR3	Breast-Cancer	140	9.394
GSE50832	GSM1230083	SKBR3	Breast-Cancer	141	9.255
GSE72362	GSM1861028	SKBR3	Breast-Cancer	142	10.027
GSE72362	GSM1861029	SKBR3	Breast-Cancer	143	9.664
GSE72362	GSM1861030	SKBR3	Breast-Cancer	144	9.103
GSE10843	GSM274644	SKBR3	Breast-Cancer	145	8.928
GSE10843	GSM274663	SKBR3	Breast-Cancer	146	8.867
GSE10890	GSM275983	SKBR3	Breast-Cancer	147	8.928
GSE12777	GSM320611	SKBR3	Breast-Cancer	148	8.867
GSE32670	GSM810975	SKBR3	Breast-Cancer	149	8.945
GSE34211	GSM844686	SKBR3	Breast-Cancer	150	8.880
GSE34211	GSM844687	SKBR3	Breast-Cancer	151	9.288
GSE40059	GSM984499	SKBR3	Breast-Cancer	152	9.335

Table S5. Gene expression of MCM10 in breast cancer patient's samples having different degree of BC malignancy based on molecular subtypes

Dataset_ID	Sample_ID	Subtype	MCM 10 gene_expression
GSE17907	GSM447207	Luminal_A	6.06608919
GSE17907	GSM447208	Luminal_A	7.357552005
GSE17907	GSM447218	Luminal_A	8.224001674
GSE17907	GSM447227	Luminal_A	6.491853096
GSE17907	GSM447234	Luminal_A	7.17990909
GSE17907	GSM447243	Luminal_A	7.118941073
GSE17907	GSM447244	Luminal_A	5.523561956
GSE17907	GSM447245	Luminal_A	7.900866808
GSE20713	GSM519735	Luminal_A	5.087462841
GSE20713	GSM519736	Luminal_A	4.523561956
GSE20713	GSM519739	Luminal_A	7.321928095
GSE20713	GSM519744	Luminal_A	6.087462841
GSE20713	GSM519758	Luminal_A	5.977279923
GSE20713	GSM519761	Luminal_A	5.554588852
GSE20713	GSM519762	Luminal_A	4.95419631
GSE20713	GSM519763	Luminal_A	6.599912842
GSE20713	GSM519765	Luminal_A	4.807354922
GSE20713	GSM519770	Luminal_A	7.375039431
GSE20713	GSM519780	Luminal_A	6.129283017
GSE20713	GSM519788	Luminal_A	5.129283017
GSE20713	GSM519789	Luminal_A	6.087462841
GSE20713	GSM519790	Luminal_A	6.14974712
GSE20713	GSM519791	Luminal_A	5.807354922
GSE20713	GSM519793	Luminal_A	6.321928095
GSE20713	GSM519795	Luminal_A	5.247927513
GSE20713	GSM519796	Luminal_A	6.426264755
GSE20713	GSM519798	Luminal_A	5.584962501
GSE20713	GSM519804	Luminal_A	6.189824559
GSE20713	GSM519807	Luminal_A	5.044394119
GSE20713	GSM519809	Luminal_A	3.807354922
GSE20713	GSM519810	Luminal_A	5.321928095
GSE21653	GSM540315	Luminal_A	5.523561956
GSE21653	GSM540305	Luminal_A	6.14974712
GSE21653	GSM540313	Luminal_A	6.658211483
GSE21653	GSM540321	Luminal_A	5.129283017
GSE21653	GSM540239	Luminal_A	7
GSE21653	GSM540227	Luminal_A	5.754887502
GSE21653	GSM540144	Luminal_A	5.087462841
GSE21653	GSM540163	Luminal_A	5.392317423
GSE21653	GSM540229	Luminal_A	7.375039431
GSE21653	GSM540240	Luminal_A	5.672425342
GSE21653	GSM540233	Luminal_A	5.129283017
GSE21653	GSM540225	Luminal_A	6.672425342

GSE21653	GSM540330	Luminal_A	4.95419631
GSE21653	GSM540197	Luminal_A	6.247927513
GSE21653	GSM540212	Luminal_A	5.672425342
GSE21653	GSM540171	Luminal_A	7.366322214
GSE21653	GSM540198	Luminal_A	7.076815597
GSE21653	GSM540179	Luminal_A	6.64385619
GSE21653	GSM540322	Luminal_A	5.491853096
GSE21653	GSM540222	Luminal_A	6.321928095
GSE21653	GSM540241	Luminal_A	5.857980995
GSE21653	GSM540337	Luminal_A	4.857980995
GSE21653	GSM540192	Luminal_A	6.614709844
GSE21653	GSM540203	Luminal_A	5.285402219
GSE21653	GSM540145	Luminal_A	5.95419631
GSE21653	GSM540114	Luminal_A	5.857980995
GSE21653	GSM540318	Luminal_A	5.491853096
GSE21653	GSM540317	Luminal_A	5
GSE21653	GSM540232	Luminal_A	5.781359714
GSE21653	GSM540193	Luminal_A	6.108524457
GSE21653	GSM540258	Luminal_A	6.14974712
GSE21653	GSM540169	Luminal_A	4.95419631
GSE21653	GSM540153	Luminal_A	6.044394119
GSE21653	GSM540202	Luminal_A	5.584962501
GSE21653	GSM540230	Luminal_A	7.033423002
GSE21653	GSM540334	Luminal_A	5.321928095
GSE21653	GSM540279	Luminal_A	6.06608919
GSE21653	GSM540275	Luminal_A	6.50779464
GSE21653	GSM540280	Luminal_A	7.417852515
GSE21653	GSM540220	Luminal_A	5.807354922
GSE21653	GSM540185	Luminal_A	7.312882955
GSE21653	GSM540262	Luminal_A	7.055282436
GSE21653	GSM540243	Luminal_A	6.658211483
GSE21653	GSM540228	Luminal_A	6.658211483
GSE21653	GSM540340	Luminal_A	6.906890596
GSE21653	GSM540247	Luminal_A	6.714245518
GSE21653	GSM540286	Luminal_A	6.108524457
GSE21653	GSM540178	Luminal_A	5.807354922
GSE21653	GSM540344	Luminal_A	4.906890596
GSE21653	GSM540246	Luminal_A	7.434628228
GSE21653	GSM540287	Luminal_A	7.658211483
GSE21653	GSM540199	Luminal_A	8.243173983
GSE21653	GSM540328	Luminal_A	4.584962501
GSE21653	GSM540296	Luminal_A	6.906890596
GSE21653	GSM540338	Luminal_A	6.247927513
GSE21653	GSM540126	Luminal_A	6.599912842
GSE21653	GSM540363	Luminal_A	7.665335917
GSE21653	GSM540205	Luminal_A	5.087462841

GSE21653	GSM540350	Luminal_A	6.426264755
GSE21653	GSM540112	Luminal_A	7.209453366
GSE21653	GSM540329	Luminal_A	6
GSE21653	GSM540172	Luminal_A	7.266786541
GSE21653	GSM540347	Luminal_A	7.276124405
GSE21653	GSM540207	Luminal_A	7.515699838
GSE21653	GSM540335	Luminal_A	5.426264755
GSE21653	GSM540324	Luminal_A	6.044394119
GSE21653	GSM540191	Luminal_A	6.50779464
GSE21653	GSM540349	Luminal_A	8.060695932
GSE21653	GSM540134	Luminal_A	7.285402219
GSE21653	GSM540135	Luminal_A	5.64385619
GSE21653	GSM540129	Luminal_A	7.409390936
GSE21653	GSM540133	Luminal_A	6.044394119
GSE21653	GSM540200	Luminal_A	7.06608919
GSE21653	GSM540122	Luminal_A	7.303780748
GSE21653	GSM540150	Luminal_A	5.857980995
GSE21653	GSM540160	Luminal_A	7.087462841
GSE21653	GSM540173	Luminal_A	6.988684687
GSE21653	GSM540175	Luminal_A	5.882643049
GSE21653	GSM540189	Luminal_A	5.64385619
GSE21653	GSM540190	Luminal_A	5.614709844
GSE21653	GSM540188	Luminal_A	5.807354922
GSE21653	GSM540194	Luminal_A	4.64385619
GSE21653	GSM540204	Luminal_A	5.129283017
GSE21653	GSM540136	Luminal_A	4.392317423
GSE21653	GSM540223	Luminal_A	5.357552005
GSE21653	GSM540210	Luminal_A	6.794415866
GSE21653	GSM540234	Luminal_A	6.894817763
GSE21653	GSM540211	Luminal_A	5.169925001
GSE21653	GSM540170	Luminal_A	6.285402219
GSE21653	GSM540112	Luminal_A	7.199672345
GSE21653	GSM540114	Luminal_A	5.832890014
GSE21653	GSM540122	Luminal_A	7.285402219
GSE21653	GSM540126	Luminal_A	6.569855608
GSE21653	GSM540129	Luminal_A	7.383704292
GSE21653	GSM540133	Luminal_A	6.022367813
GSE21653	GSM540134	Luminal_A	7.266786541
GSE21653	GSM540135	Luminal_A	5.614709844
GSE21653	GSM540136	Luminal_A	4.321928095
GSE21653	GSM540144	Luminal_A	5.044394119
GSE21653	GSM540145	Luminal_A	5.930737338
GSE21653	GSM540150	Luminal_A	5.832890014
GSE21653	GSM540153	Luminal_A	6.022367813
GSE21653	GSM540160	Luminal_A	7.06608919
GSE21653	GSM540163	Luminal_A	5.357552005

GSE21653	GSM540169	Luminal_A	4.906890596
GSE21653	GSM540170	Luminal_A	6.266786541
GSE21653	GSM540171	Luminal_A	7.348728154
GSE21653	GSM540172	Luminal_A	7.238404739
GSE21653	GSM540173	Luminal_A	6.977279923
GSE21653	GSM540175	Luminal_A	5.857980995
GSE21653	GSM540178	Luminal_A	5.781359714
GSE21653	GSM540179	Luminal_A	6.614709844
GSE21653	GSM540185	Luminal_A	7.294620749
GSE21653	GSM540188	Luminal_A	5.781359714
GSE21653	GSM540189	Luminal_A	5.614709844
GSE21653	GSM540190	Luminal_A	5.614709844
GSE21653	GSM540191	Luminal_A	6.475733431
GSE21653	GSM540192	Luminal_A	6.599912842
GSE21653	GSM540193	Luminal_A	6.087462841
GSE21653	GSM540194	Luminal_A	4.64385619
GSE21653	GSM540197	Luminal_A	6.22881869
GSE21653	GSM540198	Luminal_A	7.055282436
GSE21653	GSM540199	Luminal_A	8.22881869
GSE21653	GSM540200	Luminal_A	7.033423002
GSE21653	GSM540202	Luminal_A	5.554588852
GSE21653	GSM540203	Luminal_A	5.247927513
GSE21653	GSM540204	Luminal_A	5.087462841
GSE21653	GSM540205	Luminal_A	5.087462841
GSE21653	GSM540207	Luminal_A	7.499845887
GSE21653	GSM540210	Luminal_A	6.768184325
GSE21653	GSM540211	Luminal_A	5.129283017
GSE21653	GSM540212	Luminal_A	5.64385619
GSE21653	GSM540220	Luminal_A	5.781359714
GSE21653	GSM540222	Luminal_A	6.303780748
GSE21653	GSM540223	Luminal_A	5.357552005
GSE21653	GSM540225	Luminal_A	6.658211483
GSE21653	GSM540227	Luminal_A	5.727920455
GSE21653	GSM540228	Luminal_A	6.64385619
GSE21653	GSM540229	Luminal_A	7.357552005
GSE21653	GSM540230	Luminal_A	7.011227255
GSE21653	GSM540232	Luminal_A	5.754887502
GSE21653	GSM540233	Luminal_A	5.087462841
GSE21653	GSM540234	Luminal_A	6.87036472
GSE21653	GSM540239	Luminal_A	6.977279923
GSE21653	GSM540240	Luminal_A	5.64385619
GSE21653	GSM540241	Luminal_A	5.832890014
GSE21653	GSM540243	Luminal_A	6.62935662
GSE21653	GSM540246	Luminal_A	7.417852515
GSE21653	GSM540247	Luminal_A	6.700439718
GSE21653	GSM540258	Luminal_A	6.108524457

GSE21653	GSM540262	Luminal_A	7.033423002
GSE21653	GSM540275	Luminal_A	6.475733431
GSE21653	GSM540279	Luminal_A	6.044394119
GSE21653	GSM540280	Luminal_A	7.392317423
GSE21653	GSM540286	Luminal_A	6.087462841
GSE21653	GSM540287	Luminal_A	7.636624621
GSE21653	GSM540296	Luminal_A	6.894817763
GSE21653	GSM540305	Luminal_A	6.129283017
GSE21653	GSM540313	Luminal_A	6.64385619
GSE21653	GSM540315	Luminal_A	5.491853096
GSE21653	GSM540317	Luminal_A	4.95419631
GSE21653	GSM540318	Luminal_A	5.459431619
GSE21653	GSM540321	Luminal_A	5.087462841
GSE21653	GSM540322	Luminal_A	5.426264755
GSE21653	GSM540324	Luminal_A	6
GSE21653	GSM540328	Luminal_A	4.523561956
GSE21653	GSM540329	Luminal_A	5.977279923
GSE21653	GSM540330	Luminal_A	4.906890596
GSE21653	GSM540334	Luminal_A	5.321928095
GSE21653	GSM540335	Luminal_A	5.392317423
GSE21653	GSM540337	Luminal_A	4.807354922
GSE21653	GSM540338	Luminal_A	6.209453366
GSE21653	GSM540340	Luminal_A	6.882643049
GSE21653	GSM540344	Luminal_A	4.857980995
GSE21653	GSM540347	Luminal_A	7.257387843
GSE21653	GSM540349	Luminal_A	8.044394119
GSE21653	GSM540350	Luminal_A	6.392317423
GSE21653	GSM540363	Luminal_A	7.651051691
GSE31448	GSM781517	Luminal_A	6.129283017
GSE31448	GSM781525	Luminal_A	6.64385619
GSE31448	GSM781552	Luminal_A	6.882643049
GSE31448	GSM781454	Luminal_A	5.64385619
GSE31448	GSM781402	Luminal_A	5.781359714
GSE31448	GSM781453	Luminal_A	6.977279923
GSE31448	GSM781405	Luminal_A	6.475733431
GSE31448	GSM781389	Luminal_A	5.857980995
GSE31448	GSM781443	Luminal_A	7.357552005
GSE31448	GSM781412	Luminal_A	7.055282436
GSE31448	GSM781541	Luminal_A	5.977279923
GSE31448	GSM781575	Luminal_A	7.651051691
GSE31448	GSM781436	Luminal_A	6.303780748
GSE31448	GSM781556	Luminal_A	4.857980995
GSE31448	GSM781547	Luminal_A	5.392317423
GSE31448	GSM781367	Luminal_A	6.022367813
GSE31448	GSM781404	Luminal_A	5.614709844
GSE31448	GSM781534	Luminal_A	5.426264755

GSE31448	GSM781549	Luminal_A	4.807354922
GSE31448	GSM781529	Luminal_A	4.95419631
GSE31448	GSM781439	Luminal_A	6.658211483
GSE31448	GSM781358	Luminal_A	5.044394119
GSE31448	GSM781446	Luminal_A	5.754887502
GSE31448	GSM781489	Luminal_A	6.475733431
GSE31448	GSM781444	Luminal_A	7.011227255
GSE31448	GSM781542	Luminal_A	4.906890596
GSE31448	GSM781559	Luminal_A	7.257387843
GSE31448	GSM781460	Luminal_A	7.417852515
GSE31448	GSM781411	Luminal_A	6.22881869
GSE31448	GSM781533	Luminal_A	5.087462841
GSE31448	GSM781340	Luminal_A	6.569855608
GSE31448	GSM781399	Luminal_A	7.294620749
GSE31448	GSM781413	Luminal_A	8.22881869
GSE31448	GSM781374	Luminal_A	7.06608919
GSE31448	GSM781437	Luminal_A	5.357552005
GSE31448	GSM781424	Luminal_A	6.768184325
GSE31448	GSM781546	Luminal_A	5.321928095
GSE31448	GSM781448	Luminal_A	6.87036472
GSE31448	GSM781392	Luminal_A	5.781359714
GSE31448	GSM781499	Luminal_A	6.087462841
GSE31448	GSM781550	Luminal_A	6.209453366
GSE31448	GSM781364	Luminal_A	5.832890014
GSE31448	GSM781393	Luminal_A	6.614709844
GSE31448	GSM781508	Luminal_A	6.894817763
GSE31448	GSM781377	Luminal_A	5.357552005
GSE31448	GSM781527	Luminal_A	5.491853096
GSE31448	GSM781493	Luminal_A	6.044394119
GSE31448	GSM781347	Luminal_A	6.022367813
GSE31448	GSM781406	Luminal_A	6.599912842
GSE31448	GSM781350	Luminal_A	4.321928095
GSE31448	GSM781418	Luminal_A	5.087462841
GSE31448	GSM781359	Luminal_A	5.930737338
GSE31448	GSM781403	Luminal_A	5.614709844
GSE31448	GSM781416	Luminal_A	5.554588852
GSE31448	GSM781425	Luminal_A	5.129283017
GSE31448	GSM781417	Luminal_A	5.247927513
GSE31448	GSM781348	Luminal_A	7.266786541
GSE31448	GSM781530	Luminal_A	5.459431619
GSE31448	GSM781326	Luminal_A	7.199672345
GSE31448	GSM781441	Luminal_A	5.727920455
GSE31448	GSM781455	Luminal_A	5.832890014
GSE31448	GSM781328	Luminal_A	5.832890014
GSE31448	GSM781385	Luminal_A	7.348728154
GSE31448	GSM781383	Luminal_A	4.906890596

GSE31448	GSM781414	Luminal_A	7.033423002
GSE31448	GSM781472	Luminal_A	6.108524457
GSE31448	GSM781387	Luminal_A	6.977279923
GSE31448	GSM781407	Luminal_A	6.087462841
GSE31448	GSM781442	Luminal_A	6.64385619
GSE31448	GSM781461	Luminal_A	6.700439718
GSE31448	GSM781336	Luminal_A	7.285402219
GSE31448	GSM781349	Luminal_A	5.614709844
GSE31448	GSM781500	Luminal_A	7.636624621
GSE31448	GSM781343	Luminal_A	7.383704292
GSE31448	GSM781494	Luminal_A	7.392317423
GSE31448	GSM781540	Luminal_A	4.523561956
GSE31448	GSM781434	Luminal_A	5.781359714
GSE31448	GSM781384	Luminal_A	6.266786541
GSE31448	GSM781421	Luminal_A	7.499845887
GSE31448	GSM781476	Luminal_A	7.033423002
GSE31448	GSM781457	Luminal_A	6.62935662
GSE31448	GSM781386	Luminal_A	7.238404739
GSE31448	GSM781426	Luminal_A	5.64385619
GSE31448	GSM781408	Luminal_A	4.64385619
GSE31448	GSM781447	Luminal_A	5.087462841
GSE31448	GSM781561	Luminal_A	8.044394119
GSE31448	GSM781419	Luminal_A	5.087462841
GSE31448	GSM781536	Luminal_A	6
GSE31448	GSM781562	Luminal_A	6.392317423
GSE31448	GSM781316	Luminal_A	5.321928095
GSE45827	GSM1116180	Luminal_A	6.554588852
GSE45827	GSM1116181	Luminal_A	5.977279923
GSE45827	GSM1116184	Luminal_A	4.700439718
GSE45827	GSM1116187	Luminal_A	5.087462841
GSE45827	GSM1116190	Luminal_A	6.044394119
GSE45827	GSM1116191	Luminal_A	5.523561956
GSE45827	GSM1116194	Luminal_A	5.459431619
GSE45827	GSM1116195	Luminal_A	5.754887502
GSE45827	GSM1116199	Luminal_A	5.614709844
GSE45827	GSM1116200	Luminal_A	5.169925001
GSE45827	GSM1116203	Luminal_A	6.303780748
GSE45827	GSM1116204	Luminal_A	5.044394119
GSE45827	GSM1116205	Luminal_A	5.754887502
GSE45827	GSM1116208	Luminal_A	5.672425342
GSE45827	GSM1116209	Luminal_A	4.807354922
GSE45827	GSM1116213	Luminal_A	5.169925001
GSE45827	GSM1116216	Luminal_A	4.754887502
GSE45827	GSM1116217	Luminal_A	5.129283017
GSE45827	GSM1116220	Luminal_A	6.169925001
GSE45827	GSM1116221	Luminal_A	4.392317423

GSE45827	GSM1116222	Luminal_A	5.882643049
GSE45827	GSM1116223	Luminal_A	5.754887502
GSE45827	GSM1116224	Luminal_A	6.044394119
GSE45827	GSM1116225	Luminal_A	5.857980995
GSE45827	GSM1116228	Luminal_A	5.554588852
GSE45827	GSM1116231	Luminal_A	5.554588852
GSE45827	GSM1116232	Luminal_A	5.285402219
GSE45827	GSM1116234	Luminal_A	5.392317423
GSE45827	GSM1116235	Luminal_A	5.392317423
GSE48391	GSM1176873	Luminal_A	5.700439718
GSE48391	GSM1176874	Luminal_A	6.108524457
GSE48391	GSM1176875	Luminal_A	4.169925001
GSE48391	GSM1176884	Luminal_A	6.569855608
GSE48391	GSM1176891	Luminal_A	6.409390936
GSE48391	GSM1176893	Luminal_A	5.930737338
GSE48391	GSM1176894	Luminal_A	5.554588852
GSE48391	GSM1176908	Luminal_A	5.087462841
GSE48391	GSM1176914	Luminal_A	5.64385619
GSE48391	GSM1176923	Luminal_A	5.491853096
GSE48391	GSM1176924	Luminal_A	4.087462841
GSE48391	GSM1176925	Luminal_A	5.700439718
GSE48391	GSM1176926	Luminal_A	5.781359714
GSE48391	GSM1176928	Luminal_A	6.50779464
GSE48391	GSM1176936	Luminal_A	5.95419631
GSE48391	GSM1176937	Luminal_A	5.930737338
GSE48391	GSM1176938	Luminal_A	6.266786541
GSE48391	GSM1176941	Luminal_A	4
GSE48391	GSM1176942	Luminal_A	6.22881869
GSE48391	GSM1176949	Luminal_A	6.321928095
GSE48391	GSM1176950	Luminal_A	6.087462841
GSE48391	GSM1176952	Luminal_A	6.658211483
GSE65216	GSM1589064	Luminal_A	5.754887502
GSE65216	GSM1589065	Luminal_A	5.882643049
GSE65216	GSM1589066	Luminal_A	5.285402219
GSE65216	GSM1589067	Luminal_A	5.754887502
GSE65216	GSM1589068	Luminal_A	6.554588852
GSE65216	GSM1589069	Luminal_A	4.392317423
GSE65216	GSM1589070	Luminal_A	5.169925001
GSE65216	GSM1589071	Luminal_A	5.554588852
GSE65216	GSM1589072	Luminal_A	6.044394119
GSE65216	GSM1589073	Luminal_A	5.977279923
GSE65216	GSM1589074	Luminal_A	5.044394119
GSE65216	GSM1589075	Luminal_A	5.392317423
GSE65216	GSM1589076	Luminal_A	5.392317423
GSE65216	GSM1589077	Luminal_A	5.523561956
GSE65216	GSM1589078	Luminal_A	5.554588852

GSE65216	GSM1589079	Luminal_A	4.807354922
GSE65216	GSM1589080	Luminal_A	5.459431619
GSE65216	GSM1589081	Luminal_A	5.614709844
GSE65216	GSM1589082	Luminal_A	5.169925001
GSE65216	GSM1589083	Luminal_A	5.087462841
GSE65216	GSM1589084	Luminal_A	5.754887502
GSE65216	GSM1589085	Luminal_A	5.129283017
GSE65216	GSM1589086	Luminal_A	6.044394119
GSE65216	GSM1589087	Luminal_A	6.169925001
GSE65216	GSM1589088	Luminal_A	5.857980995
GSE65216	GSM1589089	Luminal_A	4.700439718
GSE65216	GSM1589090	Luminal_A	4.754887502
GSE65216	GSM1589091	Luminal_A	6.303780748
GSE65216	GSM1589092	Luminal_A	5.672425342
GSE20713	GSM519725	Luminal_B	6.14974712
GSE20713	GSM519730	Luminal_B	7.375039431
GSE20713	GSM519731	Luminal_B	7.483815777
GSE20713	GSM519732	Luminal_B	6.491853096
GSE20713	GSM519733	Luminal_B	6.658211483
GSE20713	GSM519737	Luminal_B	6.475733431
GSE20713	GSM519742	Luminal_B	7.011227255
GSE20713	GSM519743	Luminal_B	5.584962501
GSE20713	GSM519748	Luminal_B	6.129283017
GSE20713	GSM519750	Luminal_B	6.977279923
GSE20713	GSM519752	Luminal_B	7.033423002
GSE20713	GSM519754	Luminal_B	7.022367813
GSE20713	GSM519771	Luminal_B	8.06608919
GSE20713	GSM519772	Luminal_B	6.044394119
GSE20713	GSM519773	Luminal_B	6.918863237
GSE20713	GSM519775	Luminal_B	8.011227255
GSE20713	GSM519776	Luminal_B	8.6794801
GSE20713	GSM519783	Luminal_B	8.005624549
GSE20713	GSM519784	Luminal_B	8.071462363
GSE20713	GSM519794	Luminal_B	8.038918989
GSE20713	GSM519801	Luminal_B	6.475733431
GSE20713	GSM519803	Luminal_B	6.426264755
GSE21653	GSM540316	Luminal_B	8.189824559
GSE21653	GSM540311	Luminal_B	7.851749041
GSE21653	GSM540303	Luminal_B	8.781359714
GSE21653	GSM540320	Luminal_B	5.523561956
GSE21653	GSM540306	Luminal_B	7.727920455
GSE21653	GSM540142	Luminal_B	7.658211483
GSE21653	GSM540166	Luminal_B	7.139551352
GSE21653	GSM540342	Luminal_B	8.640244936
GSE21653	GSM540245	Luminal_B	7.321928095
GSE21653	GSM540336	Luminal_B	8.144658243

GSE21653	GSM540148	Luminal_B	7.266786541
GSE21653	GSM540226	Luminal_B	7.607330314
GSE21653	GSM540182	Luminal_B	8.038918989
GSE21653	GSM540277	Luminal_B	7.483815777
GSE21653	GSM540266	Luminal_B	7.483815777
GSE21653	GSM540284	Luminal_B	7.392317423
GSE21653	GSM540152	Luminal_B	7.787902559
GSE21653	GSM540231	Luminal_B	6.357552005
GSE21653	GSM540270	Luminal_B	7.189824559
GSE21653	GSM540177	Luminal_B	8.13442632
GSE21653	GSM540269	Luminal_B	8.271463028
GSE21653	GSM540341	Luminal_B	6.894817763
GSE21653	GSM540116	Luminal_B	6.965784285
GSE21653	GSM540221	Luminal_B	8.77148947
GSE21653	GSM540332	Luminal_B	8.247927513
GSE21653	GSM540295	Luminal_B	7.820178962
GSE21653	GSM540165	Luminal_B	7.894817763
GSE21653	GSM540168	Luminal_B	6.87036472
GSE21653	GSM540196	Luminal_B	7.348728154
GSE21653	GSM540253	Luminal_B	6.523561956
GSE21653	GSM540161	Luminal_B	6.894817763
GSE21653	GSM540201	Luminal_B	9.164906927
GSE21653	GSM540108	Luminal_B	6.491853096
GSE21653	GSM540278	Luminal_B	9.366322214
GSE21653	GSM540110	Luminal_B	7.426264755
GSE21653	GSM540299	Luminal_B	7.159871337
GSE21653	GSM540131	Luminal_B	7.499845887
GSE21653	GSM540109	Luminal_B	7.189824559
GSE21653	GSM540128	Luminal_B	8.95419631
GSE21653	GSM540120	Luminal_B	7.787902559
GSE21653	GSM540139	Luminal_B	5
GSE21653	GSM540149	Luminal_B	7.06608919
GSE21653	GSM540137	Luminal_B	7.011227255
GSE21653	GSM540343	Luminal_B	9.87036472
GSE21653	GSM540326	Luminal_B	5.64385619
GSE21653	GSM540333	Luminal_B	6.754887502
GSE21653	GSM540224	Luminal_B	7.577428828
GSE21653	GSM540123	Luminal_B	7.475733431
GSE21653	GSM540298	Luminal_B	6.658211483
GSE21653	GSM540108	Luminal_B	6.459431619
GSE21653	GSM540109	Luminal_B	7.14974712
GSE21653	GSM540110	Luminal_B	7.392317423
GSE21653	GSM540116	Luminal_B	6.942514505
GSE21653	GSM540120	Luminal_B	7.77478706
GSE21653	GSM540123	Luminal_B	7.451211112
GSE21653	GSM540128	Luminal_B	8.936637939

GSE21653	GSM540131	Luminal_B	7.475733431
GSE21653	GSM540137	Luminal_B	6.977279923
GSE21653	GSM540139	Luminal_B	4.95419631
GSE21653	GSM540142	Luminal_B	7.636624621
GSE21653	GSM540148	Luminal_B	7.238404739
GSE21653	GSM540149	Luminal_B	7.044394119
GSE21653	GSM540152	Luminal_B	7.761551232
GSE21653	GSM540161	Luminal_B	6.882643049
GSE21653	GSM540165	Luminal_B	7.876516947
GSE21653	GSM540166	Luminal_B	7.118941073
GSE21653	GSM540168	Luminal_B	6.845490051
GSE21653	GSM540177	Luminal_B	8.124121312
GSE21653	GSM540182	Luminal_B	8.022367813
GSE21653	GSM540196	Luminal_B	7.330916878
GSE21653	GSM540201	Luminal_B	9.147204925
GSE21653	GSM540221	Luminal_B	8.74819285
GSE21653	GSM540224	Luminal_B	7.562242424
GSE21653	GSM540226	Luminal_B	7.584962501
GSE21653	GSM540231	Luminal_B	6.303780748
GSE21653	GSM540245	Luminal_B	7.303780748
GSE21653	GSM540253	Luminal_B	6.491853096
GSE21653	GSM540266	Luminal_B	7.46760555
GSE21653	GSM540269	Luminal_B	8.257387843
GSE21653	GSM540270	Luminal_B	7.169925001
GSE21653	GSM540277	Luminal_B	7.46760555
GSE21653	GSM540278	Luminal_B	9.346513733
GSE21653	GSM540284	Luminal_B	7.366322214
GSE21653	GSM540295	Luminal_B	7.8008999
GSE21653	GSM540298	Luminal_B	6.64385619
GSE21653	GSM540299	Luminal_B	7.139551352
GSE21653	GSM540303	Luminal_B	8.761551232
GSE21653	GSM540306	Luminal_B	7.707359132
GSE21653	GSM540311	Luminal_B	7.826548487
GSE21653	GSM540316	Luminal_B	8.169925001
GSE21653	GSM540320	Luminal_B	5.491853096
GSE21653	GSM540326	Luminal_B	5.614709844
GSE21653	GSM540332	Luminal_B	8.224001674
GSE21653	GSM540333	Luminal_B	6.741466986
GSE21653	GSM540336	Luminal_B	8.124121312
GSE21653	GSM540341	Luminal_B	6.882643049
GSE21653	GSM540342	Luminal_B	8.622051819
GSE21653	GSM540343	Luminal_B	9.861086906
GSE31448	GSM781544	Luminal_B	8.224001674
GSE31448	GSM781548	Luminal_B	8.124121312
GSE31448	GSM781351	Luminal_B	6.977279923
GSE31448	GSM781484	Luminal_B	7.169925001

GSE31448	GSM781553	Luminal_B	6.882643049
GSE31448	GSM781554	Luminal_B	8.622051819
GSE31448	GSM781353	Luminal_B	4.95419631
GSE31448	GSM781483	Luminal_B	8.257387843
GSE31448	GSM781555	Luminal_B	9.861086906
GSE31448	GSM781356	Luminal_B	7.636624621
GSE31448	GSM781445	Luminal_B	6.303780748
GSE31448	GSM781391	Luminal_B	8.124121312
GSE31448	GSM781363	Luminal_B	7.044394119
GSE31448	GSM781415	Luminal_B	9.147204925
GSE31448	GSM781545	Luminal_B	6.741466986
GSE31448	GSM781523	Luminal_B	7.826548487
GSE31448	GSM781538	Luminal_B	5.614709844
GSE31448	GSM781362	Luminal_B	7.238404739
GSE31448	GSM781345	Luminal_B	7.475733431
GSE31448	GSM781379	Luminal_B	7.876516947
GSE31448	GSM781342	Luminal_B	8.936637939
GSE31448	GSM781410	Luminal_B	7.330916878
GSE31448	GSM781435	Luminal_B	8.74819285
GSE31448	GSM781330	Luminal_B	6.942514505
GSE31448	GSM781532	Luminal_B	5.491853096
GSE31448	GSM781440	Luminal_B	7.584962501
GSE31448	GSM781491	Luminal_B	7.46760555
GSE31448	GSM781322	Luminal_B	6.459431619
GSE31448	GSM781515	Luminal_B	8.761551232
GSE31448	GSM781467	Luminal_B	6.491853096
GSE31448	GSM781380	Luminal_B	7.118941073
GSE31448	GSM781507	Luminal_B	7.8008999
GSE31448	GSM781518	Luminal_B	7.707359132
GSE31448	GSM781511	Luminal_B	7.139551352
GSE31448	GSM781375	Luminal_B	6.882643049
GSE31448	GSM781497	Luminal_B	7.366322214
GSE31448	GSM781438	Luminal_B	7.562242424
GSE31448	GSM781480	Luminal_B	7.46760555
GSE31448	GSM781334	Luminal_B	7.77478706
GSE31448	GSM781396	Luminal_B	8.022367813
GSE31448	GSM781492	Luminal_B	9.346513733
GSE31448	GSM781382	Luminal_B	6.845490051
GSE31448	GSM781510	Luminal_B	6.64385619
GSE31448	GSM781366	Luminal_B	7.761551232
GSE31448	GSM781337	Luminal_B	7.451211112
GSE31448	GSM781459	Luminal_B	7.303780748
GSE31448	GSM781528	Luminal_B	8.169925001
GSE31448	GSM781324	Luminal_B	7.392317423
GSE31448	GSM781323	Luminal_B	7.14974712
GSE45827	GSM1116182	Luminal_B	6.845490051

GSE45827	GSM1116183	Luminal_B	6.832890014
GSE45827	GSM1116185	Luminal_B	7.209453366
GSE45827	GSM1116186	Luminal_B	6.658211483
GSE45827	GSM1116188	Luminal_B	6.108524457
GSE45827	GSM1116189	Luminal_B	6.741466986
GSE45827	GSM1116192	Luminal_B	5.169925001
GSE45827	GSM1116193	Luminal_B	6.442943496
GSE45827	GSM1116196	Luminal_B	6.22881869
GSE45827	GSM1116197	Luminal_B	7.17990909
GSE45827	GSM1116198	Luminal_B	7.22881869
GSE45827	GSM1116201	Luminal_B	7.033423002
GSE45827	GSM1116202	Luminal_B	6.087462841
GSE45827	GSM1116206	Luminal_B	5.554588852
GSE45827	GSM1116207	Luminal_B	8.430452552
GSE45827	GSM1116210	Luminal_B	5.906890596
GSE45827	GSM1116211	Luminal_B	6.491853096
GSE45827	GSM1116212	Luminal_B	6.475733431
GSE45827	GSM1116214	Luminal_B	7.700439718
GSE45827	GSM1116215	Luminal_B	6.022367813
GSE45827	GSM1116218	Luminal_B	5.491853096
GSE45827	GSM1116219	Luminal_B	6.658211483
GSE45827	GSM1116226	Luminal_B	5.64385619
GSE45827	GSM1116227	Luminal_B	6.894817763
GSE45827	GSM1116229	Luminal_B	5.700439718
GSE45827	GSM1116230	Luminal_B	7.46760555
GSE45827	GSM1116233	Luminal_B	8.379378367
GSE45827	GSM1116236	Luminal_B	6.475733431
GSE45827	GSM1116237	Luminal_B	7.348728154
GSE45827	GSM1116238	Luminal_B	6.686500527
GSE48391	GSM1176879	Luminal_B	6.857980995
GSE48391	GSM1176883	Luminal_B	8.30833903
GSE48391	GSM1176902	Luminal_B	7.451211112
GSE48391	GSM1176904	Luminal_B	7.569855608
GSE48391	GSM1176905	Luminal_B	6.658211483
GSE48391	GSM1176917	Luminal_B	6.768184325
GSE48391	GSM1176922	Luminal_B	6.554588852
GSE48391	GSM1176927	Luminal_B	6.794415866
GSE48391	GSM1176929	Luminal_B	7.46760555
GSE48391	GSM1176931	Luminal_B	5.554588852
GSE48391	GSM1176939	Luminal_B	8.499845887
GSE48391	GSM1176940	Luminal_B	6.87036472
GSE48391	GSM1176943	Luminal_B	6.965784285
GSE48391	GSM1176945	Luminal_B	7.50779464
GSE48391	GSM1176947	Luminal_B	7.238404739
GSE65216	GSM1589093	Luminal_B	8.379378367
GSE65216	GSM1589094	Luminal_B	7.209453366

GSE65216	GSM1589095	Luminal_B	5.64385619
GSE65216	GSM1589097	Luminal_B	6.741466986
GSE65216	GSM1589098	Luminal_B	7.033423002
GSE65216	GSM1589099	Luminal_B	6.22881869
GSE65216	GSM1589101	Luminal_B	7.46760555
GSE65216	GSM1589103	Luminal_B	5.906890596
GSE65216	GSM1589105	Luminal_B	6.832890014
GSE65216	GSM1589107	Luminal_B	7.17990909
GSE65216	GSM1589109	Luminal_B	6.087462841
GSE65216	GSM1589110	Luminal_B	7.348728154
GSE65216	GSM1589111	Luminal_B	6.845490051
GSE65216	GSM1589112	Luminal_B	6.475733431
GSE65216	GSM1589113	Luminal_B	6.491853096
GSE65216	GSM1589114	Luminal_B	7.700439718
GSE65216	GSM1589115	Luminal_B	5.169925001
GSE65216	GSM1589116	Luminal_B	6.022367813
GSE65216	GSM1589117	Luminal_B	6.475733431
GSE65216	GSM1589118	Luminal_B	8.430452552
GSE65216	GSM1589119	Luminal_B	5.700439718
GSE65216	GSM1589120	Luminal_B	6.108524457
GSE65216	GSM1589121	Luminal_B	7.22881869
GSE65216	GSM1589122	Luminal_B	6.658211483
GSE65216	GSM1589123	Luminal_B	6.658211483
GSE65216	GSM1589124	Luminal_B	6.894817763
GSE65216	GSM1589125	Luminal_B	5.554588852
GSE65216	GSM1589126	Luminal_B	6.442943496
GSE65216	GSM1589127	Luminal_B	6.686500527
GSE65216	GSM1589128	Luminal_B	5.491853096
GSE12763	GSM320217	HER2	7.714245518
GSE12763	GSM320218	HER2	7.247927513
GSE12763	GSM320219	HER2	7.741466986
GSE12763	GSM320222	HER2	7.942514505
GSE12763	GSM320231	HER2	9.5980525
GSE12763	GSM320241	HER2	7.876516947
GSE12763	GSM320242	HER2	8.577428828
GSE13787	GSM346894	HER2	6.794415866
GSE13787	GSM346895	HER2	7.209453366
GSE13787	GSM346896	HER2	7.54689446
GSE13787	GSM346897	HER2	7.672425342
GSE13787	GSM346898	HER2	7.303780748
GSE13787	GSM346899	HER2	6.807354922
GSE13787	GSM346900	HER2	8.129283017
GSE13787	GSM346901	HER2	8.326429487
GSE13787	GSM346902	HER2	7.77478706
GSE13787	GSM346903	HER2	8.569855608
GSE13787	GSM346904	HER2	8.519636253

GSE17907	GSM447197	HER2	7.417852515
GSE17907	GSM447198	HER2	10.03479896
GSE17907	GSM447199	HER2	9.167418146
GSE17907	GSM447200	HER2	7.339850003
GSE17907	GSM447201	HER2	5.584962501
GSE17907	GSM447202	HER2	7.159871337
GSE17907	GSM447203	HER2	7.330916878
GSE17907	GSM447209	HER2	7.348728154
GSE17907	GSM447210	HER2	8.46760555
GSE17907	GSM447211	HER2	8.672425342
GSE17907	GSM447212	HER2	7.845490051
GSE17907	GSM447213	HER2	8.312882955
GSE17907	GSM447214	HER2	7.832890014
GSE17907	GSM447215	HER2	9.649256178
GSE17907	GSM447217	HER2	6.569855608
GSE17907	GSM447219	HER2	7.491853096
GSE17907	GSM447220	HER2	8.011227255
GSE17907	GSM447221	HER2	8.781359714
GSE17907	GSM447222	HER2	6.894817763
GSE17907	GSM447226	HER2	7.330916878
GSE17907	GSM447228	HER2	6.285402219
GSE17907	GSM447229	HER2	7.912889336
GSE17907	GSM447230	HER2	8.62935662
GSE17907	GSM447231	HER2	5.906890596
GSE17907	GSM447232	HER2	6.832890014
GSE17907	GSM447233	HER2	7.781359714
GSE17907	GSM447235	HER2	7.46760555
GSE17907	GSM447236	HER2	9.566054038
GSE17907	GSM447237	HER2	7.076815597
GSE17907	GSM447238	HER2	7.636624621
GSE17907	GSM447240	HER2	8.603626345
GSE17907	GSM447241	HER2	6.459431619
GSE17907	GSM447242	HER2	7.754887502
GSE17907	GSM447246	HER2	9.167418146
GSE17907	GSM447247	HER2	6.754887502
GSE20713	GSM519722	HER2	7.22881869
GSE20713	GSM519723	HER2	7.330916878
GSE20713	GSM519724	HER2	6.768184325
GSE20713	GSM519728	HER2	6.832890014
GSE20713	GSM519740	HER2	6.189824559
GSE20713	GSM519749	HER2	7.636624621
GSE20713	GSM519751	HER2	8.965784285
GSE20713	GSM519756	HER2	8.28077077
GSE20713	GSM519767	HER2	6.62935662
GSE20713	GSM519769	HER2	7.965784285
GSE20713	GSM519777	HER2	7.499845887

GSE20713	GSM519778	HER2	7.108524457
GSE20713	GSM519781	HER2	7.936637939
GSE20713	GSM519782	HER2	6.977279923
GSE20713	GSM519785	HER2	6.599912842
GSE20713	GSM519786	HER2	7.247927513
GSE20713	GSM519797	HER2	7.707359132
GSE20713	GSM519799	HER2	6.672425342
GSE20713	GSM519800	HER2	8.174925683
GSE20713	GSM519805	HER2	6.189824559
GSE20713	GSM519806	HER2	7.515699838
GSE21653	GSM540323	HER2	6.357552005
GSE21653	GSM540309	HER2	6.906890596
GSE21653	GSM540250	HER2	7.209453366
GSE21653	GSM540180	HER2	6.569855608
GSE21653	GSM540251	HER2	8.312882955
GSE21653	GSM540257	HER2	7.781359714
GSE21653	GSM540370	HER2	7.523561956
GSE21653	GSM540213	HER2	8.784634846
GSE21653	GSM540265	HER2	8.353146825
GSE21653	GSM540111	HER2	7.348728154
GSE21653	GSM540283	HER2	7.672425342
GSE21653	GSM540354	HER2	7.74819285
GSE21653	GSM540216	HER2	7.845490051
GSE21653	GSM540272	HER2	7.209453366
GSE21653	GSM540268	HER2	9.649256178
GSE21653	GSM540358	HER2	9.554588852
GSE21653	GSM540297	HER2	8.22881869
GSE21653	GSM540289	HER2	8.011227255
GSE21653	GSM540339	HER2	6.285402219
GSE21653	GSM540162	HER2	7.592457037
GSE21653	GSM540346	HER2	6.129283017
GSE21653	GSM540327	HER2	7.700439718
GSE21653	GSM540174	HER2	8.672425342
GSE21653	GSM540274	HER2	7.330916878
GSE21653	GSM540111	HER2	7.321928095
GSE21653	GSM540162	HER2	7.569855608
GSE21653	GSM540174	HER2	8.654636029
GSE21653	GSM540180	HER2	6.539158811
GSE21653	GSM540213	HER2	8.768184325
GSE21653	GSM540216	HER2	7.826548487
GSE21653	GSM540250	HER2	7.17990909
GSE21653	GSM540251	HER2	8.299208018
GSE21653	GSM540257	HER2	7.761551232
GSE21653	GSM540265	HER2	8.330916878
GSE21653	GSM540268	HER2	9.627533884
GSE21653	GSM540272	HER2	7.17990909

GSE21653	GSM540274	HER2	7.312882955
GSE21653	GSM540283	HER2	7.658211483
GSE21653	GSM540289	HER2	7.994353437
GSE21653	GSM540297	HER2	8.204571144
GSE21653	GSM540309	HER2	6.882643049
GSE21653	GSM540323	HER2	6.321928095
GSE21653	GSM540327	HER2	7.6794801
GSE21653	GSM540339	HER2	6.247927513
GSE21653	GSM540346	HER2	6.108524457
GSE21653	GSM540354	HER2	7.721099189
GSE21653	GSM540358	HER2	9.539158811
GSE21653	GSM540370	HER2	7.499845887
GSE31448	GSM781501	HER2	7.994353437
GSE31448	GSM781464	HER2	7.17990909
GSE31448	GSM781551	HER2	6.247927513
GSE31448	GSM781465	HER2	8.299208018
GSE31448	GSM781496	HER2	7.658211483
GSE31448	GSM781394	HER2	6.539158811
GSE31448	GSM781521	HER2	6.882643049
GSE31448	GSM781325	HER2	7.321928095
GSE31448	GSM781539	HER2	7.6794801
GSE31448	GSM781509	HER2	8.204571144
GSE31448	GSM781427	HER2	8.768184325
GSE31448	GSM781471	HER2	7.761551232
GSE31448	GSM781558	HER2	6.108524457
GSE31448	GSM781388	HER2	8.654636029
GSE31448	GSM781566	HER2	7.721099189
GSE31448	GSM781479	HER2	8.330916878
GSE31448	GSM781482	HER2	9.627533884
GSE31448	GSM781570	HER2	9.539158811
GSE31448	GSM781582	HER2	7.499845887
GSE31448	GSM781488	HER2	7.312882955
GSE31448	GSM781376	HER2	7.569855608
GSE31448	GSM781486	HER2	7.17990909
GSE31448	GSM781430	HER2	7.826548487
GSE31448	GSM781306	HER2	6.409390936
GSE31448	GSM781535	HER2	6.321928095
GSE45827	GSM1116086	HER2	6.794415866
GSE45827	GSM1116088	HER2	7.199672345
GSE45827	GSM1116089	HER2	7.434628228
GSE45827	GSM1116095	HER2	7.672425342
GSE45827	GSM1116096	HER2	7.62935662
GSE45827	GSM1116097	HER2	7.303780748
GSE45827	GSM1116098	HER2	6.794415866
GSE45827	GSM1116100	HER2	6.64385619
GSE45827	GSM1116103	HER2	8.257387843

GSE45827	GSM1116104	HER2	7.434628228
GSE45827	GSM1116105	HER2	7.768184325
GSE45827	GSM1116107	HER2	7.820178962
GSE45827	GSM1116116	HER2	9.144658243
GSE45827	GSM1116117	HER2	8.366322214
GSE45827	GSM1116121	HER2	7.348728154
GSE45827	GSM1116122	HER2	7.417852515
GSE45827	GSM1116123	HER2	6.930737338
GSE45827	GSM1116126	HER2	7.87036472
GSE45827	GSM1116127	HER2	6.62935662
GSE45827	GSM1116129	HER2	7.592457037
GSE45827	GSM1116132	HER2	7.375039431
GSE45827	GSM1116133	HER2	9.674192268
GSE45827	GSM1116134	HER2	6.768184325
GSE45827	GSM1116137	HER2	6.169925001
GSE45827	GSM1116140	HER2	7.21916852
GSE45827	GSM1116141	HER2	9.758223215
GSE45827	GSM1116151	HER2	6.807354922
GSE45827	GSM1116152	HER2	7.924812504
GSE45827	GSM1116153	HER2	8
GSE45827	GSM1116154	HER2	6.14974712
GSE48391	GSM1176877	HER2	6.06608919
GSE48391	GSM1176885	HER2	7.475733431
GSE48391	GSM1176888	HER2	5.523561956
GSE48391	GSM1176889	HER2	7.098032083
GSE48391	GSM1176890	HER2	7.303780748
GSE48391	GSM1176896	HER2	7.129283017
GSE48391	GSM1176897	HER2	7.022367813
GSE48391	GSM1176899	HER2	8.442943496
GSE48391	GSM1176901	HER2	7.022367813
GSE48391	GSM1176906	HER2	7.400879436
GSE48391	GSM1176907	HER2	8.113742166
GSE48391	GSM1176915	HER2	7.906890596
GSE48391	GSM1176930	HER2	7.055282436
GSE48391	GSM1176934	HER2	7.044394119
GSE65216	GSM1588972	HER2	7.312882955
GSE65216	GSM1588973	HER2	7.820178962
GSE65216	GSM1588981	HER2	6.794415866
GSE65216	GSM1588989	HER2	7.199672345
GSE65216	GSM1588990	HER2	7.209453366
GSE65216	GSM1588991	HER2	8.243173983
GSE65216	GSM1588992	HER2	8.366322214
GSE65216	GSM1588997	HER2	7.348728154
GSE65216	GSM1588998	HER2	7.06608919
GSE65216	GSM1588999	HER2	7.417852515
GSE65216	GSM1589000	HER2	7.434628228

GSE65216	GSM1589001	HER2	7.539158811
GSE65216	GSM1589002	HER2	6.930737338
GSE65216	GSM1589006	HER2	7.409390936
GSE65216	GSM1589007	HER2	7.87036472
GSE65216	GSM1589008	HER2	6.62935662
GSE65216	GSM1589013	HER2	7.592457037
GSE65216	GSM1589018	HER2	7.826548487
GSE65216	GSM1589019	HER2	7.672425342
GSE65216	GSM1589020	HER2	7.375039431
GSE65216	GSM1589021	HER2	9.674192268
GSE65216	GSM1589022	HER2	6.857980995
GSE65216	GSM1589023	HER2	6.768184325
GSE65216	GSM1589025	HER2	6.169925001
GSE65216	GSM1589026	HER2	7.62935662
GSE65216	GSM1589027	HER2	7.303780748
GSE65216	GSM1589030	HER2	6.794415866
GSE65216	GSM1589033	HER2	7.21916852
GSE65216	GSM1589034	HER2	9.758223215
GSE65216	GSM1589044	HER2	6.64385619
GSE65216	GSM1589046	HER2	8.257387843
GSE65216	GSM1589047	HER2	7.515699838
GSE65216	GSM1589048	HER2	7.434628228
GSE65216	GSM1589049	HER2	7.768184325
GSE65216	GSM1589050	HER2	6.807354922
GSE65216	GSM1589051	HER2	7.924812504
GSE65216	GSM1589052	HER2	8
GSE65216	GSM1589053	HER2	6.14974712
GSE65216	GSM1589057	HER2	9.144658243
GSE65216	GSM1588971	TNBC	8.519636253
GSE65216	GSM1588974	TNBC	8.668884984
GSE65216	GSM1588975	TNBC	9.079484784
GSE65216	GSM1588976	TNBC	9.278449458
GSE65216	GSM1588977	TNBC	8.312882955
GSE65216	GSM1588978	TNBC	8.174925683
GSE65216	GSM1588979	TNBC	9.14974712
GSE65216	GSM1588980	TNBC	9.631177056
GSE65216	GSM1588982	TNBC	8.531381461
GSE65216	GSM1588983	TNBC	8.826548487
GSE65216	GSM1588984	TNBC	10.13570929
GSE65216	GSM1588985	TNBC	8.17990909
GSE65216	GSM1588986	TNBC	8.73809226
GSE65216	GSM1588987	TNBC	9.076815597
GSE65216	GSM1588988	TNBC	8.113742166
GSE65216	GSM1588993	TNBC	7.266786541
GSE65216	GSM1588994	TNBC	7.539158811
GSE65216	GSM1588995	TNBC	9.584962501

GSE65216	GSM1588996	TNBC	9.802516365
GSE65216	GSM1589003	TNBC	7.54689446
GSE65216	GSM1589004	TNBC	7.876516947
GSE65216	GSM1589005	TNBC	8.049848549
GSE65216	GSM1589009	TNBC	8.689997971
GSE65216	GSM1589010	TNBC	9.005624549
GSE65216	GSM1589011	TNBC	9
GSE65216	GSM1589012	TNBC	8.233619677
GSE65216	GSM1589014	TNBC	8.689997971
GSE65216	GSM1589015	TNBC	8.8008999
GSE65216	GSM1589016	TNBC	8.422064766
GSE65216	GSM1589017	TNBC	9.301496195
GSE65216	GSM1589024	TNBC	7.357552005
GSE65216	GSM1589028	TNBC	7.50779464
GSE65216	GSM1589029	TNBC	7.554588852
GSE65216	GSM1589031	TNBC	8.159871337
GSE65216	GSM1589032	TNBC	8.129283017
GSE65216	GSM1589035	TNBC	9.136991112
GSE65216	GSM1589036	TNBC	9.430452552
GSE65216	GSM1589037	TNBC	8.038918989
GSE65216	GSM1589038	TNBC	8.124121312
GSE65216	GSM1589039	TNBC	7.741466986
GSE65216	GSM1589040	TNBC	7.761551232
GSE65216	GSM1589041	TNBC	7.285402219
GSE65216	GSM1589042	TNBC	8.092757141
GSE65216	GSM1589043	TNBC	8.554588852
GSE65216	GSM1589045	TNBC	8.566054038
GSE65216	GSM1589054	TNBC	7.832890014
GSE65216	GSM1589055	TNBC	8.77148947
GSE65216	GSM1589056	TNBC	9.121533517
GSE65216	GSM1589058	TNBC	8.942514505
GSE65216	GSM1589059	TNBC	8.965784285
GSE65216	GSM1589060	TNBC	9.554588852
GSE65216	GSM1589061	TNBC	8.912889336
GSE65216	GSM1589062	TNBC	8.326429487
GSE65216	GSM1589063	TNBC	9.942514505
GSE76275	GSM1974651	TNBC	7.108524457
GSE76275	GSM1974680	TNBC	8.005624549
GSE76275	GSM1974739	TNBC	6.392317423
GSE76275	GSM1974740	TNBC	9.741466986
GSE76275	GSM1974741	TNBC	6.539158811
GSE76275	GSM1974647	TNBC	10.06877828
GSE76275	GSM1974652	TNBC	6.754887502
GSE76275	GSM1974721	TNBC	9.008428622
GSE76275	GSM1974687	TNBC	8.661778098
GSE76275	GSM1974656	TNBC	8.84862294

GSE76275	GSM1974581	TNBC	5.209453366
GSE76275	GSM1974663	TNBC	6.741466986
GSE76275	GSM1974620	TNBC	9.326429487
GSE76275	GSM1974752	TNBC	6.475733431
GSE76275	GSM1974748	TNBC	5.321928095
GSE76275	GSM1974701	TNBC	8.897845456
GSE76275	GSM1974626	TNBC	9.025139562
GSE76275	GSM1974720	TNBC	7.960001932
GSE76275	GSM1974709	TNBC	8.73809226
GSE76275	GSM1974706	TNBC	6.569855608
GSE76275	GSM1974587	TNBC	5.426264755
GSE76275	GSM1974729	TNBC	8.417852515
GSE76275	GSM1974688	TNBC	5.977279923
GSE76275	GSM1974657	TNBC	9.002815016
GSE76275	GSM1974722	TNBC	7.577428828
GSE76275	GSM1974579	TNBC	5.584962501
GSE76275	GSM1974754	TNBC	9.603626345
GSE76275	GSM1974753	TNBC	7.499845887
GSE76275	GSM1974662	TNBC	8.744833837
GSE76275	GSM1974735	TNBC	6.539158811
GSE76275	GSM1974744	TNBC	8.396604781
GSE76275	GSM1974627	TNBC	5.64385619
GSE76275	GSM1974727	TNBC	10.3376219
GSE76275	GSM1974612	TNBC	8.103287808
GSE76275	GSM1974583	TNBC	7.46760555
GSE76275	GSM1974685	TNBC	7.826548487
GSE76275	GSM1974707	TNBC	9.754887502
GSE76275	GSM1974719	TNBC	6.22881869
GSE76275	GSM1974703	TNBC	6.539158811
GSE76275	GSM1974717	TNBC	8.159871337
GSE76275	GSM1974654	TNBC	8.348728154
GSE76275	GSM1974635	TNBC	5.930737338
GSE76275	GSM1974566	TNBC	8.539158811
GSE76275	GSM1974614	TNBC	8.924812504
GSE76275	GSM1974625	TNBC	8.392317423
GSE76275	GSM1974602	TNBC	8.483815777
GSE76275	GSM1974578	TNBC	8.092757141
GSE76275	GSM1974689	TNBC	7.366322214
GSE76275	GSM1974615	TNBC	9.169925001
GSE76275	GSM1974712	TNBC	8.906890596
GSE76275	GSM1974597	TNBC	6.741466986
GSE76275	GSM1974681	TNBC	6.108524457
GSE76275	GSM1974631	TNBC	9.4325419
GSE76275	GSM1974621	TNBC	8.900866808
GSE76275	GSM1974664	TNBC	9.415741768
GSE76275	GSM1974623	TNBC	7.936637939

GSE76275	GSM1974745	TNBC	7.266786541
GSE76275	GSM1974658	TNBC	7.087462841
GSE76275	GSM1974567	TNBC	9.850186838
GSE76275	GSM1974696	TNBC	8.199672345
GSE76275	GSM1974589	TNBC	8.238404739
GSE76275	GSM1974569	TNBC	7.451211112
GSE76275	GSM1974648	TNBC	7.826548487
GSE76275	GSM1974653	TNBC	9.668884984
GSE76275	GSM1974671	TNBC	9.649256178
GSE76275	GSM1974630	TNBC	8.005624549
GSE76275	GSM1974618	TNBC	9.465566405
GSE76275	GSM1974661	TNBC	9.224001674
GSE76275	GSM1974732	TNBC	8.562242424
GSE76275	GSM1974641	TNBC	7.330916878
GSE76275	GSM1974711	TNBC	7.948367232
GSE76275	GSM1974673	TNBC	7.483815777
GSE76275	GSM1974751	TNBC	5.754887502
GSE76275	GSM1974588	TNBC	7.942514505
GSE76275	GSM1974749	TNBC	8.6794801
GSE76275	GSM1974574	TNBC	7.483815777
GSE76275	GSM1974598	TNBC	8.479780264
GSE76275	GSM1974628	TNBC	9.640244936
GSE76275	GSM1974693	TNBC	9.47370575
GSE76275	GSM1974591	TNBC	8.668884984
GSE76275	GSM1974708	TNBC	9.269126679
GSE76275	GSM1974669	TNBC	9.539158811
GSE76275	GSM1974650	TNBC	9.087462841
GSE76275	GSM1974702	TNBC	9.813781191
GSE76275	GSM1974705	TNBC	9.296916207
GSE76275	GSM1974629	TNBC	8.022367813
GSE76275	GSM1974692	TNBC	8.948367232
GSE76275	GSM1974694	TNBC	5.554588852
GSE76275	GSM1974710	TNBC	9.303780748
GSE76275	GSM1974607	TNBC	8.721099189
GSE76275	GSM1974613	TNBC	8.370687407
GSE76275	GSM1974646	TNBC	8.930737338
GSE76275	GSM1974668	TNBC	7.714245518
GSE76275	GSM1974700	TNBC	8.592457037
GSE76275	GSM1974731	TNBC	8.927777962
GSE76275	GSM1974667	TNBC	7.108524457
GSE76275	GSM1974704	TNBC	9.116343961
GSE76275	GSM1974616	TNBC	6.700439718
GSE76275	GSM1974568	TNBC	7.159871337
GSE76275	GSM1974747	TNBC	9.398743692
GSE76275	GSM1974582	TNBC	9.044394119
GSE76275	GSM1974697	TNBC	9.627533884

GSE76275	GSM1974624	TNBC	9.887220615
GSE76275	GSM1974619	TNBC	9.413627929
GSE76275	GSM1974762	TNBC	6.06608919
GSE76275	GSM1974636	TNBC	9.105908509
GSE76275	GSM1974713	TNBC	9.060695932
GSE76275	GSM1974682	TNBC	8.430452552
GSE76275	GSM1974714	TNBC	6.700439718
GSE76275	GSM1974670	TNBC	8.997179481
GSE76275	GSM1974665	TNBC	8.366322214
GSE76275	GSM1974666	TNBC	8.108524457
GSE76275	GSM1974622	TNBC	8.379378367
GSE76275	GSM1974638	TNBC	7.672425342
GSE76275	GSM1974639	TNBC	6.129283017
GSE76275	GSM1974679	TNBC	9.764871591
GSE76275	GSM1974593	TNBC	8.682994584
GSE76275	GSM1974577	TNBC	9.828136484
GSE76275	GSM1974603	TNBC	8.816983623
GSE76275	GSM1974675	TNBC	9.911391988
GSE76275	GSM1974595	TNBC	9.654636029
GSE76275	GSM1974633	TNBC	8.071462363
GSE76275	GSM1974686	TNBC	8.693486957
GSE76275	GSM1974738	TNBC	7.14974712
GSE76275	GSM1974724	TNBC	9.668884984
GSE76275	GSM1974718	TNBC	8.832890014
GSE76275	GSM1974755	TNBC	8.581200582
GSE76275	GSM1974699	TNBC	7.906890596
GSE76275	GSM1974605	TNBC	8.912889336
GSE76275	GSM1974758	TNBC	9.818582177
GSE76275	GSM1974592	TNBC	9.11113567
GSE76275	GSM1974690	TNBC	8.118941073
GSE76275	GSM1974742	TNBC	8.997179481
GSE76275	GSM1974750	TNBC	8.607330314
GSE76275	GSM1974617	TNBC	8.965784285
GSE76275	GSM1974634	TNBC	8.299208018
GSE76275	GSM1974655	TNBC	7.851749041
GSE76275	GSM1974683	TNBC	8.912889336
GSE76275	GSM1974746	TNBC	9.002815016
GSE76275	GSM1974586	TNBC	9.556506055
GSE76275	GSM1974678	TNBC	9.665335917
GSE76275	GSM1974642	TNBC	8.611024797
GSE76275	GSM1974760	TNBC	7.813781191
GSE76275	GSM1974584	TNBC	7.451211112
GSE76275	GSM1974737	TNBC	8.754887502
GSE76275	GSM1974649	TNBC	9.008428622
GSE76275	GSM1974611	TNBC	8.876516947
GSE76275	GSM1974761	TNBC	9.505811554

GSE76275	GSM1974698	TNBC	10.01541505
GSE76275	GSM1974726	TNBC	8.383704292
GSE76275	GSM1974759	TNBC	8.724513853
GSE76275	GSM1974585	TNBC	7.977279923
GSE76275	GSM1974763	TNBC	5.64385619
GSE76275	GSM1974594	TNBC	8.033423002
GSE76275	GSM1974677	TNBC	8.353146825
GSE76275	GSM1974676	TNBC	6.357552005
GSE76275	GSM1974730	TNBC	7.169925001
GSE76275	GSM1974644	TNBC	5.754887502
GSE76275	GSM1974674	TNBC	6.857980995
GSE76275	GSM1974643	TNBC	6.832890014
GSE76275	GSM1974645	TNBC	6.832890014
GSE76275	GSM1974580	TNBC	5.247927513
GSE76275	GSM1974570	TNBC	8.479780264
GSE76275	GSM1974575	TNBC	6.426264755
GSE76275	GSM1974733	TNBC	5.491853096
GSE76275	GSM1974684	TNBC	8.194756854
GSE76275	GSM1974672	TNBC	8.550746785
GSE76275	GSM1974573	TNBC	4.807354922
GSE76275	GSM1974576	TNBC	5.044394119
GSE76275	GSM1974743	TNBC	9.566054038
GSE76275	GSM1974632	TNBC	6.303780748
GSE76275	GSM1974606	TNBC	7.357552005
GSE76275	GSM1974736	TNBC	5.087462841
GSE76275	GSM1974723	TNBC	6.169925001
GSE76275	GSM1974725	TNBC	6.614709844
GSE76275	GSM1974608	TNBC	8.366322214
GSE76275	GSM1974596	TNBC	6.768184325
GSE76275	GSM1974590	TNBC	6.044394119
GSE76275	GSM1974756	TNBC	7.839203788
GSE76275	GSM1974571	TNBC	8.930737338
GSE76275	GSM1974734	TNBC	5.491853096
GSE76275	GSM1974609	TNBC	7.366322214
GSE76275	GSM1974757	TNBC	6.965784285
GSE76275	GSM1974599	TNBC	7.658211483
GSE76275	GSM1974610	TNBC	7.17990909
GSE76275	GSM1974637	TNBC	9.303780748
GSE76275	GSM1974728	TNBC	7.721099189
GSE76275	GSM1974601	TNBC	8.055282436
GSE76275	GSM1974716	TNBC	5.857980995
GSE76275	GSM1974572	TNBC	8.77807713
GSE76275	GSM1974715	TNBC	7.700439718
GSE76275	GSM1974600	TNBC	7.942514505
GSE76275	GSM1974691	TNBC	8.603626345
GSE76275	GSM1974640	TNBC	9.25502857

GSE76275	GSM1974604	TNBC	9.247927513
GSE76275	GSM1974659	TNBC	9.594324604
GSE76275	GSM1974660	TNBC	9.211888295
